# Gene expression profiling of rubella virus infected primary endothelial cells of fetal and adult origin

**DOI:** 10.1186/s12985-016-0475-9

**Published:** 2016-02-02

**Authors:** Henriette Geyer, Michael Bauer, Jennifer Neumann, Amy Lüdde, Paul Rennert, Nicole Friedrich, Claudia Claus, Ludmilla Perelygina, Annette Mankertz

**Affiliations:** Division 12, “Measles, Mumps, Rubella, and Viruses Affecting Immunocompromised Patients”, Robert Koch Institute, 13353 Berlin, Germany; Institute of Molecular Life Sciences, University of Zurich, 8057 Zurich, Switzerland; Unit “Diagnostics and Pathogen Characterisation”, Bundesinstitut für Risikobewertung, 12277 Berlin, Germany; Institut für Virologie, Universität Leipzig, Johannisallee 30, 04103 Leipzig, Germany; Division of Viral Diseases, Centers for Disease Control and Prevention, 1600 Clifton Rd, Atlanta, GA 30333 USA

**Keywords:** Rubella virus, Congenital rubella syndrome, Gene expression, Sensory organ development, Teratogenicity, Endothelial cells

## Abstract

**Background:**

Rubella virus (RV) infection is usually a mild illness in children and adults. However, maternal infection during the first trimester of pregnancy can lead to congenital rubella syndrome (CRS) in the infant. Fetuses with CRS show damage to the endothelium of the heart and blood vessels; thus, it has been speculated that the clinical manifestations associated with CRS may be a result of endothelial cells persistently infected with RV. Here, we compared the effects of RV infection on gene expression in primary endothelial cells of fetal (HUVEC) and of adult (HSaVEC) origin by transcriptional profiling.

**Results:**

More than 75 % of the genes differentially regulated following RV infection were identical in both cell types. Gene Ontology (GO) analysis of these commonly regulated genes showed an enrichment of terms involved in cytokine production and cytokine regulation. Increased accumulation of inflammatory cytokines following RV infection was verified by protein microarray. Interestingly, the chemokine CCL14, which is implicated in supporting embryo implantation at the fetal-maternal interface, was down-regulated following RV infection only in HUVEC. Most noticeably, when analyzing the uniquely regulated transcripts for each cell type, GO term-based cluster analysis of the down-regulated genes of HUVEC revealed an enrichment of the GO terms “sensory organ development”, “ear development” and “eye development”.

**Conclusion:**

Since impairment in vision and hearing are the most prominent clinical manifestations observed in CRS patients, the here detected down-regulated genes involved in the development of sensory organs sheds light on the molecular mechanisms that may contribute to the teratogenic effect of RV.

**Electronic supplementary material:**

The online version of this article (doi:10.1186/s12985-016-0475-9) contains supplementary material, which is available to authorized users.

## Background

Rubella virus (RV) is a single-stranded RNA virus of positive polarity and belongs to the family *Togaviridae*. RV infection typically causes mild symptoms such as rash and fever in children and adults; however, its teratogenicity is still a public health concern. Maternal infection early in pregnancy can lead to a combination of birth defects in infants, collectively called congenital rubella syndrome (CRS) [1, 2]. Transplacental transmission of the virus is high in the first weeks of gestation [3], and if infection occurs, the virus is able to infect almost any organ, thereby establishing a chronic infection in the developing fetus [4]. In RV-infected embryos and fetuses, vascular abnormalities, such as lesions in the endothelium of the heart and blood vessels, have been described as the most frequently observed pathological findings [5–8]. Common clinical manifestations associated with CRS are patent ductus arteriosus, pulmonary artery stenosis and valvular stenosis [9]. Other frequently observed defects include cataracts, glaucoma, sensorineural deafness and psychomotor retardation [10]. Despite the introduction of an effective vaccine and global vaccination programs, approximately 110,000 CRS cases are estimated to still occur worldwide per year [11].

RV infection during the critical stages of organ development and in a setting where a mature immune system is absent is believed to account for the clinical manifestations of CRS. However, the exact molecular mechanisms by which RV causes CRS are still poorly understood. Previous work has shown that RV interferes with cellular proliferative pathways, alters cytoskeletal structures, and induces mitochondrial changes, leading to the hypothesis that virus-induced apoptosis contributes to the teratogenic effects of RV (reviewed in J. Y. Lee and D. S. Bowden [12] and D. M. Knipe and P. M. Howley [13]). However, most of these findings are derived from studies using immortalized adult cell lines (e.g. Vero, A549, and BHK-21 cells). These cell lines differ considerably from fetal cells in vivo in terms of gene expression, metabolism, and growth rate, and therefore give only limited insight into the effects of RV infection during embryonic development.

Microarray gene expression analysis provides a valuable tool to comprehensively examine and identify pathways that are affected by virus infection. Previous transcriptional profiling analysis following RV infection of primary human fibroblasts derived from a whole embryo [14], as well as the ECV304 cell line which exhibit both endothelial and epithelial characteristics [15], revealed that RV induces a robust interferon-stimulated gene response. However, since endothelial cells are believed to play a major role in RV-induced teratogenesis, our studies focused on the gene expression changes of an infection caused by a wild type RV isolate (Wuerzburg-12) in primary fetal endothelial cells derived from the human umbilical vein (HUVEC) and adult endothelial cells derived from the human saphenous vein (HSaVEC). By comparing up- and down-regulated genes in the endothelial cells of fetal and adult origin using Gene ontology (GO) term analysis, we were able to identify differences in biological processes and pathways between HUVEC and HSaVEC. We believe that these differences in gene expression after infection of endothelial cells of adult and fetal origin provide new insights into the molecular mechanisms involved in RV-induced teratogenicity.

## Results

### Primary endothelial cells are permissive for RV

**Fig. 1:**
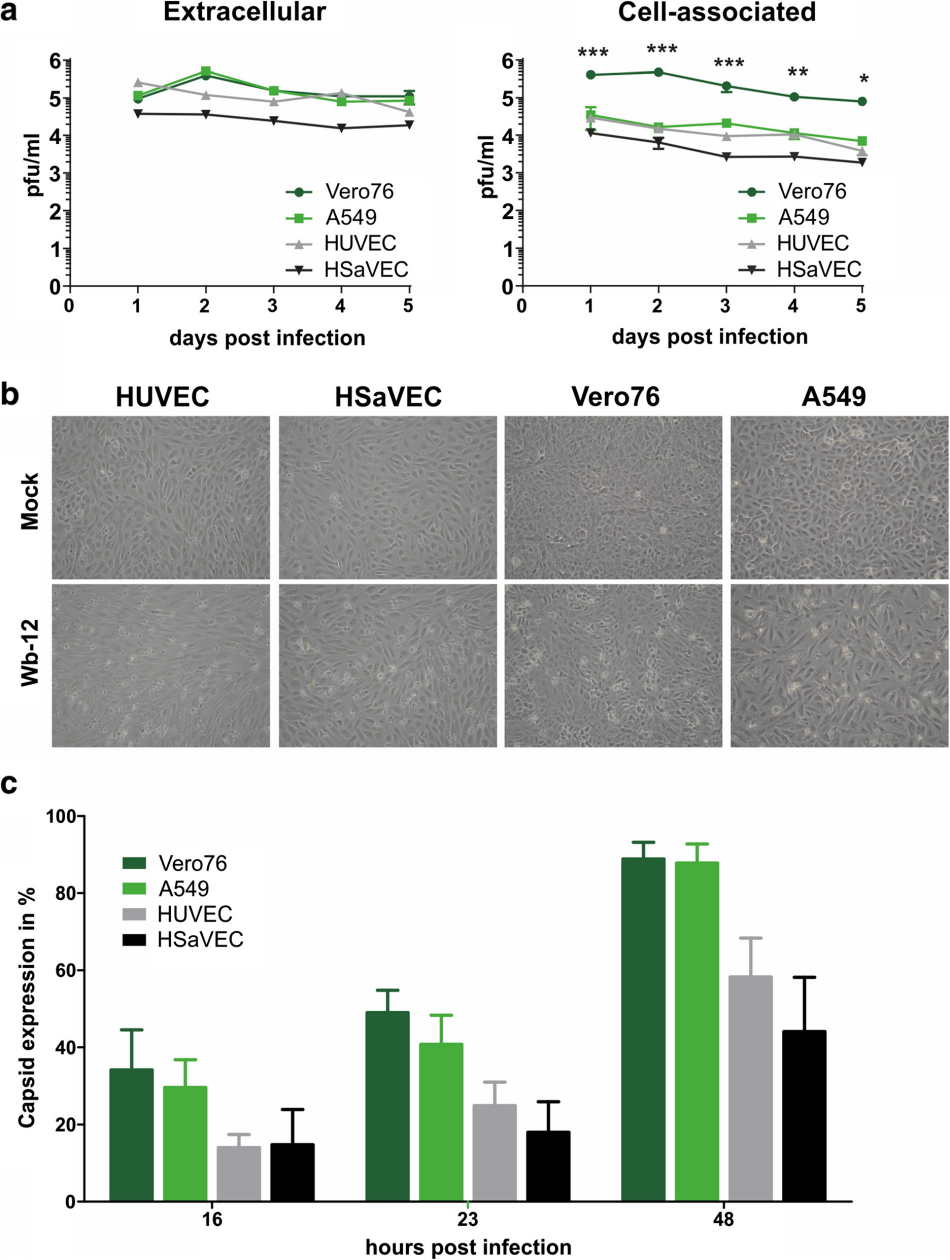
RV replication in primary endothelial cells and RV-permissive cell lines. **a** RV replication kinetics in HUVEC, HSaVEC, Vero76, and A549 cells. Cells were infected with RV at an MOI of 5. At the indicated time points, cell culture supernatants or cell lysates were titered in duplicate on Vero76 cells. The data are represented as the mean of two independent experiments ± standard deviation (SD). The data were analyzed by two-way ANOVA with the Bonferroni correction for multiple comparisons (*, p ≤ 0.05; **, p ≤ 0.01; ***, p ≤ 0.001). **b** Phase contrast pictures of non-infected cells or cells infected with RV with an MOI of 10 two dpi. **c** Capsid protein levels of RV-infected cell types at various time points post infection. Cells were infected with an MOI of 5 and harvested at the indicated time points and analyzed by flow cytometry. Intracellular staining was carried out using a capsid-specific antibody and anti-mouse-Cy5 serum as a secondary antibody. Non-infected cells served as the control. All experiments were performed three times. Error bars represent means ± SD

RV is characterized by a relatively slow replication cycle [16, 17] and has been shown to infect different cell types asynchronously, even at a high MOI [14, 18]. However, L. Perelygina, et al. [19] found that RV infection at an MOI ≥ 10 produced synchronously infected HUVEC cultures. To determine the optimal time point for gene expression analyses and in order to see if HSaVEC are also synchronously infected, viral replication was analyzed by growth curves and viral capsid protein levels were determined by flow cytometry. The immortalized cell lines Vero76 and A549 have been used successfully for producing high RV titers [13] and were therefore used as controls in the following experiments. Growth kinetics showed that RV effectively replicates in the monolayer of fetal and adult primary endothelial cells. Comparable titers were observed after infection of HUVEC, A549, and Vero76; the infection of HSaVEC produced slightly lower viral titers (Fig. 1(a)). However, the viral titer remained almost constant during the observation period of five days for all cell types. Since it has been shown that the efficiency of RV egress is strongly dependent on the cell line [20], we quantified the titers of both extracellular and cell-associated virus, revealing that a similar amount of extracellular and cell-associated virus was produced in Vero76 cells. In contrast, the amount of extracellular virus was approximately one log higher in the supernatant than cell-associated in HUVEC, HSaVEC, and A549 cells. This is in accord with previous studies and suggests a more efficient viral egress in human cell lines [19]. Only moderate cytopathic effects (CPE), such as cell rounding and detachment, were detectable for all cell types two days post infection (dpi) (Fig. 1 (b)).

In contrast to L. Perelygina, et al. [19] we did not detect synchronous infection in HUVEC by flow cytometry (Fig. 1 (c)). Approximately one fourth of the primary endothelial cells were RV capsid positive 23 h post infection (hpi). Capsid protein levels increased over time, reaching about 50 % of capsid positive cells in the primary endothelial cells 48 hpi. Interestingly, immortalized cell lines Vero76 and A549 cells expressed more viral capsid than HUVEC and HSaVEC, indicating that RV is able to infect these cell lines more efficiently.

### Similar changes in gene expression pattern after RV infection of fetal and adult endothelial cells

The viral life cycle of RV is completed approximately 24 hpi [16, 19]. Due to asynchronous infection of the primary endothelial cells, and in order to have a relatively high number of infected primary endothelial cells while still avoiding multiple viral life cycles, 36 hpi was chosen for analysis of gene expression. HUVEC and HSaVEC were infected in triplicate with the clinical isolate Wb-12 at an MOI of 10. Non-infected cells served as the control. Total RNA was harvested 36 hpi and was used for microarray analysis of host cell transcription. To compare the gene expression of infected and non-infected cells, ratios were calculated by using normalized signal intensities of infected- and non-infected cells. Genes that exhibited a fold change in gene expression ≥ 4 and ≤ −4 with an ANOVA p-value of ≤ 0.01 were chosen for further analysis (Additional file 1).

**Fig. 2:**
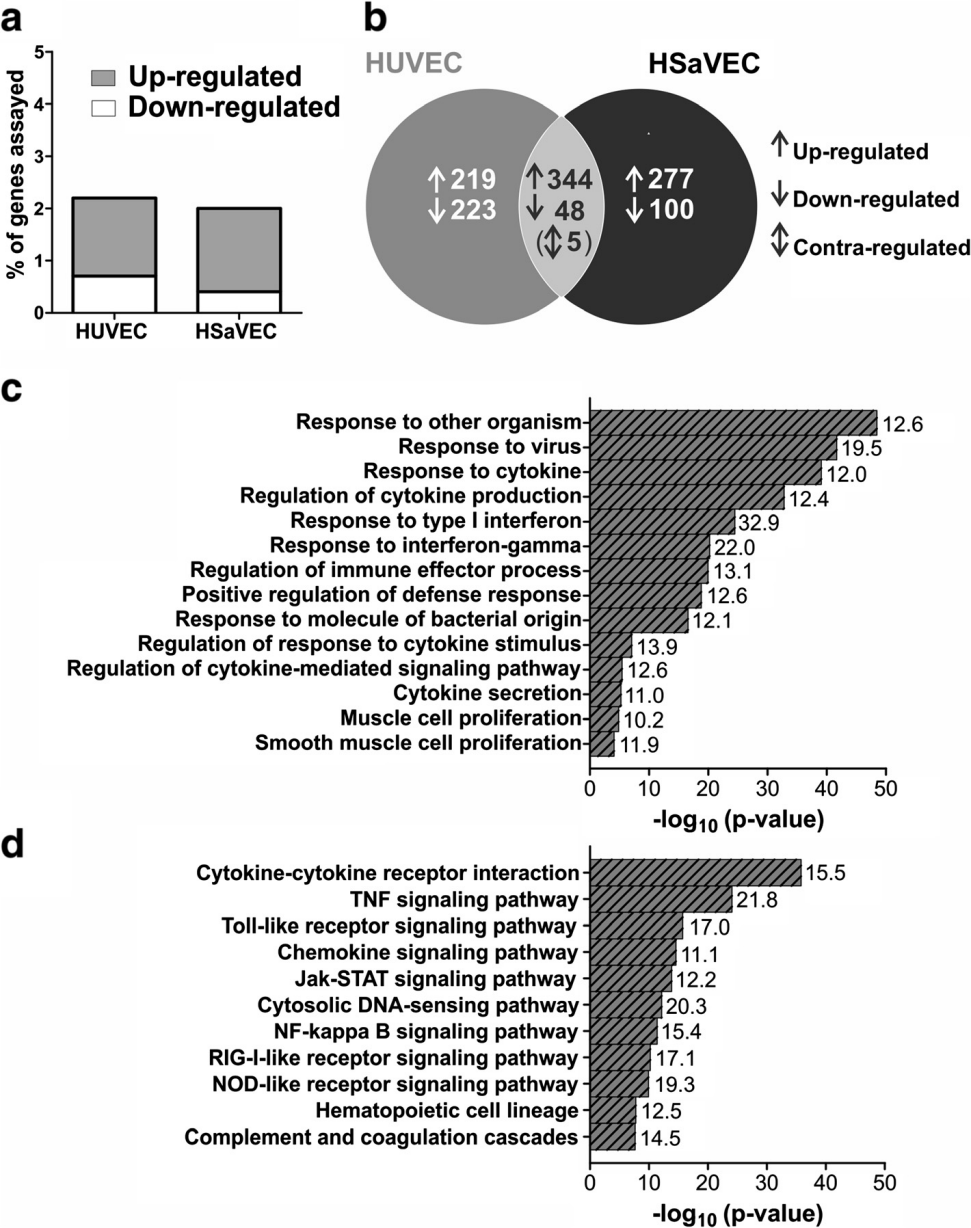
Common changes in cellular gene expression after RV infection in fetal and adult endothelial cells. **a** Displayed are the percent of genes that exhibited expression changes greater than or equal to 4-fold with an ANOVA p-value of ≤ 0.01. The percentage is based on the total amount of genes assayed. **b** Venn diagram showing the intersection between the up-regulated (↑), down-regulated (↓) and contra-egulated (↕) transcripts 36 h after RV infection (MOI of 10) of HUVEC and HSaVEC. **c** Biological process and **d** KEGG pathway analysis of the 392 transcripts that were affected after RV infection in both fetal and adult endothelial cells. Displayed are the ranked Bonferroni corrected p-values of significantly enriched biological processes and pathways (p ≤ 0.01). Related terms were grouped and the most significant term of the group was defined as the group leading term. Numbers at the bar represent genes (in %) from the cluster that were associated with the term

Gene expression data of virus-infected to non-infected cells were compared to each other to determine the relative modulation of cellular transcription induced by RV infection. Of the 38,500 human genes assayed, 834 (2.2 %) and 769 (2.0 %) genes were differentially regulated in HUVEC and HSaVEC, respectively (Fig. 2 (a)). In HUVEC, 563 genes were up-regulated and 271 were down-regulated following RV infection. Similarly, in HSaVEC, 621 genes were up-regulated and 148 were down-regulated. A comparison of the number of differentially regulated genes in both endothelial cells showed an overlap of more than 75 % (Fig. 2 (b)). Of the overlapping genes, five genes were contra-regulated. Remarkably, a larger proportion of genes were down-regulated in HUVEC after RV infection compared to HSaVEC.

Due to the high overlap of genes which are affected by RV infection in HUVEC and HSaVEC, we wanted to know whether certain biological processes and cellular pathways were statistically overrepresented. GO term-based analysis of the 392 commonly regulated genes was applied. As shown in Fig. 2 (c), fourteen biological processes were enriched in the endothelial cells. Remarkably, a large proportion of genes were assigned to GO terms involved in cytokine production and regulation. Furthermore, GO terms involved in cellular defense mechanisms were enriched in infected HUVEC and HSaVEC. In addition to GO term-analysis, KEGG pathway analysis (Fig. 2 (d)) revealed the enrichment of pathways which are involved in antiviral responses, such as the Toll-like receptor signaling pathway and the RIG-I-like receptor signaling pathway.

### Similar chemokine expression pattern after RV infection in fetal and adult endothelial cells

Chemokines are chemotactic cytokines that play an important role during inflammation and homeostasis. While inflammatory chemokines are required to recruit immune cells to the site of infection, homeostatic chemokines possess a key function in controlling cell migration during developmental processes. Interference with the precisely coordinated production of chemokines triggered by a viral infection has profound effects on fetal development and is speculated to contribute to the pathological findings of CRS [14].

**Fig. 3:**
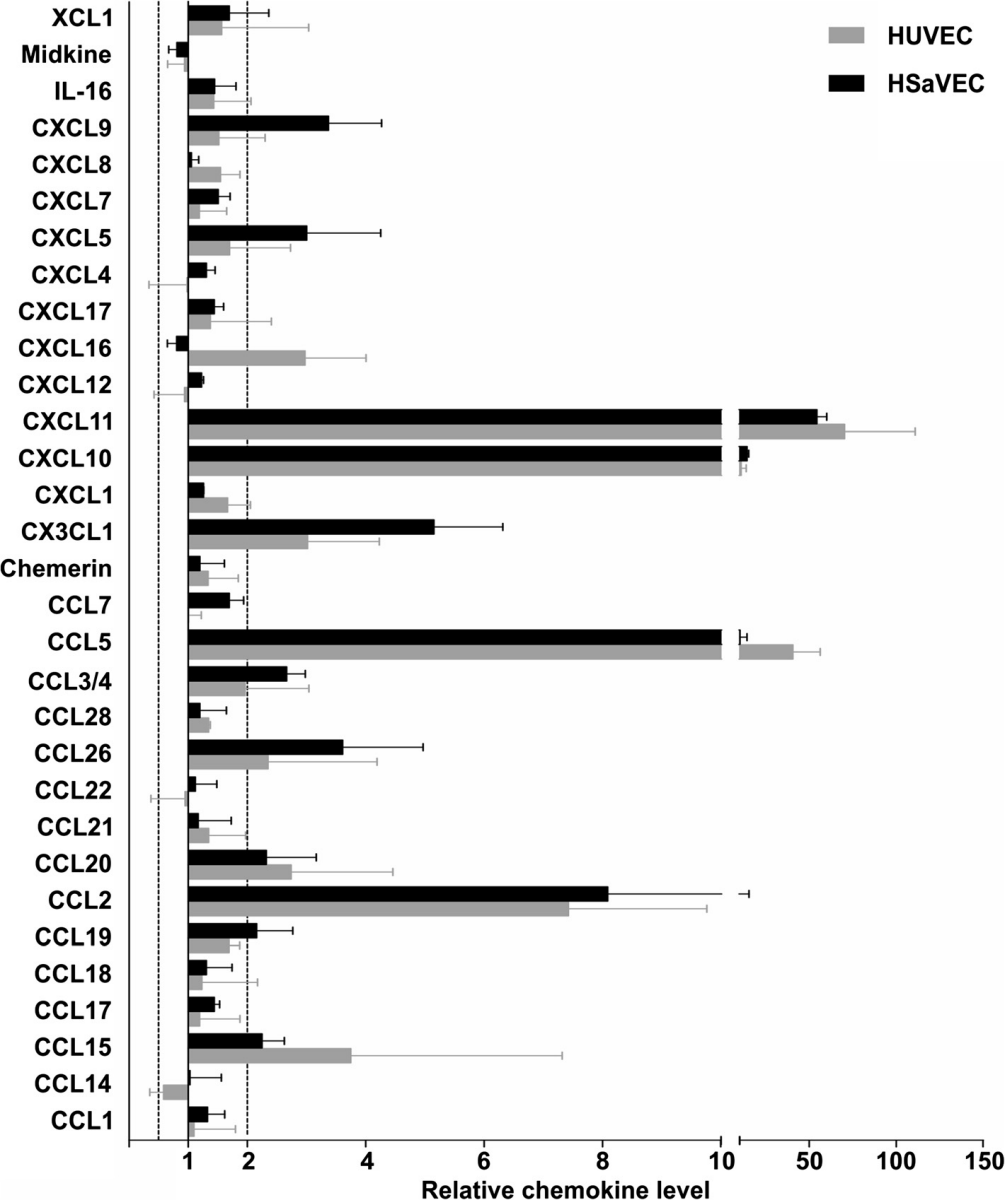
Impact of RV infection on chemokine secretion. HUVEC and HSaVEC were infected with RV at an MOI of 10 and the cell culture supernatant was collected 48 hpi. Chemokine levels in the supernatant were determined using a human chemokine array kit. Changes in chemokine concentration after RV infection are shown as the fold change compared to uninfected cells. Dotted lines indicate the thresholds that were chosen for the experiment (fold change ≥ 2 and ≤ 0.5). Data are represented as the mean of two independent experiments ± SD

Our GO term analysis revealed that biological processes involved in cytokine production, regulation, and processing are enriched in both adult and fetal primary endothelial cells. Moreover, gene expression was strongly increased for the interferon-inducible chemokines (Table 1). To verify up-regulation of these chemokines on the protein level, HUVEC and HSaVEC were infected at an MOI of 10 with RV and the chemokine levels in the supernatant were quantified with a protein array at 48 hpi (Fig. 3). Similar to the microarray results, significantly increased amounts of CCL2, CCL3, CCL4, CCL5, CCL15, CCL20, CCL26, CXCL10, CXCL11 and CX3CL1 were detected in the supernatant following RV infection in both HUVEC and HSaVEC. The overall pattern of chemokine production before and after infection was similar in the endothelial cells (Additional file 2). Interestingly, one chemokine, CCL14, appeared to be secreted at significantly lower amounts into the supernatant following RV infection of HUVEC.

**Table 1:** Differentially expressed genes of the chemokine family

Gene symbol	Gene title	Mean fold change^(a)^	
		HUVEC	HSaVEC
*CCL2*	chemokine (C-C motif) ligand 2	21.03	n.s.
*CCL20*	chemokine (C-C motif) ligand 20	112.23	122.14
*CCL23*	chemokine (C-C motif) ligand 23	4.32	4.09
*CCL3, CCL3L1, CCL3L3*	chemokine (C-C motif) ligand 3; chemokine (C-C motif) ligand 3-like 1; chemokine (C-C motif) ligand 3-like 3	194.09	278.09
*CCL4*	chemokine (C-C motif) ligand 4	231.15	972.27
*CCL5*	chemokine (C-C motif) ligand 5	1394.87	430.3
		1254.72	325.72
		414.38	310.81
*CCL7*	chemokine (C-C motif) ligand 7	115.92	n.s.
*CCL8*	chemokine (C-C motif) ligand 8	579.29	151.42
*CX3CL1*	chemokine (C-X3-C motif) ligand 1	23.37	36.99
		12.43	28.53
*CXCL1*	chemokine (C-X-C motif) ligand 1	5.59	n.s.
*CXCL10*	chemokine (C-X-C motif) ligand 10	4833.33	297.78
*CXCL11*	chemokine (C-X-C motif) ligand 11	382.38	72.25
*CXCL12*	chemokine (C-X-C motif) ligand 12	35.66	n.s.
		7.43	
*CXCL16*	chemokine (C-X-C motif) ligand 16	8.68	6.29
*CXCL2*	chemokine (C-X-C motif) ligand 2	28.56	4.70
		25.62	4.18
		22.74	
*CXCL3*	chemokine (C-X-C motif) ligand 3	92.97	33.14
*CXCL5*	chemokine (C-X-C motif) ligand 5	44.83	15.34
		22.63	12.55
			12.1
*CXCL6*	chemokine (C-X-C motif) ligand 6	84.27	8.97
*CXCL9*	chemokine (C-X-C motif) ligand 9	41.56	88.71

^(a)^Displayed are average fold change of RV-infected cells in comparison to non-infected cells detected by transcriptome analysis. In positions that are labeled n.s., the gene expression did not meet the selected criteria (i.e. fold change cut-off ≤ −4 and ≥ 4 and ANOVA p-value of ≤ 0.01). If a gene was detected by several different probes, all fold changes that meet the selected criteria are shown

### Identification of differentially regulated genes induced by RV in fetal and adult cells

**Fig. 4:**
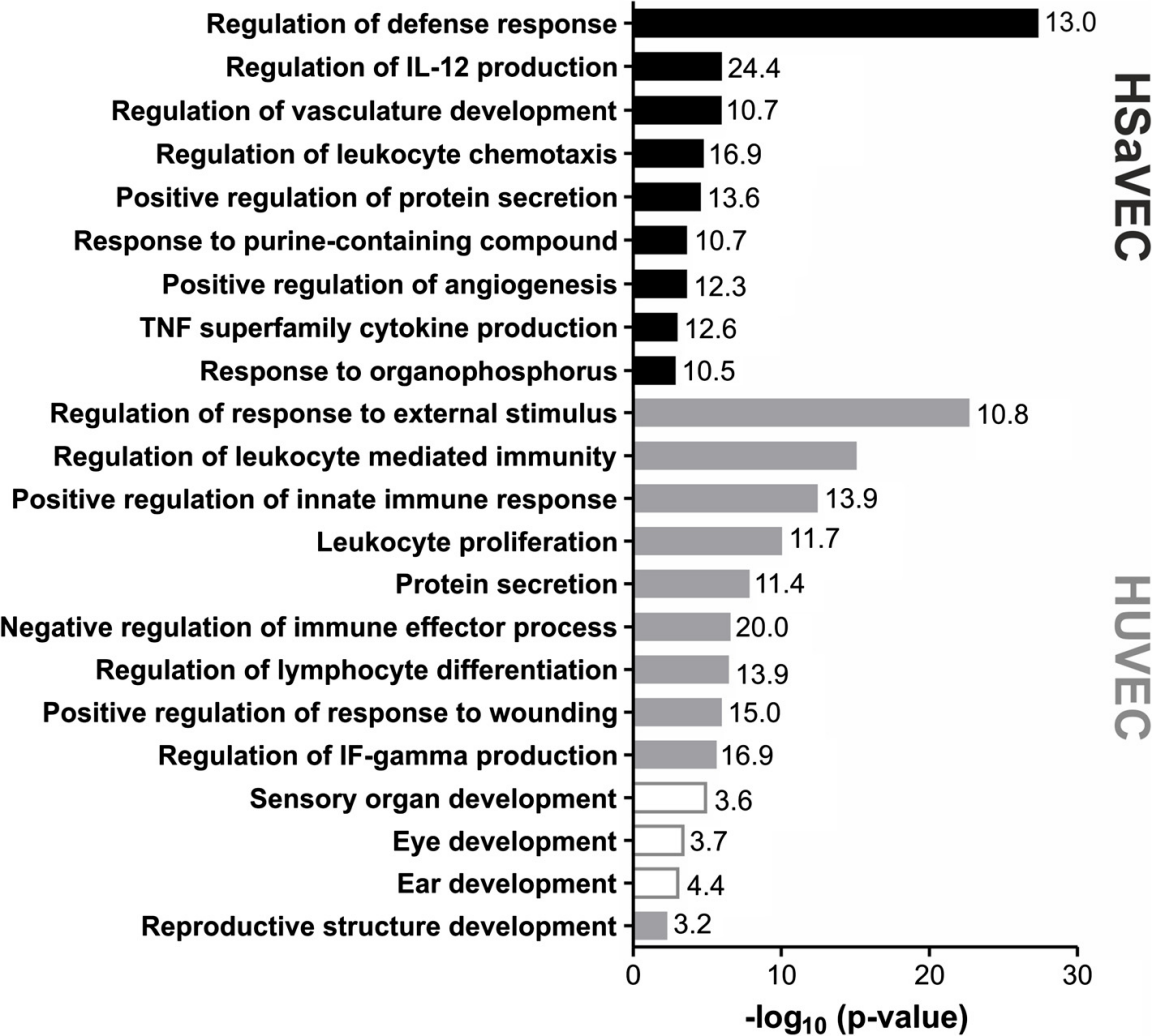
Bonferroni corrected p-values of significantly enriched biological processes which are unique to the two primary cells following RV infection. Related terms were merged into functional groups and the most significant term was defined as the group-leading term. Numbers at the bar represent genes (in %) from the cluster that were associated with the term. Unfilled bars indicate if the GO term is derived from the down-regulated gene list of HUVEC. Filled bars indicate if the GO term is derived from the up-regulated gene list of HUVEC (grey) and HSaVEC (black)

In order to identify biological processes which are differentially regulated after RV infection of primary cells of adult and fetal origin, GO term-based cluster analysis was carried out with genes that were up- or down-regulated following infection compared to non-infected cells. Figure 4 shows the GO terms that were significantly (p ≤ 0.01) over-represented in the set of genes that were up- or down-regulated after RV infection of HUVEC and HSaVEC. Displayed are the Bonferroni corrected p-values, with the numbers at the bar representing the percentage of genes from the cluster that were associated with the term (compared with all genes associated with the term). In addition to genes involved in immune defense processes, genes associated with angiogenesis and vascular development were over-represented following RV infection in the up-regulated gene list of HSaVEC. No GO terms were over-represented in the down-regulated gene list of HSaVEC. In the set of genes up-regulated in HUVEC following RV infection, GO terms involved in immune processes were over-represented. Moreover, the GO term “negative regulation of immune effector processes” was also over-represented in this set of genes, indicating that genes are activated upon infection that counteract the cellular immune response. Interestingly, 18 genes assigned to the GO term “sensory organ development”, “eye development”, and “ear development” were over-represented in the set of down-regulated genes of HUVEC, but not HSaVEC. Heart malformation, deafness and ocular conditions such as cataracts are the most common clinical manifestations of CRS, however the molecular mechanisms that lead to the pathological conditions (or the gene products involved) are not known. Table 2 summarizes the fold changes of the down-regulated sensory organ development genes as detected by microarray analysis for HUVEC and HSaVEC. Four of the 18 genes that were down-regulated in HUVEC were also down-regulated in HSaVEC. The remaining 14 genes were either unaffected or up-regulated following infection in these cells.

**Table 2:** Differential expression of genes belonging to the GO term “sensory organ development” in HUVEC and HSaVEC following RV infection

Gene symbol	Gene title	Fold change microarray ^(a)^	
		HUVEC	HSaVEC
*ADAMTS18*	ADAM metallopeptidase with thrombospondin type 1 motif, 18	−4.89	n.s.
*ALDH1A2*	aldehyde dehydrogenase 1 family, member A2	−4.16	n.s.
*CLN8*	ceroid-lipofuscinosis, neuronal 8	−8.05	n.s.
*DSCAM*	Down syndrome cell adhesion molecule	−9.98	n.s.
*FGFR2*	fibroblast growth factor receptor 2	−10.01	5.03
*FZD3*	frizzled family receptor 3	−10.00	−8.56
*JAG2*	jagged 2	−4.86	n.s.
		−4.01	
*MYO3A*	myosin 3A	−9.46	n.s.
*MYO7A*	myosin 7A	−5.65	−6.17
*NHS*	Nance-Horan syndrome	−4.35	n.s.
*NOG*	noggin	−6.68	−5.04
*NTRK3*	neurotrophic tyrosine kinase, receptor, type 3	−7.70	n.s.
*PDGFRA*	platelet-derived growth factor receptor, alpha polypeptide	−6.24	n.s.
*RDH10*	retinol dehydrogenase 10 (all-trans)	−9.00	n.s.
*SLC25A27*	solute carrier family 25, member 27	−16.78	n.s.
*TNPO1*	transportin 1	−7.66	n.s.
*TRPM1*	transient receptor potential cation channel, subfamily M, member 1	−7.72	−5.76
*TSPAN12*	tetraspanin 12	−4.42	n.s.

^a^Displayed are the average fold changes from three independent experiments of RV-infected cells in comparison to non-infected cells detected by the microarray that meet the selected criteria (i.e. fold change cut-off ≤ −4 and ≥ 4 and ANOVA p-value of ≤ 0.01). If a gene was detected by several different probes, all fold changes that meet the selected criteria are shown. Positions labeled n.s. where not shown to be significantly up- or down-regulated in the DNA microarray analysis

**Fig. 5:**
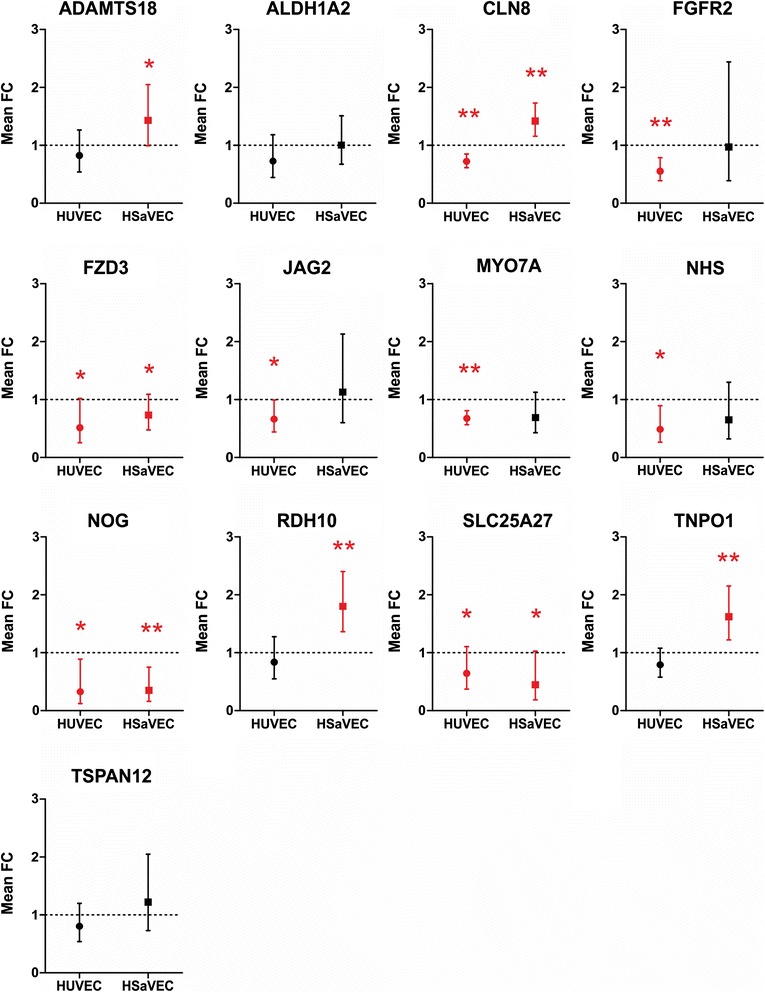
Differential expression of genes belonging to the GO term “sensory organ development” in HUVEC and HSaVEC following RV infection as determined by qPCR. HUVEC and HSaVEC from different donors were infected with RV at an MOI of 5 and gene expression relative to non-infected cells was quantified by qPCR 36 hpi. *GAPDH* was used for normalization. Bars denote mean fold change (FC, n = 5 for HUVEC and n = 6 for HSaVEC) with confidence intervals calculated by the ∆∆CT mathematical model. A one-tailed paired *t*-test was used to determine statistically significant differences in gene expression compared to non-infected cells using ∆CT values and is indicated by an asterisk and red coloring (*, p ≤ 0.05; **, p ≤ 0.01)

In order to elucidate the putative molecular processes leading to pathogenesis, we sought to verify the down-regulated expression of genes assigned to the GO term “sensory organ development” by qPCR. Therefore, HUVEC and HSaVEC were infected with Wb-12 at an MOI of ten and total RNA was isolated 36 hpi and subjected to qPCR. For HUVEC, two pools of cells were utilized for five independent experiments; whereas for HSaVEC, two donors were used for six independent experiments. Non-infected cells served as the control and *GAPDH* was used for normalization. Analysis of gene expression by qPCR was carried out with 13 genes according to the ∆∆CT method. Of the 13 genes belonging to this GO term, qPCR was able to confirm the down-regulation of *CLN8*, *FGFR2*, *FZD3*, *JAG2*, *MYO7A*, *NHS*, *NOG* and *SLC25A27* in infected HUVEC (Fig. 5). Down-regulation was verified for *FZD3*, *NOG* and *SLC25A27* in infected HSaVEC; the other ten sensory organ development genes were either up-regulated or not affected in this cell type.

Differences in gene expression between HUVEC and HSaVEC following RV infection might arise due to significant differences in basal gene expression. In order to rule this out for the selected sensory organ development genes, the basal expression levels of non-infected endothelial cells were compared with each other (Table 3). Based on our selected criteria (i.e. fold change cut-off ≤ −4 and ≥ 4 and ANOVA p-value of ≤ 0.01) only the basal gene expression level of *ADAMTS18* was significantly lower and *NHS* was significantly higher in HUVEC compared to HSaVEC. Thus, we cannot completely rule out for these genes that the observed differences following RV infection between the primary endothelial cells might be the result of differences in basal expression rates. However, for the remaining eleven other genes examined, no differences in basal gene expression were seen.

**Table 3:** Basal expression of genes belonging to the GO term “sensory organ development” in HUVEC and HSaVEC

Gene symbol	Gene title	Fold Change HUVEC vs HSaVEC^(a)^	ANOVA p-value HUVEC vs HSaVEC^(b)^
*ADAMTS18*	ADAM metallopeptidase with thrombospondin type 1 motif, 18	−5.87	0.000027
*NHS*	Nance-Horan syndrome	5.43	0.000766

^(a^)Displayed are the average fold changes from three independent experiments of non-infected HUVEC in comparison to non-infected HSaVEC detected by microarray that meet the selected criteria (i.e. fold change cut-off ≤ −4 and ≥ 4 and ANOVA p-value of ≤ 0.01)

^(b)^ANOVA p-values ≤ 0.01 are considered significant

**Fig. 6:**
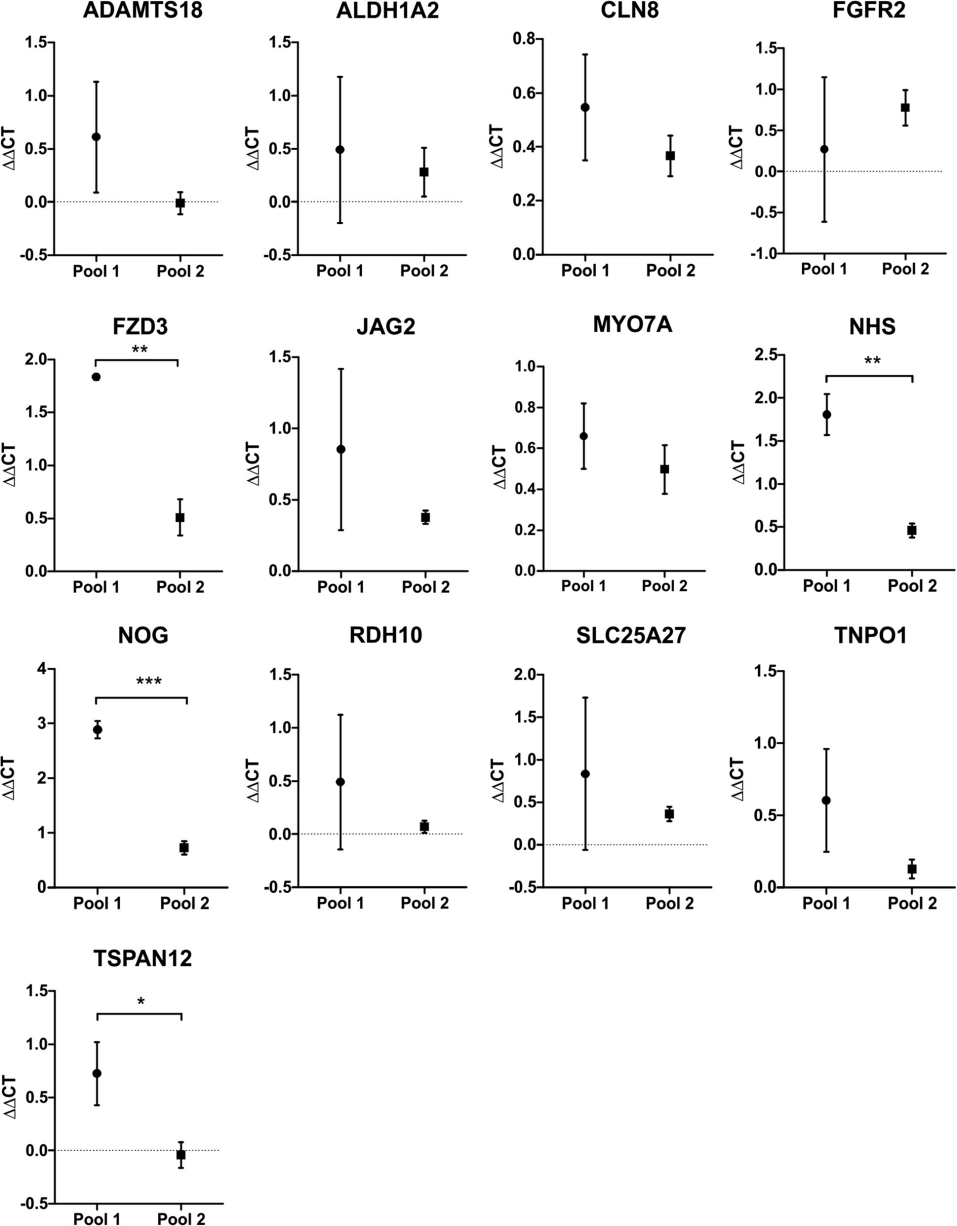
Donor-to-donor variation of sensory organ gene expression in HUVEC isolated from different donors. Two pools of HUVEC, with each pool consisting of cells from three different donors, were infected with RV at an MOI of 5 and gene expression relative to non-infected cells was quantified by qPCR 36 hpi. *GAPDH* was used for normalization. Bars denote mean ∆∆CT ± SEM with ∆∆CT values < 0 indicating up-regulation and ∆∆CT values > 0 indicating down-regulation. A two-tailed unpaired *t*-test was used to determine statistically significant differences in gene expression using ∆CT values and is indicated by an asterisk (*, p ≤ 0.05; **, p ≤ 0.01)

Primary cells are thought to be a good model for studying viral infections. However, these cells have been shown to exhibit significant donor-to-donor variability in some biological aspects. Although two pools of HUVEC and HSaVEC from two different donors were used for qPCR, we wanted to ensure that the observed effects are not unique to a certain donor. Thus, we compared the levels of sensory organ gene expression following RV infection with cells from different donors. Fig. 6 compares the ∆∆CT values obtained from infections with two pools of HUVEC — with each pool of cells consisting of three different donors. The expression of *FZD3*, *NHS*, *NOG* and *TSPAN12* differed significantly between the two pools of cells following infection. Despite the significant difference, the direction of regulation (i.e. down-regulation) was the same for *FZD3*, *NHS* and *NOG* between the pools of cells. Thus, down-regulation of the sensory organ development genes could be observed for the majority of genes in HUVEC derived from different donors, suggesting that the observed effects on expression were not donor specific.

**Fig. 7:**
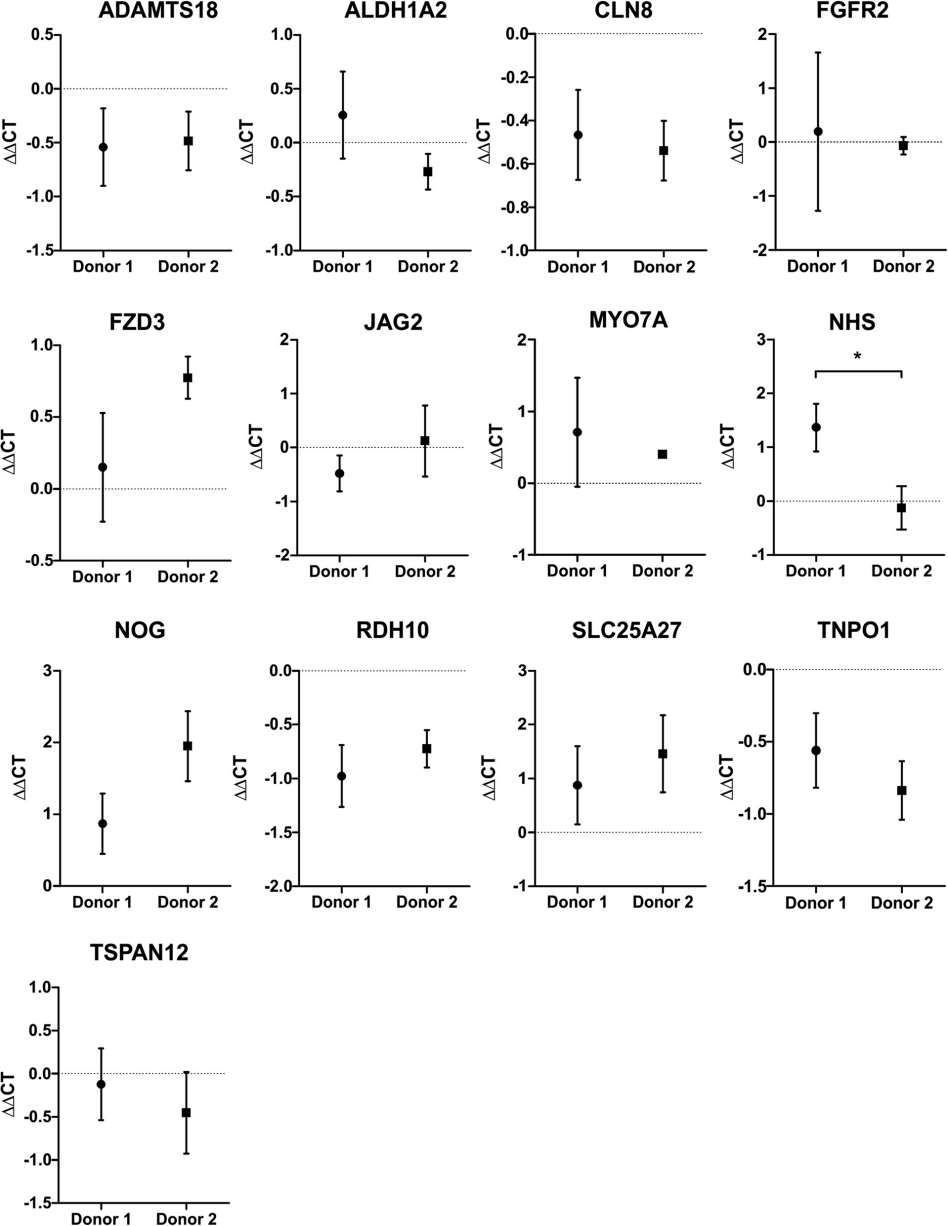
Donor-to-donor variation of sensory organ gene expression in HSaVEC isolated from two different donors. HSaVEC obtained from two different donors were infected with RV at an MOI of 5 and gene expression relative to non-infected cells was quantified by qPCR 36 hpi. *GAPDH* was used for normalization. Bars denote mean ∆∆CT ± SEM with ∆∆CT values < 0 indicating up-regulation and ∆∆CT values > 0 indicating down-regulation. A two-tailed unpaired *t*-test was used to determine statistically significant differences in gene expression using ∆CT values and is indicated by an asterisk (*, p ≤ 0.05)

Since pooled HSaVEC are not commercially available and the number of donors is limited, the expression changes for two individual donors were compared and showed a significant difference between donor 1 and 2 for *NHS*. However, the remaining twelve sensory organ development genes did not differ between the two donors following RV infection (Fig. 7).

## Discussion

Cardiovascular abnormalities, sensorineural hearing loss, cataracts, and glaucoma have been the most prominent findings among patients suffering from CRS [8]. Although RV has been shown to efficiently replicate in endothelial cells [19], and was also found in the endothelium of infected fetuses [6, 7, 9], studies with human endothelial cells are scarce. Most research was conducted with non-human cell lines, such as Vero, BHK and RK13.

Since it has been speculated that endothelial cells play an important role in the birth defects associated with CRS, we wanted to explore the effect of RV infection on primary human endothelial cells of adult and fetal origin by gene expression analysis. HUVEC are considered fetal cells because they are derived from the umbilical cord veins, which develop genetically and physiologically from the fetus. In contrast to that, HSaVEC are derived from the saphenous vein of adult donors. By analyzing the effects of RV replication on fetal and adult endothelial cells we approched to understand the mechanisms underlying RV-induced teratogenesis.

Efficient replication of RV in endothelial cells was previously reported for HUVEC [19], but not for HSaVEC. Therefore, the primary endothelial cells were analyzed for their ability to support viral replication, in addition to the Vero76 and A549 cell lines (which have been routinely used to cultivate RV). The replication kinetics and the detection of viral proteins showed that RV replicates both in fetal and adult primary endothelial cells. RV infection of HUVEC resulted in slightly lower extracellular titers compared to the infection of the immortalized cell lines Vero76 and A549. The extracellular titers after infection of HSaVEC were about 10-fold reduced, which might be caused by the slower growth rate of this cell line. However, viral titers remained almost constant for the observation period of five days after infection with an MOI of five, indicating that the virus caused a persistent infection in the immortalized cell lines as well as in the primary endothelial cells. The results of the capsid expression analysis show that the immortalized cell lines Vero76 and A549 are infected more efficiently by RV than HUVEC and HSaVEC by a factor of almost two. Synchronous infection has not been observed in any of the cell lines after infection at an MOI of 5, which is consistent with findings in other various cell lines [14, 17, 18, 21]. In one other study, infection of HUVEC with a clinical isolate of a different genotype (1E) has been shown to be synchronous at a high MOI [19]. Possibly, different RV strains may have distinct efficiencies and properties regarding infection or different cellular tropisms. Since RV-infected HUVEC and HSaVEC with similar efficiencies, it is reasonable to assume that the virus generally does not have a stronger tropism for fetal cells than for adult cells.

The aim of the present study was to identify differences in the gene expression pattern following RV infection in fetal and adult primary cells in order to understand the teratogenicity of the virus. More than 75 % of the differentially regulated genes following RV infection overlapped between HUVEC and HSaVEC cells indicating similar gene expression modulation following RV infection. Not surprisingly, these genes were grouped to GO terms involved in host defense mechanisms, such as cytokine regulation and production. M. P. Adamo, et al. [14] and X. Y. Mo, et al. [15] reported a robust interferon-stimulated gene response following infection of human embryonic fibroblasts and the ECV304 cell line, respectively. This is corroborated by our findings, since expression of interferon-inducible genes, such as *OASL* and *MX2*, was strongly upregulated following RV infection. Moreover, these genes showed the highest ranking position when the commonly expressed genes of HUVEC and HSaVEC were analyzed (Additional file 3).

It has been speculated that dysregulated expression of cytokines and chemokines following RV infection disrupts cellular processes in the developing fetus, thereby contributing to CRS. However, data on which chemokines are produced during RV infection and how they affect the developing fetus are missing. Using the primary endothelial cell model system, we examined whether increased expression of chemotactic cytokines was detected on the protein level by a chemokine array. In concordance with the microarray results, an increase of inflammatory chemokines was detected in the supernatant of the RV-infected endothelial cells. Interestingly, CCL5, CXCL10, and CXCL11 — which have been reported to play a key role in the pathogenesis of cardiovascular disease [22–24] — appeared to be most upregulated (more than 10-fold). CCL14 was the only chemokine tested in the protein array for which a reduction was seen after infection in HUVEC, but not in HSaVEC. CCL14 was also down-regulated 2.31 fold (ANOVA p-value 5.27E-14) in the transcriptional profiling analysis after infection of HUVEC. Interestingly, CCL14 is highly expressed during early pregnancy by the human endometrium [25] and has been implicated in regulating the implantation of the embryo at the fetal-maternal interface. The corresponding receptor of CCL14, CCR1, which is mainly expressed by trophoblasts, was also down-regulated 5.70-fold (ANOVA p-value 6.96E-03) as detected by microarray analysis in HUVEC after RV infection. Thus, perturbation of this chemokine-receptor network might interfere with maternal-fetal communication and contribute to RV-associated embryo resorption or disturbed placental and fetal development. Interestingly, this receptor was also found to be down-regulated following infection of monocytes with another teratogen, HCMV [26]. However, the role of RV infection in vivo on CCL14 and CCR1 expression requires further investigation.

The most prominent clinical manifestations observed in CRS patients are impairment in vision and hearing as well as cardiovascular abnormalities. As of today, it is not understood which mechanisms contribute to this symptomatology or which genes are involved in the RV-induced pathophysiology. However, dysfunction of the sensory organs has been speculated to be a result of vascular insufficiency causing nutrient deprivation rather than being caused by direct viral damage [3]. By examining differences in gene expression between endothelial cells of fetal and adult origin, we wanted to investigate which genes might contribute to the RV-induced pathophysiology in the fetus. GO term-based cluster analysis of the down-regulated genes revealed that terms belonging to sensory organ development (GO term “sensory organ development”, “ear development”, “eye development”) were only enriched for HUVEC. Gene expression analysis by qPCR of RV-infected HUVEC also showed a significant down-regulation of eight genes belonging to this GO term.

While all genes encode proteins that possess functions in sensory organ development, their molecular functions and associated diseases are diverse. The genes ceroid-lipofuscinosis, neuronal 8 (*CLN8*) and *NHS* play a role during sensory organ development of the eye: *CLN8* has a function in lipid synthesis and transport and mutations are associated with progressive epilepsy, mental retardation, and vison impairment [27]. Mutations in the *NHS* gene are associated with Nance-Horan-Syndrome, characterized by ocular abnormalities such as cataracts. It was demonstrated that the NHS protein is a regulator of actin remodeling, and it was speculated that its function is required for maintaining cell morphology during embryogenesis [28].

The genes *FZD3*, *JAG2*, *NOG* and *SLC25A27*, encode for proteins that are attributed to ear development. The function of FZD3 protein is still under debate, but recent data indicate that FZD3 is involved in regulating axonal navigation during embryonic development [29]. The putative Notch-ligand, JAG2, was shown to control the number of sensory hair cells that form the organ of corti in the cochlea of the inner ear [30, 31]. The gene product of *NOG* is required for neural tube growth and patterning, but was also demonstrated to interfere with bone morphogenetic protein (BMP) signaling, thereby blocking sensory organ morphogenesis of the inner ear in different animal models [32–34]. The function of the mitochondria uncoupling protein (UCP4), encoded by *SLC25A27*, is still a matter of debate; however, expression of this protein has been found in neurosensory cells of the inner ear supporting the hypothesis that UCP4 plays a role in functional maturation of the inner ear [35].

The gene product of *FGFR2* and *MYO7A* are suspected to function both in ear and eye development. Mutations in the *FGFR2* gene have been associated with skeletal abnormalities [36] and Lacrimo-auriculo-dento-digital (LADD [MIM 149730]) syndrome, a disorder characterized by hearing loss, deformity in the network of structures of the eye, lacrimal-duct aplasia, and malformations of teeth, forearms, and fingers. Defects in *MYO7A* have been shown to cause Usher syndrome, a disorder characterized by sensorineural hearing loss combined with retinitis pigmentosa [37, 38].

*FZD3*, *NOG* and *SLC25A27* were also shown to be down-regulated in HSaVEC, indicating that the regulation of these genes seems to be independent of cell origin. Nevertheless, one could imagine that interference with the expression of these three genes during embryonic development would have a profound effect on the organism, whereas the effect would not be as dramatic in the adult. In contrast, *ADAMTS18*, *CLN8*, *RDH10* and *TNPO1* were shown to be up-regulated following infection in HSaVEC, indicating that gene regulation differs for these genes between the endothelial cells.

Development of organs belonging to the sensory system is complex and must be precisely coordinated and controlled by intrinsic and extrinsic factors. Interference with the expression of genes involved in these processes may be fatal during embryogenesis and, furthermore, the down-regulation of these genes may give some explanations as to why RV infection is often associated with vision and hearing loss, but causes only mild and transient symptoms in patients infected postnatally. Interestingly, in a study by M. P. Adamo, et al. [14], the here detected down-regulated genes belonging to the GO term “sensory organ development” were not shown to be differentially regulated in their transcriptome analysis of the RV-infected human embryonic fibroblasts (HEF).

In order to the see common and different patterns in gene expression between our primary cells and the cell lines used in the transcriptome analysis carried out by M. P. Adamo, et al. [14], the gene lists of the cells of fetal and adult origin from both studies were compared with each other (Additional file 4 (a)). In the study of M. P. Adamo, et al. [14] gene expression of primary human embryo fibroblasts (HEF) was compared with a fibroblast cell line from the human adult lung (Hs888Lu) following RV infection. Interestingly, when comparing the up-and down-regulated genes between the HUVEC, HSaVEC, Hs888Lu, and HEF, only a small overlap was detected. 86 genes were commonly up-regulated in HUVEC and HEF, whereas only 56 genes were commonly up-regulated in HSaVEC and Hs888Lu. When comparing the down-regulated genes, the overlap was even smaller with eight and three commonly down-regulated genes for fetal and adult cells, respectively. Not surprisingly, the commonly up-regulated genes of fetal cells showed an enrichment of GO-terms involved in viral defense mechanisms (Additional file 4 (b)). For the other commonly regulated genes, no GO-terms were enriched. However, most of the up-regulated genes of adult cells also contained pro-inflammatory genes involved in viral defense mechanisms. Many antiviral strategies are switched on upon viral infection and are conserved, even if different viruses and cell types are analyzed. Thus, it is not surprising that antiviral genes were up-regulated in the endothelial cells as well as HEF and Hs888Lu. However, a down-regulation of genes belonging to the GO term “sensory organ development” was only observed in our study in HUVEC. Our use of a different virus strain and a later harvest time, but more importantly the use of a different cell type, might be responsible for this observation. Since damage to the vascular endothelium has been observed in RV-infected fetuses, we believe that our primary endothelial cell model is well suited for studying RV teratogenesis and we speculate that the prominent clinical manifestations observed in CRS patients arise due to the deregulation of sensory organ development genes in endothelial cells.

## Conclusion

The data presented here show that RV infection perturbs the gene expression of its host. While most RV-induced changes in transcription are common to endothelial cells of fetal and adult origin (including those involved in antiviral processes), we also detected unique gene expression changes depending on the cell type. Most remarkably, a set of genes involved in sensory organ development was down-regulated in the primary cells of fetal origin. Since we could show that RV efficiently replicates in the primary endothelial cells and since lesions in the endothelium are a prominent finding in histopathological studies of CRS fetuses, we propose that replication of RV in the fetal endothelium leads to a down-regulation of genes required for ear and eye development. It still remains unknown, however, which viral gene products and cellular transcription factors are responsible for the down-regulation of these genes. Nevertheless, the down-regulation was mainly observed in the primary endothelial cells of fetal origin. Thus, we believe that the special cellular environment during fetal and embryonic development plays a major role in this process and further studies should be conducted with primary fetal endothelial cells, such as HUVEC, rather than non-fetal cell lines.

## Methods

### Cell culture and viruses

Two pools of HUVEC (from three donors, Promocell) and HSaVEC from two single donors, Promocell), between passage three and eight, were cultured in Endothelial Cell Growth Medium 2 (Promocell), supplemented with 100 μg/ml streptomycin and 100 U/ml penicillin in a humidified atmosphere with 5 % CO_2_ at 37 °C. The clinical isolate RVi/Wuerzburg.DEU/47.11 (Wb-12, genotype 2B) was provided by Dr. Benedikt Weissbrich at the University of Wuerzburg, propagated on Vero76 cells (ATCC) and titered by the immunocolorimetric plaque assay [39].

For growth curve analysis, cells were seeded in 48-well culture plates at a density of 5°10^4^ cells per well and infected with Wb-12 at an MOI of 5 the following day. After adsorption for 2 h at 35 °C, the viral inoculum was removed, the cells were washed three times with 1x PBS and overlaid with 500 μl fresh medium. Supernatants and cells were collected at the indicated time points. The amount of intracellular virus was determined by dissolving the cell pellet in 500 μl fresh medium followed by three subsequent freeze-thaw cycles and centrifugation for 5 min at 400∙g to remove debris. Virus titer of the supernatant was determined by immunocolorimetric plaque assay on Vero76 cells in duplicate.

### Microarray, hybridization and gene expression analysis

For microarray analysis, 2°10^6^ endothelial cells of fetal and adult origin were infected with RV at an MOI of 10 and cells were lysed 36 h post infection with 600 μl RLT buffer from the RNAeasy kit (Qiagen). RNA samples from three independently RV-infected or mock-infected cell cultures were used for each analysis. RNA-extraction, microarray chip hybridization and processing were performed by ATLAS Biolabs GmbH. DNA microarray analysis was carried out using the Affymetrix Human Genome U133 Plus 2.0 array (Affymetrix). The obtained data were further processed, checked for quality, and filtered using the Affymetrix Expression Console Software. Gene level analysis was further conducted with Affymetrix Transcriptome Analysis Console 2.0 software.

Analysis of enriched biological processes and Kyoto Encyclopedia of Genes and Genomes (KEGG) pathways of the differentially expressed genes after RV infection was carried out using the Cytoscape v 3.1.1 plugin ClueGO v2.1.1 [40]. Processes and pathways that possessed a p-value of ≤ 0.01 were displayed. Term and group significance were calculated using a two-sided hypergeometric test and a Bonferroni correction of the p-value.

### Quantitative real-time PCR (qPCR)

Total RNA from five independent infections of HUVEC and six independent infections of HSaVEC, was harvested 36 h post infection using the RNAeasy Mini Kit (Qiagen) according to manufacturer’s instructions. In order to account for donor-to-donor variation, experiments with HUVEC were conducted using two different pools of cells (with each pool consisting of cells from three different donors); furthermore, experiments with HSaVEC were carried out on cells from two different donors. Approximately 6 · 10^5^ cells were used per RNA isolation column and remaining DNA contaminants were removed by a 30 min digest with 20 U of Turbo-DNase (Ambion). RNA was eluted twice with RNase-free water and the RNA concentration was determined using the NanoDrop 8000 (Thermo Scientific). For cDNA generation, 1 μg of RNA was incubated for 1 h at 45 °C with the following components: 1 unit RevertAid™ H minus reverse transcriptase, 5 μM oligo(dT)18 primer, 1x reaction buffer, 1 mM dNTP, and 20 U RiboLock RNase inhibitor (Fermentas). The reaction was terminated by heating the mixture for 10 min at 70 °C. Reactions were performed in a total volume of 25 μl, consisting of 1x SYBR® Green PCR Master Mix (Life Technology), forward and reverse primers (200 nM each) and 5 μl of cDNA, dilutions ranging from 1:5 to 1:50. Cycling conditions were as follows: 95 °C for 15 min, followed by 40 cycles of 95 °C for 15 s and 60 °C for 30 s using the LightCycler 480 system (Roche). Fluorescence readings were recorded at the last step. Melting curve analysis was performed after amplification to determine the presence of nonspecific amplification products. All primer pairs used in this study are listed in Additional file 5. Primers for the amplification of *NOG* were purchased from Biorad (Germany). Gene expression, normalized to *GAPDH*, was determined using the ∆∆Ct mathematical model (Livak & Schmittgen, 2001). The paired one-tailed *t*-test was used to determine statistically significant differences in ∆CT values between non-infected and infected groups.

### Protein microarray and flow cytometry analysis

For protein microarray analysis, 2 · 10^5^ HUVEC and HSaVEC were infected with RV at an MOI of ten in two independent experiments. Supernatants were collected 48 hpi and analyzed for the presence of chemokines using the Proteome Profiler Human Chemokine Array Kit (R&D Systems, Minneapolis, MN, USA) according to the manufacturer’s instructions. For flow cytometry analysis, 1°10^5^ HUVEC and HSaVEC were infected with RV at an MOI of 5 and detached with trypsin at various time points post infection. Following fixation with 2 % paraformaldehyde for 20 min, cells were permeabilized with 0.5 % saponine and stained with a RV anti-capsid antibody (Meridian Life Science, USA) and a secondary goat anti-mouse APC (Biolegend, USA). Flow cytometry data were acquired on a FACSCalibur flow cytometer (BD Biosciences, USA) using Cell Quest Pro software (BD Biosciences).

## Additional files

Additional file 1:
**List of transcripts that were differentially regulated after RV infection.** (PDF 241 kb) Additional file 2:
**Absolute chemokine expression following RV infection.** (PDF 761 kb) Additional file 3:
**Ranking of commonly up- and down-regulated genes in the endothelial cells following RV infection.** (PDF 52 kb) Additional file 4:
**Comparison of differentially regulated genes following RV infection in HUVEC, HSaVEC, HEF and Hs888Lu.** (PDF 244 kb) Additional file 5:
**List of primer sequences used for expression analysis of genes belonging to the GO term “sensory organ development” by qPCR.** (PDF 7 kb)

## Abbreviations

*ADAMTS18*: ADAM metallopeptidase with thrombospondin type 1 motif, 18
*ALDH1A2*: aldehyde dehydrogenase 1 family, member A2
*CLN8*: ceroid-lipofuscinosis, neuronal 8
CPE: cytopathic effect
CRS: congenital rubella syndrome
*DSCAM*: down syndrome cell adhesion molecule
*FGFR2*: fibroblast growth factor receptor 2
*FZD3*: frizzled class receptor 3
GO: gene ontology
HSaVEC: human saphenous vein endothelial cells
HUVEC: human umbilical vein endothelial cells
*JAG2*: jagged 2
KEGG: Kyoto Encyclopedia of Genes and Genomes
*MYO3A*: myosin 3A
*MYO7A*: myosin 7A
*NHS*: Nance-Horan syndrome
*NOG*: noggin
*NTRK3*: neurotrophic tyrosine kinase, receptor, type 3
*PDGFRA*: platelet-derived growth factor receptor, alpha polypeptide
*RDH10*: retinol dehydrogenase 10
RV: rubella virus
*SLC25A27*: solute carrier family 25, member 27
*TNPO1*: transportin 1
*TRPM1*: transient receptor potential cation channel, subfamily M, member 1
*TSPAN12*: tetraspanin 12
