# Supplementary material for: Gene expression profiling of rubella virus infected primary endothelial cells of fetal and adult origin
Source: Virol J. 2016 Feb 2;13:21. doi: 10.1186/s12985-016-0475-9 (PMC4736114; doi:10.1186/s12985-016-0475-9)
Supplement: Additional file 1: — List of transcripts that were differentially regulated after RV infection. (PDF 241 kb) [file 12985_2016_475_MOESM1_ESM.pdf]

Additional File 1. List of transcripts that were differentially regulated after RV infection. Displayed are the average fold changes from three independent experiments of RV-infected cells in comparison to non-infected cells detected by the microarray that meet the selected criteria (i.e. fold change cut-off ≤ -4 and ≥ 4 and ANOVA p-value of ≤ 0.01)

Up-regulated genes following RV-infection in HUVEC

Gene Symbol	Description	Fold Change	ANOVA p-value
MX2	myxovirus (influenza virus) resistance 2 (mouse)	5545.10	4.07E-04
CXCL10	chemokine (C-X-C motif) ligand 10	4833.33	5.00E-05
OASL	2'-5'-oligoadenylate synthetase-like	4179.14	2.00E-05
IFNB1	interferon, beta 1, fibroblast	3134.23	2.00E-06
TNFSF13B	tumor necrosis factor (ligand) superfamily, member 13b	1861.94	1.30E-04
TNFAIP6	tumor necrosis factor, alpha-induced protein 6	1708.13	1.08E-04
CCL5	chemokine (C-C motif) ligand 5	1394.87	6.20E-05
CCL5	chemokine (C-C motif) ligand 5	1254.72	2.67E-04
SCN3A	sodium channel, voltage-gated, type III, alpha subunit	1021.88	5.81E-04
C1S	complement component 1, s subcomponent	913.33	9.00E-05
RSAD2	radical S-adenosyl methionine domain containing 2	905.79	3.25E-04
RSAD2	radical S-adenosyl methionine domain containing 2	848.74	9.38E-07
IFIT1	interferon-induced protein with tetratricopeptide repeats 1	752.49	1.20E-05
IDO1	indoleamine 2,3-dioxygenase 1	644.88	3.68E-04
CCL8	chemokine (C-C motif) ligand 8	579.29	5.80E-05
IFIT2	interferon-induced protein with tetratricopeptide repeats 2	574.79	1.75E-04
IL28A	interleukin 28A (interferon, lambda 2)	573.89	9.48E-04
ELOVL7	ELOVL fatty acid elongase 7	554.79	9.10E-07
TNIP3	TNFAIP3 interacting protein 3	514.11	9.00E-06
RTP4	receptor (chemosensory) transporter protein 4	467.24	7.20E-05
IFIT3	interferon-induced protein with tetratricopeptide repeats 3	427.80	9.40E-05
CCL5	chemokine (C-C motif) ligand 5	414.38	4.83E-04
GABBR1 , UBD	gamma-aminobutyric acid (GABA) B receptor, 1 ; ubiquitin D	413.13	7.50E-05
CXCL11	chemokine (C-X-C motif) ligand 11	382.38	3.80E-05
OASL	2'-5'-oligoadenylate synthetase-like	373.68	4.20E-05
IL12RB1	interleukin 12 receptor, beta 1	338.97	2.92E-03
PDZK1IP1	PDZK1 interacting protein 1	283.73	1.48E-04
IFI44L	interferon-induced protein 44-like	261.15	2.53E-07
AIM2	absent in melanoma 2	258.91	8.75E-03
BTC	betacellulin	258.43	1.00E-06
CCL4	chemokine (C-C motif) ligand 4	231.15	3.85E-04
SELE	selectin E	219.64	1.10E-05
CFB	complement factor B	211.67	4.46E-07
SLC15A3	solute carrier family 15, member 3	204.15	2.27E-03
KYNU	kynureninase	203.50	5.41E-03
BIRC3	baculoviral IAP repeat containing 3	202.23	1.80E-05
SECTM1	secreted and transmembrane 1	200.94	2.00E-04

CCL3 , CCL3L1 , CCL3L3	chemokine (C-C motif) ligand 3 ; chemokine (C-C motif) ligand 3-like 1 ; chemokine (C-C motif) ligand 3-like 3	194.09	9.38E-04
STATH	statherin	185.76	5.75E-04
VCAM1	vascular cell adhesion molecule 1	182.08	3.80E-05
ISG20	interferon stimulated exonuclease gene 20kDa	179.66	3.90E-05
CD38	CD38 molecule	175.23	6.05E-04
C3	complement component 3	167.60	1.50E-05
TNFSF13B	tumor necrosis factor (ligand) superfamily, member 13b	163.07	1.38E-03
EPSTI1	epithelial stromal interaction 1 (breast)	162.60	6.63E-07
ADAM28	ADAM metallopeptidase domain 28	159.60	5.25E-03
TNFAIP6	tumor necrosis factor, alpha-induced protein 6	157.27	3.20E-05
CR1 , CR1L	complement component (3b/4b) receptor 1 (Knops blood group) ; complement component (3b/4b) receptor 1-like	156.10	7.86E-09
CSAG2 , CSAG3	CSAG family, member 2 ; CSAG family, member 3	155.46	3.55E-03
MX1	myxovirus (influenza virus) resistance 1, interferon-inducible protein p78 (mouse)	148.14	7.00E-06
ISG20	interferon stimulated exonuclease gene 20kDa	145.77	6.00E-06
TEX14	testis expressed 14	142.62	1.88E-03
CTSS	cathepsin S	139.28	2.54E-04
CMPK2	cytidine monophosphate (UMP-CMP) kinase 2, mitochondrial	137.38	5.00E-06
SIDT1	SID1 transmembrane family, member 1	137.35	4.70E-05
OAS2	2'-5'-oligoadenylate synthetase 2, 69/71kDa	137.25	4.79E-07
BTC	betacellulin	122.28	2.08E-04
ANGPTL1	angiopoietin-like 1	118.62	7.44E-04
CCL7	chemokine (C-C motif) ligand 7	115.92	2.95E-03
IFI30	interferon, gamma-inducible protein 30	114.26	3.40E-05
CCL20	chemokine (C-C motif) ligand 20	112.23	3.58E-07
SERPINA3	serpin peptidase inhibitor, clade A (alpha-1 antiproteinase, antitrypsin), member 3	106.73	4.93E-07
PI3	peptidase inhibitor 3, skin-derived	102.08	2.40E-05
HERC5	HECT and RLD domain containing E3 ubiquitin protein ligase 5	102.00	7.00E-06
LOC100129518 , SOD2	uncharacterized LOC100129518 ; superoxide dismutase 2, mitochondrial	99.88	3.13E-03
GBP4	guanylate binding protein 4	99.70	4.00E-06
BDKRB2	bradykinin receptor B2	97.40	5.10E-03
EPSTI1	epithelial stromal interaction 1 (breast)	96.65	1.75E-07
IFIT3	interferon-induced protein with tetratricopeptide repeats 3	96.25	1.00E-06
CXCL3	chemokine (C-X-C motif) ligand 3	92.97	1.30E-05
PDZK1IP1	PDZK1 interacting protein 1	87.35	2.23E-03
IL7	interleukin 7	85.03	2.84E-04
CXCL6	chemokine (C-X-C motif) ligand 6 (granulocyte chemotactic protein 2)	84.27	2.70E-05
IFIT2	interferon-induced protein with tetratricopeptide repeats 2	83.88	4.00E-06
PTGS2	prostaglandin-endoperoxide synthase 2 (prostaglandin G/H synthase and cyclooxygenase)	79.05	4.44E-04
MAB21L2	mab-21-like 2 (C. elegans)	74.65	2.45E-03
NCF2	neutrophil cytosolic factor 2	74.48	4.01E-03

ANGPTL1	angiopoietin-like 1	70.47	6.50E-05
GBP5	guanylate binding protein 5	69.45	1.54E-03
BATF2	basic leucine zipper transcription factor, ATF-like 2	69.35	7.00E-07
TBC1D1	TBC1 (tre-2/USP6, BUB2, cdc16) domain family, member 1	66.42	1.08E-04
SERPINB2	serpin peptidase inhibitor, clade B (ovalbumin), member 2	65.61	1.56E-03
P2RY6	pyrimidinergic receptor P2Y, G-protein coupled, 6	61.23	2.88E-03
IL29	interleukin 29 (interferon, lambda 1)	60.04	2.54E-03
CSF2	colony stimulating factor 2 (granulocyte-macrophage)	59.87	6.50E-05
IFI35	interferon-induced protein 35	59.24	6.00E-06
TNFRSF9	tumor necrosis factor receptor superfamily, member 9	58.82	1.20E-05
IRG1	immunoresponsive 1 homolog (mouse)	58.25	2.00E-06
ICAM1	intercellular adhesion molecule 1	57.33	3.68E-07
TLR2	toll-like receptor 2	57.15	4.10E-05
GBP5	guanylate binding protein 5	56.15	6.02E-04
HERC6	HECT and RLD domain containing E3 ubiquitin protein ligase family member 6	55.79	3.20E-07
IFIH1	interferon induced with helicase C domain 1	55.65	3.44E-08
S100P	S100 calcium binding protein P	55.19	4.08E-04
CR1L	complement component (3b/4b) receptor 1-like	54.96	1.29E-03
PI3	peptidase inhibitor 3, skin-derived	54.49	1.41E-03
KLRD1	killer cell lectin-like receptor subfamily D, member 1	53.90	1.61E-04
ATP10A	ATPase, class V, type 10A	52.86	4.90E-05
LGALS3BP	lectin, galactoside-binding, soluble, 3 binding protein	52.44	1.20E-05
SAA1 , SAA2	serum amyloid A1 ; serum amyloid A2	52.14	9.61E-03
IFIH1	interferon induced with helicase C domain 1	52.13	9.00E-06
TSLP	thymic stromal lymphopoietin	51.29	3.77E-04
TAC3	tachykinin 3	50.60	2.07E-04
OAS3	2'-5'-oligoadenylate synthetase 3, 100kDa	50.44	3.00E-06
SLCO5A1	solute carrier organic anion transporter family, member 5A1	50.26	8.00E-06
CTSS	cathepsin S	50.21	5.80E-04
CSF3	colony stimulating factor 3 (granulocyte)	49.96	8.00E-06
IFI6	interferon, alpha-inducible protein 6	49.95	3.40E-05
NEURL3	neuralized homolog 3 (Drosophila) pseudogene	49.61	4.40E-05
LOC100505474	uncharacterized LOC100505474	49.14	1.19E-03
TNFAIP3	tumor necrosis factor, alpha-induced protein 3	48.63	1.00E-05
CYP1B1	cytochrome P450, family 1, subfamily B, polypeptide 1	47.92	6.70E-05
HSH2D	hematopoietic SH2 domain containing	47.91	2.56E-03
GBP4	guanylate binding protein 4	47.82	1.93E-03
APOL6	apolipoprotein L, 6	47.63	4.60E-03
KLK10	kallikrein-related peptidase 10	46.43	2.26E-08
CTSS	cathepsin S	46.43	5.00E-06
MAK	male germ cell-associated kinase	45.53	2.94E-03
CXCL5	chemokine (C-X-C motif) ligand 5	44.83	2.77E-04
OAS1	2'-5'-oligoadenylate synthetase 1, 40/46kDa	44.13	3.83E-07
IFITM1	interferon induced transmembrane protein 1	43.13	3.00E-06
APOL6	apolipoprotein L, 6	43.11	3.70E-05

PSMB9	proteasome (prosome, macropain) subunit, beta type, 9 (large multifunctional peptidase 2)	42.81	1.30E-05
SLC1A3	solute carrier family 1 (glial high affinity glutamate transporter), member 3	42.79	7.19E-03
CXCL9	chemokine (C-X-C motif) ligand 9	41.56	1.80E-04
CXCL11	chemokine (C-X-C motif) ligand 11	41.42	1.10E-05
ETV7	ets variant 7	41.29	1.29E-03
CLDN23	claudin 23	41.16	1.45E-03
PIK3AP1	phosphoinositide-3-kinase adaptor protein 1	40.71	1.30E-03
BCL2A1	BCL2-related protein A1	40.04	1.00E-05
RET	ret proto-oncogene	40.04	5.19E-03
ODF3B	outer dense fiber of sperm tails 3B	39.54	9.88E-03
LRP2	low density lipoprotein receptor-related protein 2	39.39	9.83E-03
CH25H	cholesterol 25-hydroxylase	39.06	1.55E-03
RARRES3	retinoic acid receptor responder (tazarotene induced) 3	38.70	3.75E-07
APOBEC3G	apolipoprotein B mRNA editing enzyme, catalytic polypeptide-like 3G	38.34	4.80E-05
KLRD1	killer cell lectin-like receptor subfamily D, member 1	38.27	2.40E-05
KYNU	kynureninase	37.64	2.74E-04
CLEC7A	C-type lectin domain family 7, member A	37.07	1.30E-05
CASP1	caspase 1, apoptosis-related cysteine peptidase	36.96	2.51E-07
TMEM229B	transmembrane protein 229B	36.62	5.38E-04
CASP1	caspase 1, apoptosis-related cysteine peptidase	36.17	6.00E-06
LRRN3	leucine rich repeat neuronal 3	35.82	7.45E-03
CXCL12	chemokine (C-X-C motif) ligand 12	35.66	4.72E-03
TNFAIP3	tumor necrosis factor, alpha-induced protein 3	35.56	7.65E-08
TLR3	toll-like receptor 3	35.52	3.00E-06
ANGPTL1	angiopoietin-like 1	35.33	2.38E-04
CLDN23	Claudin 23	35.06	5.14E-03
OAS1	2'-5'-oligoadenylate synthetase 1, 40/46kDa	34.15	1.50E-05
LOC100129518 , SOD2	uncharacterized LOC100129518 ; superoxide dismutase 2, mitochondrial	33.46	3.20E-07
C1R	complement component 1, r subcomponent	33.44	3.00E-06
ICAM1	intercellular adhesion molecule 1	33.30	7.38E-07
CASP1	caspase 1, apoptosis-related cysteine peptidase	33.29	1.30E-05
IL1B	interleukin 1, beta	33.22	1.10E-05
CD69	CD69 molecule	32.53	9.00E-06
C1S	complement component 1, s subcomponent	31.61	3.97E-04
SPAG6	sperm associated antigen 6	31.58	2.60E-05
FCGR2A	Fc fragment of IgG, low affinity IIa, receptor (CD32)	31.49	8.20E-05
ETV7	ets variant 7	30.10	1.60E-04
IL1RN	interleukin 1 receptor antagonist	29.65	4.59E-04
APOL6	apolipoprotein L, 6	29.63	1.48E-04
USP30-AS1	USP30 antisense RNA 1 (non-protein coding)	29.39	9.53E-03
SEMA3A	sema domain, immunoglobulin domain (Ig), short basic domain, secreted, (semaphorin) 3A	28.70	8.79E-04
CXCL2	chemokine (C-X-C motif) ligand 2	28.56	3.00E-06
DENND2D	DENN/MADD domain containing 2D	28.51	2.60E-03
DSP	desmoplakin	28.43	8.09E-03
LOC100129518 ,	uncharacterized LOC100129518 ; superoxide	28.06	1.27E-07

SOD2	dismutase 2, mitochondrial		
IL18RAP	interleukin 18 receptor accessory protein	27.86	7.01E-03
SAMD9	sterile alpha motif domain containing 9	27.62	1.02E-04
DDX58	DEAD (Asp-Glu-Ala-Asp) box polypeptide 58	27.53	1.00E-06
HCG4	HLA complex group 4 (non-protein coding)	27.44	5.08E-04
CHI3L1	chitinase 3-like 1 (cartilage glycoprotein-39)	27.18	1.04E-03
OAS2	2'-5'-oligoadenylate synthetase 2, 69/71kDa	26.88	1.60E-03
TNC	tenascin C	26.64	2.50E-04
HOXD10	homeobox D10	26.16	1.30E-04
LOC100287705 , PTN	uncharacterized LOC100287705 ; pleiotrophin	25.82	8.20E-05
TBC1D1	TBC1 (tre-2/USP6, BUB2, cdc16) domain family, member 1	25.76	1.81E-03
IL8	interleukin 8	25.72	1.07E-04
CASP1	caspase 1, apoptosis-related cysteine peptidase	25.64	1.20E-07
EGR3	early growth response 3	25.62	3.01E-04
SLC22A16	solute carrier family 22 (organic cation/carnitine transporter), member 16	25.42	5.63E-04
SGPP2	sphingosine-1-phosphate phosphatase 2	25.19	6.90E-05
RHBDL2	rhomboid, veinlet-like 2 (Drosophila)	24.57	7.20E-05
SAMD9L	sterile alpha motif domain containing 9-like	24.47	3.60E-05
IFIH1	Interferon induced with helicase C domain 1	24.42	1.80E-04
HLA-F	major histocompatibility complex, class I, F	24.35	1.21E-04
C6orf58	chromosome 6 open reading frame 58	23.95	2.31E-03
BIRC3	baculoviral IAP repeat containing 3	23.61	5.64E-03
CX3CL1	chemokine (C-X3-C motif) ligand 1	23.37	5.49E-07
ANKRD45	ankyrin repeat domain 45	23.17	1.26E-03
CLDN23	claudin 23	23.13	5.00E-06
GBP1	guanylate binding protein 1, interferon-inducible	23.09	1.67E-04
CXCL5	chemokine (C-X-C motif) ligand 5	22.63	5.21E-04
APOL1	apolipoprotein L, 1	22.28	1.10E-05
GBP1	guanylate binding protein 1, interferon-inducible	22.05	5.00E-06
ICAM1	intercellular adhesion molecule 1	21.57	5.43E-07
PPM1K	protein phosphatase, Mg2+/Mn2+ dependent, 1K	21.42	1.30E-05
RNF213	ring finger protein 213	21.22	5.10E-05
CCL2	chemokine (C-C motif) ligand 2	21.03	6.60E-05
LOC100129518 , SOD2	uncharacterized LOC100129518 ; superoxide dismutase 2, mitochondrial	20.78	1.70E-05
CASP1	caspase 1, apoptosis-related cysteine peptidase	20.70	1.00E-06
LOC100506373	uncharacterized LOC100506373	20.58	2.73E-03
C2	complement component 2	20.56	1.41E-03
RNF175	ring finger protein 175	20.51	6.31E-04
IFITM1 , IFITM2	interferon induced transmembrane protein 1 ; interferon induced transmembrane protein 2	20.20	4.00E-06
HCP5	HLA complex P5 (non-protein coding)	20.09	2.68E-04
ODF3B	outer dense fiber of sperm tails 3B	20.01	2.59E-03
FAM65C	family with sequence similarity 65, member C	19.95	1.80E-04
APOL6	apolipoprotein L, 6	19.95	2.00E-06
ISG15	ISG15 ubiquitin-like modifier	19.84	1.10E-05
CD83	CD83 molecule	19.65	4.31E-07

SSTR2	somatostatin receptor 2	19.64	1.96E-03
ADAM28	ADAM metallopeptidase domain 28	19.62	1.33E-04
LOC284561	uncharacterized LOC284561	19.46	1.00E-03
PARP10	Poly (ADP-ribose) polymerase family, member 10	19.34	1.84E-04
DDX58	DEAD (Asp-Glu-Ala-Asp) box polypeptide 58	19.06	2.00E-06
RASGRF1	Ras protein-specific guanine nucleotide-releasing factor 1	19.03	5.17E-03
SAMHD1	SAM domain and HD domain 1	19.01	1.19E-04
PLEKHA4	pleckstrin homology domain containing, family A (phosphoinositide binding specific) member 4	18.48	4.01E-04
LPPR4	lipid phosphate phosphatase-related protein type 4	18.40	2.98E-04
OAS2	2'-5'-oligoadenylate synthetase 2, 69/71kDa	18.35	9.40E-05
MUC4	mucin 4, cell surface associated	18.27	3.25E-04
SPARCL1	SPARC-like 1 (hevin)	18.26	3.24E-04
GXYLT2	glucoside xylosyltransferase 2	18.13	8.79E-03
F3	coagulation factor III (thromboplastin, tissue factor)	18.00	5.00E-06
XAF1	XIAP associated factor 1	17.94	8.00E-06
NKAIN1	Na+/K+ transporting ATPase interacting 1	17.81	1.02E-03
NKX3-1	NK3 homeobox 1	17.56	1.40E-05
BANCR	BRAF-activated non-protein coding RNA	17.23	1.46E-03
KLB	klotho beta	17.19	5.98E-03
IL18BP	interleukin 18 binding protein	17.12	3.00E-06
C8orf34	chromosome 8 open reading frame 34	17.01	9.20E-05
SAA1 , SAA2 , SAA2-SAA4	serum amyloid A1 ; serum amyloid A2 ; SAA2-SAA4 readthrough	16.83	1.20E-03
GPR84	G protein-coupled receptor 84	16.75	2.00E-06
PRIC285	peroxisomal proliferator-activated receptor A interacting complex 285	16.73	6.50E-05
SP110	SP110 nuclear body protein	16.70	6.90E-05
HERC6	HECT and RLD domain containing E3 ubiquitin protein ligase family member 6	16.70	3.81E-04
IL6	interleukin 6 (interferon, beta 2)	16.68	1.40E-05
XAF1	XIAP associated factor 1	16.56	2.40E-05
IFI44	Interferon-induced protein 44	16.51	1.90E-05
HSD11B1	hydroxysteroid (11-beta) dehydrogenase 1	16.46	5.81E-03
CEBPD	CCAAT/enhancer binding protein (C/EBP), delta	16.35	3.96E-04
HPSE	heparanase	16.28	1.67E-03
GBP1	guanylate binding protein 1, interferon-inducible	16.21	2.20E-05
HLA-F	major histocompatibility complex, class I, F	16.19	4.00E-06
KLRD1	killer cell lectin-like receptor subfamily D, member 1	16.17	3.12E-04
SAMD9L	sterile alpha motif domain containing 9-like	15.84	1.00E-06
KIAA0146	KIAA0146	15.73	2.70E-05
LY6E	lymphocyte antigen 6 complex, locus E	15.70	3.60E-05
CYP2J2	cytochrome P450, family 2, subfamily J, polypeptide 2	15.64	4.03E-04
ARHGAP15	Rho GTPase activating protein 15	15.48	2.26E-03
AKIP1 , NUAK2	A kinase (PRKA) interacting protein 1 ; NUAK family, SNF1-like kinase, 2	15.44	3.60E-05
PLA1A	phospholipase A1 member A	15.33	2.70E-05
IGFBP5	insulin-like growth factor binding protein 5	15.25	1.93E-03
CHI3L2	chitinase 3-like 2	15.24	3.92E-03

NR4A3	nuclear receptor subfamily 4, group A, member 3	15.01	7.91E-04
ZC3H12A	zinc finger CCCH-type containing 12A	14.98	2.60E-05
IFNA10	interferon, alpha 10	14.82	5.29E-04
SP110	SP110 nuclear body protein	14.74	2.10E-05
PDZD2	PDZ domain containing 2	14.73	2.60E-05
HLA-G	major histocompatibility complex, class I, G	14.73	6.06E-04
LIPA , LOC100507575	lipase A, lysosomal acid, cholesterol esterase ; uncharacterized LOC100507575	14.67	2.84E-03
LOC644135	Uncharacterized LOC644135	14.58	2.02E-03
HPSE	heparanase	14.45	8.70E-05
SH2D1B	SH2 domain containing 1B	14.45	1.45E-04
PTGS2	prostaglandin-endoperoxide synthase 2 (prostaglandin G/H synthase and cyclooxygenase)	14.39	7.25E-07
ATF3	activating transcription factor 3	14.32	1.29E-04
SAMHD1	SAM domain and HD domain 1	14.31	1.77E-04
KBTBD12	kelch repeat and BTB (POZ) domain containing 12	14.28	5.39E-04
LCP2	lymphocyte cytosolic protein 2 (SH2 domain containing leukocyte protein of 76kDa)	14.12	1.76E-04
FAM65B	family with sequence similarity 65, member B	14.09	8.56E-03
FAM46A	family with sequence similarity 46, member A	14.06	1.65E-04
TRIM14	tripartite motif containing 14	14.05	2.00E-06
C2CD4A	C2 calcium-dependent domain containing 4A	13.93	7.87E-04
TNFSF10	tumor necrosis factor (ligand) superfamily, member 10	13.66	6.31E-04
MXD1	MAX dimerization protein 1	13.59	5.61E-03
DDX60	DEAD (Asp-Glu-Ala-Asp) box polypeptide 60	13.51	4.90E-05
CHI3L1	chitinase 3-like 1 (cartilage glycoprotein-39)	13.34	5.06E-03
HLA-C	major histocompatibility complex, class I, C	13.34	2.00E-06
MRGPRX3	MAS-related GPR, member X3	13.30	1.87E-03
LOC100505880	uncharacterized LOC100505880	13.19	1.17E-03
FAM46A	family with sequence similarity 46, member A	13.12	1.70E-05
C6orf58	Chromosome 6 open reading frame 58	13.10	4.16E-04
TNFSF10	tumor necrosis factor (ligand) superfamily, member 10	13.08	4.00E-06
STAT1	signal transducer and activator of transcription 1, 91kDa	13.03	2.00E-06
TAP1	transporter 1, ATP-binding cassette, sub-family B (MDR/TAP)	13.02	2.10E-05
HIST2H2AA3 , HIST2H2AA4	histone cluster 2, H2aa3 ; histone cluster 2, H2aa4	13.01	5.00E-06
NTNG2	netrin G2	12.84	2.52E-03
MT1M	metallothionein 1M	12.66	8.80E-07
HIST2H2AA3 , HIST2H2AA4	histone cluster 2, H2aa3 ; histone cluster 2, H2aa4	12.65	2.60E-05
C2	complement component 2	12.64	2.84E-03
TRAF1	TNF receptor-associated factor 1	12.64	3.78E-03
FAM46A	Family with sequence similarity 46, member A	12.57	3.47E-04
STAT1 , STAT1	signal transducer and activator of transcription 1, 91kDa ; signal transducer and activator of transcription 1, 91kDa	12.56	3.22E-07
MAP3K8	mitogen-activated protein kinase kinase kinase 8	12.49	1.64E-03
PMAIP1	phorbol-12-myristate-13-acetate-induced protein 1	12.47	8.00E-06
UNC93B1	unc-93 homolog B1 (C. elegans)	12.44	4.10E-03

CX3CL1	chemokine (C-X3-C motif) ligand 1	12.43	4.66E-03
IL1A	interleukin 1, alpha	12.38	3.33E-04
KIAA0146	KIAA0146	12.31	9.00E-06
IFI44	interferon-induced protein 44	12.14	7.53E-07
ZC3HAV1	zinc finger CCCH-type, antiviral 1	12.13	2.00E-06
BST2	bone marrow stromal cell antigen 2	12.09	1.90E-05
USP18	ubiquitin specific peptidase 18	12.09	3.43E-07
RET	ret proto-oncogene	11.89	1.18E-03
SLAMF8	SLAM family member 8	11.88	1.43E-03
CEACAM1	carcinoembryonic antigen-related cell adhesion molecule 1 (biliary glycoprotein)	11.86	3.30E-05
PARP15	poly (ADP-ribose) polymerase family, member 15	11.78	6.54E-03
ACSL5	acyl-CoA synthetase long-chain family member 5	11.70	6.50E-05
ZNF385D	zinc finger protein 385D	11.61	4.16E-03
GZMA	granzyme A (granzyme 1, cytotoxic T-lymphocyte-associated serine esterase 3)	11.53	2.88E-03
RASGEF1B	RasGEF domain family, member 1B	11.48	1.01E-03
COL1A1	collagen, type I, alpha 1	11.47	1.25E-03
SAMD9L	sterile alpha motif domain containing 9-like	11.34	2.00E-06
WASL	Wiskott-Aldrich syndrome-like	11.33	5.94E-03
HIST2H2BE	histone cluster 2, H2be	11.31	6.00E-06
NRAP	nebulin-related anchoring protein	11.30	1.12E-03
ZNF837	zinc finger protein 837	11.24	1.15E-03
L1CAM	L1 cell adhesion molecule	11.18	8.80E-04
SAMD9	sterile alpha motif domain containing 9	11.14	1.32E-07
GUCY2F	guanylate cyclase 2F, retinal	11.09	3.09E-03
LGALS9	lectin, galactoside-binding, soluble, 9	11.08	7.00E-05
STAT1 , STAT1	signal transducer and activator of transcription 1, 91kDa ; signal transducer and activator of transcription 1, 91kDa	11.06	2.03E-03
PLSCR1	phospholipid scramblase 1	10.98	8.00E-06
RGS1	regulator of G-protein signaling 1	10.91	1.80E-03
IRF1	interferon regulatory factor 1	10.89	1.20E-05
HAR1A	highly accelerated region 1A (non-protein coding)	10.84	6.00E-03
TRIM14	tripartite motif containing 14	10.80	5.30E-05
NFKBIZ	nuclear factor of kappa light polypeptide gene enhancer in B-cells inhibitor, zeta	10.79	1.00E-05
CLEC4E	C-type lectin domain family 4, member E	10.70	4.06E-03
RASGRF1	Ras protein-specific guanine nucleotide-releasing factor 1	10.69	1.60E-03
LAMP3	lysosomal-associated membrane protein 3	10.68	6.12E-07
TNFSF10	tumor necrosis factor (ligand) superfamily, member 10	10.65	2.70E-05
HLA-G	major histocompatibility complex, class I, G	10.63	2.00E-05
TMEM139	transmembrane protein 139	10.61	1.80E-05
EDNRA	endothelin receptor type A	10.57	5.35E-04
HIST2H2AA3	histone cluster 2, H2aa3	10.45	6.00E-06
LOC285628 , MIR146A	uncharacterized LOC285628 ; microRNA 146a	10.45	4.68E-04
FXYD6	FXYD domain containing ion transport regulator 6	10.43	6.50E-05
PRRG4	proline rich Gla (G-carboxyglutamic acid) 4 (transmembrane)	10.36	1.94E-04

IFNA1	interferon, alpha 1	10.30	4.95E-04
C15orf48	chromosome 15 open reading frame 48	10.27	2.30E-03
TNFAIP2	tumor necrosis factor, alpha-induced protein 2	10.23	7.00E-06
VNN3	vanin 3	10.11	2.28E-03
DHX36	DEAH (Asp-Glu-Ala-His) box polypeptide 36	9.96	9.50E-05
CD47	CD47 molecule	9.94	1.34E-03
SP110	SP110 nuclear body protein	9.88	5.19E-07
LOC285556	uncharacterized LOC285556	9.88	1.26E-03
PDE6H	phosphodiesterase 6H, cGMP-specific, cone, gamma	9.87	6.63E-03
RPPH1	ribonuclease P RNA component H1	9.84	5.24E-03
SLC7A2	solute carrier family 7 (cationic amino acid transporter, y+ system), member 2	9.74	4.61E-04
SIK1	salt-inducible kinase 1	9.74	8.70E-05
SEPT4	septin 4	9.73	6.33E-04
VNN3	vanin 3	9.69	3.43E-04
GMPR	guanosine monophosphate reductase	9.65	6.00E-06
IRF7	interferon regulatory factor 7	9.63	1.45E-04
IL3RA	interleukin 3 receptor, alpha (low affinity)	9.59	6.51E-03
IL12A	interleukin 12A (natural killer cell stimulatory factor 1, cytotoxic lymphocyte maturation factor 1, p35)	9.59	7.64E-04
UBE2L6	ubiquitin-conjugating enzyme E2L 6	9.56	9.00E-06
PAPPA	pregnancy-associated plasma protein A, pappalysin 1	9.54	5.21E-04
SPATA6L	spermatogenesis associated 6-like	9.54	8.50E-05
RHCG	Rh family, C glycoprotein	9.51	8.56E-04
NLRC5	NLR family, CARD domain containing 5	9.51	5.30E-05
DTWD1	DTW domain containing 1	9.51	1.46E-03
ODF3B	outer dense fiber of sperm tails 3B	9.50	1.34E-03
LOC100271840	uncharacterized LOC100271840	9.47	3.18E-03
ARAP2	ArfGAP with RhoGAP domain, ankyrin repeat and PH domain 2	9.42	5.58E-04
CTNND2	catenin (cadherin-associated protein), delta 2 (neural plakophilin-related arm-repeat protein)	9.39	4.73E-03
DHX58	DEXH (Asp-Glu-X-His) box polypeptide 58	9.39	1.62E-04
ACSL5	acyl-CoA synthetase long-chain family member 5	9.38	6.30E-05
PF4	platelet factor 4	9.28	6.30E-05
HLA-J	major histocompatibility complex, class I, J (pseudogene)	9.27	5.40E-05
FBXO6	F-box protein 6	9.17	1.50E-04
SAMHD1	SAM domain and HD domain 1	9.17	5.73E-03
APOF	apolipoprotein F	9.13	2.53E-03
WDR96	WD repeat domain 96	9.12	1.45E-03
LIMD1	LIM domains containing 1	9.12	2.52E-03
RIT2	Ras-like without CAAX 2	9.11	1.47E-03
KCTD14 , NDUFC2-KCTD14	potassium channel tetramerisation domain containing 14 ; NDUFC2-KCTD14 readthrough	9.08	7.40E-05
KCTD14 , NDUFC2-KCTD14	potassium channel tetramerisation domain containing 14 ; NDUFC2-KCTD14 readthrough	9.07	1.90E-05
LAX1	lymphocyte transmembrane adaptor 1	8.99	8.60E-04
MSX1	msh homeobox 1	8.98	2.50E-05

NLRP3	NLR family, pyrin domain containing 3	8.98	7.27E-04
ACTN2	actinin, alpha 2	8.96	3.10E-05
PKIB	protein kinase (cAMP-dependent, catalytic) inhibitor beta	8.96	2.63E-04
TNF	tumor necrosis factor	8.93	2.84E-03
CCDC164	coiled-coil domain containing 164	8.93	6.84E-03
NRK	Nik related kinase	8.87	2.80E-03
SLC16A4	solute carrier family 16, member 4 (monocarboxylic acid transporter 5)	8.75	1.09E-04
OAS3	2'-5'-oligoadenylate synthetase 3, 100kDa	8.75	3.51E-03
HLA-B	major histocompatibility complex, class I, B	8.73	1.90E-05
GCH1	GTP cyclohydrolase 1	8.70	1.10E-05
C9orf57	chromosome 9 open reading frame 57	8.70	1.01E-03
CXCL16	chemokine (C-X-C motif) ligand 16	8.68	1.00E-06
SOCS1	suppressor of cytokine signaling 1	8.61	9.61E-04
DDX60L	DEAD (Asp-Glu-Ala-Asp) box polypeptide 60-like	8.61	8.00E-06
NFKBIZ	nuclear factor of kappa light polypeptide gene enhancer in B-cells inhibitor, zeta	8.59	1.14E-03
FST	follistatin	8.56	5.30E-05
SHOX2	short stature homeobox 2	8.56	8.90E-05
PAPPA	pregnancy-associated plasma protein A, pappalysin 1	8.49	1.10E-05
CASZ1	castor zinc finger 1	8.48	3.20E-04
NKAIN2	Na+/K+ transporting ATPase interacting 2	8.44	4.37E-04
SV2B	synaptic vesicle glycoprotein 2B	8.43	7.85E-03
IL8	interleukin 8	8.41	1.00E-06
CSF1	colony stimulating factor 1 (macrophage)	8.41	1.54E-04
BATF3	basic leucine zipper transcription factor, ATF-like 3	8.40	1.32E-04
SLAIN2	SLAIN motif family, member 2	8.39	8.86E-03
SP110	SP110 nuclear body protein	8.36	2.30E-05
PARP9	poly (ADP-ribose) polymerase family, member 9	8.32	9.60E-05
APOBEC3F , APOBEC3G	apolipoprotein B mRNA editing enzyme, catalytic polypeptide-like 3F ; apolipoprotein B mRNA editing enzyme, catalytic polypeptide-like 3G	8.25	5.03E-04
FAM26F	family with sequence similarity 26, member F	8.25	1.77E-04
GPR18	G protein-coupled receptor 18	8.24	1.06E-03
HLA-G	major histocompatibility complex, class I, G	8.22	7.30E-05
ITIH4 , MUSTN1	inter-alpha-trypsin inhibitor heavy chain family, member 4 ; musculoskeletal, embryonic nuclear protein 1	8.21	4.87E-03
FST	follistatin	8.21	7.80E-05
LIF	leukemia inhibitory factor	8.20	3.47E-03
SLC1A2	solute carrier family 1 (glial high affinity glutamate transporter), member 2	8.20	1.51E-03
PARP14	poly (ADP-ribose) polymerase family, member 14	8.20	2.50E-05
RNF213	ring finger protein 213	8.19	4.00E-06
MSX1	Msh homeobox 1	8.17	1.50E-04
JAK3	Janus kinase 3	8.16	1.60E-05
TFAP2A	transcription factor AP-2 alpha (activating enhancer binding protein 2 alpha)	8.14	5.80E-04
ARPC4-TTLL3 , TTLL3	ARPC4-TTLL3 readthrough ; tubulin tyrosine ligase-like family, member 3	8.12	4.55E-03
SAMHD1	SAM domain and HD domain 1	8.11	8.00E-06

HDAC9	histone deacetylase 9	8.07	2.70E-03
EFHD1	EF-hand domain family, member D1	8.06	6.96E-03
BRE-AS1	BRE antisense RNA 1 (non-protein coding)	8.05	1.08E-03
PLA2G1B	phospholipase A2, group IB (pancreas)	8.02	1.74E-04
STAT1 , STAT1	signal transducer and activator of transcription 1, 91kDa ; signal transducer and activator of transcription 1, 91kDa	8.01	5.00E-06
PAPPA	pregnancy-associated plasma protein A, pappalysin 1	8.01	5.00E-06
VNN1	vanin 1	7.98	3.00E-03
OLFML3	olfactomedin-like 3	7.96	2.90E-05
CLDN14	claudin 14	7.95	3.00E-06
ALDOB	aldolase B, fructose-bisphosphate	7.90	7.99E-03
MOCOS	molybdenum cofactor sulfurase	7.84	1.76E-04
RHEBL1	Ras homolog enriched in brain like 1	7.84	3.69E-04
NFKBIA	nuclear factor of kappa light polypeptide gene enhancer in B-cells inhibitor, alpha	7.77	7.90E-05
HLA-B	major histocompatibility complex, class I, B	7.75	3.90E-05
SP110	SP110 nuclear body protein	7.72	2.90E-05
CARD18	caspase recruitment domain family, member 18	7.67	8.37E-04
CLDN1	claudin 1	7.66	4.08E-03
NPAS3	neuronal PAS domain protein 3	7.65	5.70E-05
FRK	fyn-related kinase	7.64	1.58E-03
TFPI2	tissue factor pathway inhibitor 2	7.61	3.59E-04
ACTR6	ARP6 actin-related protein 6 homolog (yeast)	7.61	3.22E-03
C14orf39	chromosome 14 open reading frame 39	7.56	5.76E-03
C5orf47	chromosome 5 open reading frame 47	7.54	7.77E-04
CEACAM1	carcinoembryonic antigen-related cell adhesion molecule 1 (biliary glycoprotein)	7.50	3.56E-04
TAP2	transporter 2, ATP-binding cassette, sub-family B (MDR/TAP)	7.48	2.00E-06
CD47	CD47 molecule	7.48	5.52E-03
FST	follistatin	7.41	2.68E-04
PTPRN2	protein tyrosine phosphatase, receptor type, N polypeptide 2	7.38	6.24E-03
NCOA7	nuclear receptor coactivator 7	7.34	1.00E-06
HLA-B	major histocompatibility complex, class I, B	7.32	1.37E-04
CFHR5	complement factor H-related 5	7.30	1.70E-05
CEACAM1	carcinoembryonic antigen-related cell adhesion molecule 1 (biliary glycoprotein)	7.29	4.62E-04
XRN1	5'-3' exoribonuclease 1	7.28	3.10E-04
XRN1	5'-3' exoribonuclease 1	7.28	1.97E-04
TRIM21	tripartite motif containing 21	7.27	4.20E-05
SLFN5	schlafen family member 5	7.26	6.20E-05
IFIT5	interferon-induced protein with tetratricopeptide repeats 5	7.24	1.90E-05
RNF213	ring finger protein 213	7.24	6.30E-05
TFPI2	tissue factor pathway inhibitor 2	7.21	4.60E-05
DNAAF1	dynein, axonemal, assembly factor 1	7.21	1.95E-03
FGF2	fibroblast growth factor 2 (basic)	7.19	5.10E-05
C12orf39	chromosome 12 open reading frame 39	7.13	3.48E-04
IFIT5	interferon-induced protein with tetratricopeptide repeats 5	7.12	6.00E-06

NPAS3	neuronal PAS domain protein 3	7.12	1.65E-03
CAPN3	calpain 3, (p94)	7.11	1.19E-03
KLF4	Kruppel-like factor 4 (gut)	7.11	1.80E-05
C1orf126	chromosome 1 open reading frame 126	7.10	2.37E-03
RNF152	ring finger protein 152	7.10	3.07E-03
PCDH17	protocadherin 17	7.07	4.68E-04
HRH3	histamine receptor H3	7.06	2.73E-03
EIF1AY	eukaryotic translation initiation factor 1A, Y-linked	7.03	3.31E-03
PLCXD3	phosphatidylinositol-specific phospholipase C, X domain containing 3	7.01	1.99E-03
FAM26F	Family with sequence similarity 26, member F	6.99	6.68E-04
C3AR1	complement component 3a receptor 1	6.97	1.00E-06
WARS	tryptophanyl-tRNA synthetase	6.96	1.80E-05
IGFBP6	insulin-like growth factor binding protein 6	6.95	1.56E-04
PSMB8	proteasome (prosome, macropain) subunit, beta type, 8 (large multifunctional peptidase 7)	6.94	2.00E-05
PARP8	poly (ADP-ribose) polymerase family, member 8	6.94	2.38E-04
CACNA1A	calcium channel, voltage-dependent, P/Q type, alpha 1A subunit	6.93	7.00E-06
WISP1	WNT1 inducible signaling pathway protein 1	6.93	1.84E-03
UBA7	ubiquitin-like modifier activating enzyme 7	6.92	1.40E-05
SYNPO2	synaptopodin 2	6.90	9.18E-03
RASGRF2	Ras protein-specific guanine nucleotide-releasing factor 2	6.88	3.07E-03
DOCK1	Dedicator of cytokinesis 1	6.86	6.64E-03
BANK1	B-cell scaffold protein with ankyrin repeats 1	6.86	4.97E-04
TDRD7	tudor domain containing 7	6.85	3.06E-07
RAPGEF5	Rap guanine nucleotide exchange factor (GEF) 5	6.84	2.82E-03
PTPRC	protein tyrosine phosphatase, receptor type, C	6.84	8.88E-03
IKZF2	IKAROS family zinc finger 2 (Helios)	6.84	2.66E-04
BTN3A1	butyrophilin, subfamily 3, member A1	6.81	5.50E-05
TMEM171	transmembrane protein 171	6.81	1.48E-04
C19orf66	chromosome 19 open reading frame 66	6.78	1.04E-04
C5orf56	chromosome 5 open reading frame 56	6.78	1.34E-03
ZNF641	zinc finger protein 641	6.77	1.52E-03
TRIM69	tripartite motif containing 69	6.75	1.89E-04
UBA7	ubiquitin-like modifier activating enzyme 7	6.74	1.50E-05
PLSCR1	phospholipid scramblase 1	6.74	7.30E-05
IRS2	insulin receptor substrate 2	6.73	3.00E-06
PCDH17	protocadherin 17	6.72	4.37E-07
RCAN1	regulator of calcineurin 1	6.71	3.43E-03
C2CD4B	C2 calcium-dependent domain containing 4B	6.69	4.03E-04
COL8A1	collagen, type VIII, alpha 1	6.68	2.19E-03
IRF1	interferon regulatory factor 1	6.67	9.80E-05
CEACAM1	carcinoembryonic antigen-related cell adhesion molecule 1 (biliary glycoprotein)	6.67	3.15E-03
PCDH17	protocadherin 17	6.66	1.94E-04
NAMPT	nicotinamide phosphoribosyltransferase	6.65	7.00E-05
STARD5	StAR-related lipid transfer (START) domain containing 5	6.64	5.38E-04
LRAT	lecithin retinol acyltransferase (phosphatidylcholine--	6.64	5.57E-03

	retinol O-acyltransferase)		
POFUT1	protein O-fucosyltransferase 1	6.62	2.12E-03
PAPPA	pregnancy-associated plasma protein A, pappalysin 1	6.59	1.84E-04
TTC39A	tetratricopeptide repeat domain 39A	6.56	9.60E-05
BMF	Bcl2 modifying factor	6.56	2.88E-03
RCAN1	regulator of calcineurin 1	6.55	6.72E-04
ANKFN1	ankyrin-repeat and fibronectin type III domain containing 1	6.54	3.24E-03
FLJ39739	uncharacterized FLJ39739	6.54	3.30E-05
KYNU	kynureninase	6.53	8.96E-03
C1orf38	chromosome 1 open reading frame 38	6.53	7.00E-05
RBFOX3	RNA binding protein, fox-1 homolog (C. elegans) 3	6.44	4.96E-03
LTB	lymphotoxin beta (TNF superfamily, member 3)	6.41	4.47E-04
LOC170425	uncharacterized LOC170425	6.40	1.13E-03
PML	promyelocytic leukemia	6.35	1.50E-04
CXCR7	Chemokine (C-X-C motif) receptor 7	6.34	1.20E-04
TAP2	transporter 2, ATP-binding cassette, sub-family B (MDR/TAP)	6.31	7.91E-03
RNF213	ring finger protein 213	6.30	2.59E-03
HAS2	hyaluronan synthase 2	6.26	5.30E-05
TXNIP	thioredoxin interacting protein	6.25	3.34E-04
SLC41A2	solute carrier family 41, member 2	6.24	1.78E-07
MOCOS	molybdenum cofactor sulfurase	6.24	1.07E-04
TUBA3FP	tubulin, alpha 3f, pseudogene	6.23	2.50E-03
HLA-DOB	major histocompatibility complex, class II, DO beta	6.20	9.30E-05
LST1	leukocyte specific transcript 1	6.20	8.60E-04
GVINP1	GTPase, very large interferon inducible pseudogene 1	6.19	1.22E-04
SAMHD1	SAM domain and HD domain 1	6.19	2.30E-05
PTPRN2	protein tyrosine phosphatase, receptor type, N polypeptide 2	6.16	2.75E-03
IL7R	interleukin 7 receptor	6.15	9.00E-06
ZNFX1	zinc finger, NFX1-type containing 1	6.13	5.40E-05
RNF213	ring finger protein 213	6.12	1.80E-04
GUCY2D	guanylate cyclase 2D, membrane (retina-specific)	6.11	2.41E-03
RXFP1	relaxin/insulin-like family peptide receptor 1	6.11	3.23E-03
MT3	metallothionein 3	6.09	5.29E-03
LOC100287705 , PTN	uncharacterized LOC100287705 ; pleiotrophin	6.09	6.28E-04
SLC35F1	solute carrier family 35, member F1	6.09	5.28E-03
IL18R1	interleukin 18 receptor 1	6.02	2.00E-06
LRP2	low density lipoprotein receptor-related protein 2	6.01	6.03E-03
IFNE	interferon, epsilon	6.00	8.76E-04
ZNF396	zinc finger protein 396	6.00	3.24E-03
SOCS3	suppressor of cytokine signaling 3	5.97	1.37E-03
TMEM62	transmembrane protein 62	5.96	1.67E-07
NOVA2	neuro-oncological ventral antigen 2	5.95	6.56E-03
QTRTD1	queuine tRNA-ribosyltransferase domain containing 1	5.94	6.40E-04
SEMA3A	sema domain, immunoglobulin domain (Ig), short basic domain, secreted, (semaphorin) 3A	5.92	2.83E-04

APOL3	apolipoprotein L, 3	5.92	1.00E-06
CFB	complement factor B	5.91	2.78E-03
SLC25A28	solute carrier family 25 (mitochondrial iron transporter), member 28	5.91	5.00E-06
PCLO	piccolo (presynaptic cytomatrix protein)	5.89	7.13E-04
BTN3A2	butyrophilin, subfamily 3, member A2	5.87	1.52E-03
SH3RF3-AS1	SH3RF3 antisense RNA 1 (non-protein coding)	5.85	3.90E-04
OR4D2	olfactory receptor, family 4, subfamily D, member 2	5.84	3.99E-04
PML	promyelocytic leukemia	5.82	3.48E-03
LOC100507463	uncharacterized LOC100507463	5.81	5.00E-03
MAP3K8	mitogen-activated protein kinase kinase kinase 8	5.80	5.93E-04
SLFN5	schlafen family member 5	5.79	8.80E-05
UBQLNL	ubiquilin-like	5.77	7.32E-03
PPBP	pro-platelet basic protein (chemokine (C-X-C motif) ligand 7)	5.74	4.45E-04
LAP3	leucine aminopeptidase 3	5.73	7.00E-06
SRD5A3-AS1	SRD5A3 antisense RNA 1 (non-protein coding)	5.72	5.50E-05
JUNB	jun B proto-oncogene	5.69	2.78E-03
DTX3L	deltex 3-like (Drosophila)	5.69	3.00E-06
TRIM38	tripartite motif containing 38	5.68	1.77E-04
PPAP2B	phosphatidic acid phosphatase type 2B	5.67	1.94E-04
RNF213	ring finger protein 213	5.67	6.00E-06
SAT1	spermidine/spermine N1-acetyltransferase 1	5.60	3.19E-07
CXCL1	chemokine (C-X-C motif) ligand 1 (melanoma growth stimulating activity, alpha)	5.59	6.00E-06
KALRN	kalirin, RhoGEF kinase	5.59	8.62E-03
BTN3A3	butyrophilin, subfamily 3, member A3	5.56	1.60E-05
IL15RA	interleukin 15 receptor, alpha	5.54	1.10E-04
MIR155 , MIR155HG	microRNA 155 ; MIR155 host gene (non-protein coding)	5.52	3.10E-05
PARP9	poly (ADP-ribose) polymerase family, member 9	5.51	2.20E-05
EDNRB	endothelin receptor type B	5.49	1.83E-03
CXCR7	chemokine (C-X-C motif) receptor 7	5.46	6.70E-05
CLEC2D	C-type lectin domain family 2, member D	5.46	1.68E-03
PDGFRL	platelet-derived growth factor receptor-like	5.44	1.50E-05
BTN3A3	butyrophilin, subfamily 3, member A3	5.44	4.00E-06
PAPPA	pregnancy-associated plasma protein A, pappalysin 1	5.44	8.17E-04
MFSD12	major facilitator superfamily domain containing 12	5.44	4.57E-04
LOC100507535	uncharacterized LOC100507535	5.43	1.82E-04
LGMN	legumain	5.42	3.63E-03
PNPT1	polyribonucleotide nucleotidyltransferase 1	5.39	2.00E-04
RNF114	ring finger protein 114	5.38	3.24E-04
C5orf56	chromosome 5 open reading frame 56	5.36	1.71E-04
C1orf38	chromosome 1 open reading frame 38	5.35	5.96E-04
TMEM106A	transmembrane protein 106A	5.35	1.24E-04
ATF3	activating transcription factor 3	5.35	9.58E-03
EDNRB	endothelin receptor type B	5.33	1.13E-03
PPP2R2A	protein phosphatase 2, regulatory subunit B, alpha	5.32	9.75E-03
FOSL2	FOS-like antigen 2	5.31	4.21E-03

HLA-C	major histocompatibility complex, class I, C	5.31	4.80E-05
NAMPT	Nicotinamide phosphoribosyltransferase	5.30	6.80E-05
HLA-C	major histocompatibility complex, class I, C	5.29	9.44E-03
LST1	leukocyte specific transcript 1	5.29	1.59E-03
RHOC	Ras homolog family member C	5.28	4.00E-05
LOC283887	uncharacterized LOC283887	5.28	4.39E-03
MUC1	mucin 1, cell surface associated	5.25	5.25E-03
CFLAR	CASP8 and FADD-like apoptosis regulator	5.24	2.60E-03
OR8B8	olfactory receptor, family 8, subfamily B, member 8	5.24	7.06E-03
PMAIP1	phorbol-12-myristate-13-acetate-induced protein 1	5.21	1.30E-03
IRF8	interferon regulatory factor 8	5.19	7.34E-04
PLA2G4C	phospholipase A2, group IVC (cytosolic, calcium-independent)	5.18	6.70E-05
C19orf66	chromosome 19 open reading frame 66	5.17	6.50E-05
B2M	Beta-2-microglobulin	5.17	1.10E-03
KCNQ4	potassium voltage-gated channel, KQT-like subfamily, member 4	5.15	1.11E-03
GATA1	GATA binding protein 1 (globin transcription factor 1)	5.15	4.16E-03
JAK2	Janus kinase 2	5.14	7.84E-04
NOD2	nucleotide-binding oligomerization domain containing 2	5.14	3.42E-04
ZFP36	zinc finger protein 36, C3H type, homolog (mouse)	5.13	1.20E-03
C5orf56	chromosome 5 open reading frame 56	5.13	7.37E-03
LOC100288310	uncharacterized LOC100288310	5.12	3.55E-04
RHOH	ras homolog family member H	5.10	9.34E-03
TXNIP	thioredoxin interacting protein	5.08	1.20E-05
BLZF1	basic leucine zipper nuclear factor 1	5.05	3.60E-05
TXNIP	thioredoxin interacting protein	5.04	9.00E-06
DLL1	delta-like 1 (Drosophila)	5.04	9.30E-04
MOBP	myelin-associated oligodendrocyte basic protein	5.03	7.10E-04
SOX8	SRY (sex determining region Y)-box 8	4.99	3.69E-03
FAM160A1	family with sequence similarity 160, member A1	4.99	7.25E-03
CD47	CD47 molecule	4.99	4.95E-03
GPR65	G protein-coupled receptor 65	4.97	4.90E-03
APOD	apolipoprotein D	4.96	2.30E-05
C5orf56	chromosome 5 open reading frame 56	4.96	1.40E-05
PLA2G4A	phospholipase A2, group IVA (cytosolic, calcium-dependent)	4.94	8.00E-06
SLAMF9	SLAM family member 9	4.93	2.29E-03
SLC38A4	solute carrier family 38, member 4	4.90	1.27E-03
GXYLT2	glucoside xylosyltransferase 2	4.89	6.82E-03
LOC100507531	uncharacterized LOC100507531	4.89	3.23E-03
KIAA0664L3	KIAA0664-like 3	4.87	1.45E-03
UHRF1BP1	UHRF1 binding protein 1	4.87	8.41E-03
TAP2	transporter 2, ATP-binding cassette, sub-family B (MDR/TAP)	4.83	1.00E-05
GCA	grancalcin, EF-hand calcium binding protein	4.82	3.02E-03
SLC7A2	solute carrier family 7 (cationic amino acid transporter, y+ system), member 2	4.82	1.00E-06
PATL1	protein associated with topoisomerase II homolog 1 (yeast)	4.80	2.02E-04

CNKSR3	CNKSR family member 3	4.80	2.50E-05
GBP2	guanylate binding protein 2, interferon-inducible	4.78	2.95E-03
RHBDL2	rhomboid, veinlet-like 2 (Drosophila)	4.76	3.20E-03
BLZF1	basic leucine zipper nuclear factor 1	4.75	4.70E-05
NEDD9	neural precursor cell expressed, developmentally down-regulated 9	4.75	2.55E-03
TRAFD1	TRAF-type zinc finger domain containing 1	4.73	4.76E-04
VSIG10L	V-set and immunoglobulin domain containing 10 like	4.73	6.50E-05
SQRDL	sulfide quinone reductase-like (yeast)	4.72	3.60E-05
SEMA3C	sema domain, immunoglobulin domain (Ig), short basic domain, secreted, (semaphorin) 3C	4.71	1.20E-03
MIR137HG , MIR2682	MIR137 host gene (non-protein coding) ; microRNA 2682	4.70	1.40E-03
PARP10	poly (ADP-ribose) polymerase family, member 10	4.69	6.10E-05
C19orf66	chromosome 19 open reading frame 66	4.68	5.70E-05
BLZF1	basic leucine zipper nuclear factor 1	4.67	7.88E-04
KLF4	Kruppel-like factor 4 (gut)	4.67	4.24E-03
MR1	major histocompatibility complex, class I-related	4.66	3.40E-04
LAMA2	laminin, alpha 2	4.66	3.18E-03
PPAP2B	phosphatidic acid phosphatase type 2B	4.65	4.24E-04
L3MBTL4	l(3)mbt-like 4 (Drosophila)	4.65	1.15E-03
FOXF1	forkhead box F1	4.64	8.00E-06
CCDC82	coiled-coil domain containing 82	4.64	7.49E-03
IL15	interleukin 15	4.63	1.10E-05
FBXO32	F-box protein 32	4.61	1.17E-04
STAT1	signal transducer and activator of transcription 1, 91kDa	4.60	4.10E-05
RHEBL1	Ras homolog enriched in brain like 1	4.58	1.38E-03
TAF11	TAF11 RNA polymerase II, TATA box binding protein (TBP)-associated factor, 28kDa	4.57	7.43E-03
STAT2	signal transducer and activator of transcription 2, 113kDa	4.55	1.80E-05
N4BP1	NEDD4 binding protein 1	4.55	8.40E-05
PSMB10	proteasome (prosome, macropain) subunit, beta type, 10	4.54	3.20E-03
JAK2	Janus kinase 2	4.53	2.00E-06
GJD3	gap junction protein, delta 3, 31.9kDa	4.52	8.69E-04
CARD14	caspase recruitment domain family, member 14	4.52	9.12E-04
CGA	glycoprotein hormones, alpha polypeptide	4.50	7.53E-03
MOV10	Mov10, Moloney leukemia virus 10, homolog (mouse)	4.50	3.82E-03
HLA-A	major histocompatibility complex, class I, A	4.48	1.20E-05
NT5C3	5'-nucleotidase, cytosolic III	4.47	3.00E-06
LOC100289251	uncharacterized LOC100289251	4.47	9.02E-04
RGS2	regulator of G-protein signaling 2, 24kDa	4.46	9.00E-06
B2M	beta-2-microglobulin	4.46	3.00E-06
SLC41A2	solute carrier family 41, member 2	4.46	1.07E-07
KCNQ5	potassium voltage-gated channel, KQT-like subfamily, member 5	4.46	4.95E-03
IRS2	insulin receptor substrate 2	4.43	4.50E-05
IRAK3	interleukin-1 receptor-associated kinase 3	4.42	9.69E-04
SIGLEC16	sialic acid binding Ig-like lectin 16 (gene/pseudogene)	4.42	9.09E-03

NOL4	nucleolar protein 4	4.40	8.48E-03
INHA	inhibin, alpha	4.39	5.91E-03
IL4I1	interleukin 4 induced 1	4.38	5.58E-04
N4BP1	NEDD4 binding protein 1	4.37	2.00E-06
CD300LF	CD300 molecule-like family member f	4.37	8.86E-04
S1PR2	sphingosine-1-phosphate receptor 2	4.35	4.07E-04
ELF1	E74-like factor 1 (ets domain transcription factor)	4.34	4.23E-04
FLJ35024	uncharacterized LOC401491	4.34	1.92E-03
LOC100506831	uncharacterized LOC100506831	4.33	1.61E-03
TRAFD1	TRAF-type zinc finger domain containing 1	4.33	6.00E-06
CCL23	chemokine (C-C motif) ligand 23	4.32	6.49E-04
RGS8	regulator of G-protein signaling 8	4.31	6.84E-03
DICER1	dicer 1, ribonuclease type III	4.30	3.73E-03
CLDN1	claudin 1	4.30	2.00E-05
PCGF5	polycomb group ring finger 5	4.30	2.80E-04
MOBP	myelin-associated oligodendrocyte basic protein	4.30	5.66E-03
NMI	N-myc (and STAT) interactor	4.29	1.79E-07
PML	promyelocytic leukemia	4.29	1.11E-04
TRAFD1	TRAF-type zinc finger domain containing 1	4.29	1.29E-03
BLZF1	basic leucine zipper nuclear factor 1	4.28	8.87E-03
PPAP2B	phosphatidic acid phosphatase type 2B	4.28	4.00E-06
FAM105A	family with sequence similarity 105, member A	4.27	9.44E-03
KIAA1217	KIAA1217	4.27	2.00E-04
FAM122C	family with sequence similarity 122C	4.27	3.07E-04
TRIM25	tripartite motif containing 25	4.26	3.65E-04
WDFY4	WDFY family member 4	4.26	8.22E-04
ZC3H12C	zinc finger CCCH-type containing 12C	4.26	2.03E-04
SAMSN1	SAM domain, SH3 domain and nuclear localization signals 1	4.25	6.14E-04
IL15	interleukin 15	4.24	1.50E-05
ZBTB32	zinc finger and BTB domain containing 32	4.24	8.12E-03
BEX1	brain expressed, X-linked 1	4.23	8.00E-03
SYTL3	synaptotagmin-like 3	4.23	2.10E-05
RIPK2	receptor-interacting serine-threonine kinase 2	4.22	7.52E-04
TTC39B	tetratricopeptide repeat domain 39B	4.22	5.03E-04
SELL	selectin L	4.21	1.60E-03
LGALS8	lectin, galactoside-binding, soluble, 8	4.21	2.87E-04
NR4A2	nuclear receptor subfamily 4, group A, member 2	4.21	3.71E-03
HLA-G	major histocompatibility complex, class I, G	4.20	9.11E-03
FOXP2	forkhead box P2	4.20	9.43E-03
SLC18B1	solute carrier family 18, subfamily B, member 1	4.19	7.09E-03
CNTN2	contactin 2 (axonal)	4.19	2.09E-03
LYSMD2	LysM, putative peptidoglycan-binding, domain containing 2	4.18	5.18E-04
PCGF5	Polycomb group ring finger 5	4.18	6.95E-04
TREX1	three prime repair exonuclease 1	4.17	3.88E-04
APOBEC3F	apolipoprotein B mRNA editing enzyme, catalytic polypeptide-like 3F	4.16	1.31E-03
C4orf34	chromosome 4 open reading frame 34	4.16	1.49E-04

SLC25A37	solute carrier family 25 (mitochondrial iron transporter), member 37	4.16	5.29E-03
ZBTB42	zinc finger and BTB domain containing 42	4.15	4.33E-03
SLC25A28	solute carrier family 25 (mitochondrial iron transporter), member 28	4.14	3.44E-04
CFLAR	CASP8 and FADD-like apoptosis regulator	4.13	5.19E-04
AQP3	aquaporin 3 (Gill blood group)	4.13	2.91E-04
PML	promyelocytic leukemia	4.13	2.47E-03
LOC389834	ankyrin repeat domain 57 pseudogene	4.13	2.33E-03
SCARB2	scavenger receptor class B, member 2	4.10	2.40E-05
ACHE	acetylcholinesterase	4.09	2.91E-03
HLA-E	major histocompatibility complex, class I, E	4.08	2.10E-04
FAM26F	family with sequence similarity 26, member F	4.08	2.03E-03
TMEM106A	transmembrane protein 106A	4.08	3.80E-05
IL7R	interleukin 7 receptor	4.07	2.02E-03
PION	pigeon homolog (Drosophila)	4.07	1.29E-03
MOV10	Mov10, Moloney leukemia virus 10, homolog (mouse)	4.07	1.00E-05
CLIC2	chloride intracellular channel 2	4.05	6.49E-04
CD274	CD274 molecule	4.04	2.68E-04
NOX5	NADPH oxidase, EF-hand calcium binding domain 5	4.02	1.54E-03
TMEM92	transmembrane protein 92	4.02	4.01E-04
MYO16	myosin XVI	4.01	7.70E-05
TMEM140	transmembrane protein 140	4.01	1.08E-04
SLC25A37	solute carrier family 25 (mitochondrial iron transporter), member 37	4.00	1.89E-04

Down-regulated genes following RV-infection in HUVEC

Gene Symbol	Description	Fold Change	ANOVA p-value
RAB6B	RAB6B, member RAS oncogene family	-4.00	2.07E-03
JAG2	jagged 2	-4.01	3.59E-04
MYBL1	v-myb myeloblastosis viral oncogene homolog (avian)-like 1	-4.01	4.51E-04
DMP1	dentin matrix acidic phosphoprotein 1	-4.01	1.48E-04
RUNDC3B	RUN domain containing 3B	-4.04	3.58E-04
ARRB1	arrestin, beta 1	-4.05	2.28E-04
CCDC81	coiled-coil domain containing 81	-4.08	4.29E-03
LAMA3	laminin, alpha 3	-4.08	6.00E-03
HMGB3	high mobility group box 3	-4.10	5.75E-04
EPPK1	Epiplakin 1	-4.10	2.64E-03
SIM1	single-minded homolog 1 (Drosophila)	-4.13	3.43E-03
PURG	purine-rich element binding protein G	-4.13	2.81E-04
CDKN2B	cyclin-dependent kinase inhibitor 2B (p15, inhibits CDK4)	-4.13	7.13E-04
GPIHBP1	glycosylphosphatidylinositol anchored high density lipoprotein binding protein 1	-4.13	6.01E-03
RPL15	Ribosomal protein L15	-4.13	4.24E-04
GIPC2	GIPC PDZ domain containing family, member 2	-4.14	2.28E-04
ALDH1A2	aldehyde dehydrogenase 1 family, member A2	-4.16	1.30E-05
C20orf160	chromosome 20 open reading frame 160	-4.16	8.78E-03
LAT2	linker for activation of T cells family, member 2	-4.17	5.15E-03

CYYR1	cysteine/tyrosine-rich 1	-4.18	2.65E-04
C20orf112	chromosome 20 open reading frame 112	-4.18	4.80E-04
TTC3	tetratricopeptide repeat domain 3 ; tetratricopeptide repeat domain 3 pseudogene 1	-4.19	1.54E-04
GLRB	glycine receptor, beta	-4.19	1.99E-03
LOC100506942	uncharacterized LOC100506942	-4.20	4.12E-03
NEURL1B	neuralized homolog 1B (Drosophila)	-4.21	1.60E-03
FOXK1	forkhead box K1	-4.24	7.50E-03
RPS15A	ribosomal protein S15a	-4.25	2.30E-04
CLCN4	Chloride channel, voltage-sensitive 4	-4.27	7.85E-03
LOC100129550	uncharacterized LOC100129550	-4.28	3.90E-05
CRTAP	cartilage associated protein	-4.29	3.81E-04
THRB	thyroid hormone receptor, beta	-4.29	7.10E-03
EIF4E3	eukaryotic translation initiation factor 4E family member 3	-4.31	4.99E-04
PRKAA2	protein kinase, AMP-activated, alpha 2 catalytic subunit	-4.31	6.22E-03
RGS7BP	regulator of G-protein signaling 7 binding protein	-4.32	5.47E-03
IFNA2	interferon, alpha 2	-4.33	1.57E-04
CABLES1	Cdk5 and Abl enzyme substrate 1	-4.34	5.10E-05
MTL5	Metallothionein-like 5, testis-specific (tesmin)	-4.34	1.07E-03
NHS	Nance-Horan syndrome (congenital cataracts and dental anomalies)	-4.35	4.41E-04
DIO2	deiodinase, iodothyronine, type II	-4.36	2.00E-03
PAGE1	P antigen family, member 1 (prostate associated)	-4.36	8.00E-03
AK5	adenylate kinase 5	-4.36	2.50E-03
NXPH2	neurexophilin 2	-4.36	1.98E-03
SERPIND1	serpin peptidase inhibitor, clade D (heparin cofactor), member 1	-4.37	7.17E-04
LOC100505501	uncharacterized LOC100505501	-4.37	6.69E-04
FAXC	failed axon connections homolog (Drosophila)	-4.39	9.93E-03
C5orf4	chromosome 5 open reading frame 4	-4.41	9.69E-04
TSPAN12	tetraspanin 12	-4.42	2.07E-04
REPS2	RALBP1 associated Eps domain containing 2	-4.48	1.83E-04
EIF2C1	eukaryotic translation initiation factor 2C, 1	-4.48	1.03E-03
KRT19	Keratin 19	-4.49	1.61E-03
HMCN1	hemicentin 1	-4.51	8.55E-03
CCL16	chemokine (C-C motif) ligand 16	-4.52	5.12E-04
MMP16	matrix metallopeptidase 16 (membrane-inserted)	-4.53	1.00E-04
C14orf132	chromosome 14 open reading frame 132	-4.53	1.50E-03
RAB3B	RAB3B, member RAS oncogene family	-4.53	2.90E-05
PTPN2	Protein tyrosine phosphatase, non-receptor type 2	-4.53	9.55E-03
EFNA2	ephrin-A2	-4.54	5.89E-03
ZNF704	zinc finger protein 704	-4.57	2.04E-04
TMEM163	transmembrane protein 163	-4.57	4.15E-04
SPATA9	spermatogenesis associated 9	-4.58	9.87E-03
LOC100506870	uncharacterized LOC100506870	-4.59	7.81E-04
H2AFV	H2A histone family, member V	-4.60	1.74E-04
CHST9-AS1	CHST9 antisense RNA 1 (non-protein coding)	-4.62	6.23E-03
STX2	syntaxin 2	-4.64	4.37E-04

GNL1	guanine nucleotide binding protein-like 1	-4.65	2.01E-03
CYFIP2	cytoplasmic FMR1 interacting protein 2	-4.70	7.40E-04
ZMAT3	zinc finger, matrin-type 3	-4.70	1.17E-04
KCNJ15	potassium inwardly-rectifying channel, subfamily J, member 15	-4.72	9.25E-03
KLHL4	kelch-like 4 (Drosophila)	-4.72	1.93E-04
PPM1L	protein phosphatase, Mg2+/Mn2+ dependent, 1L	-4.72	2.10E-05
LOC285178	uncharacterized LOC285178	-4.73	3.03E-03
MEGF6	multiple EGF-like-domains 6	-4.75	4.50E-05
DANCR	differentiation antagonizing non-protein coding RNA	-4.76	2.00E-06
DMBX1	diencephalon/mesencephalon homeobox 1	-4.77	5.55E-03
NT5DC2	5'-nucleotidase domain containing 2	-4.78	5.70E-05
PRR7	proline rich 7 (synaptic)	-4.79	3.96E-03
C7orf41	chromosome 7 open reading frame 41	-4.79	1.47E-04
IPW	imprinted in Prader-Willi syndrome (non-protein coding) ; uncharacterized LOC100506948 ; small nucleolar RNA, C/D box 107 ; small nucleolar RNA, C/D box 115-13 ; small nucleolar RNA, C/D box 115-26 ; small nucleolar RNA, C/D box 115-7 ; small nucleolar RNA, C/D box 116-28	-4.82	1.61E-03
CRLS1	cardiolipin synthase 1	-4.84	9.05E-03
JAG2	jagged 2	-4.86	3.30E-04
ADAMTS18	ADAM metallopeptidase with thrombospondin type 1 motif, 18	-4.89	9.30E-05
C15orf32	chromosome 15 open reading frame 32	-4.89	2.58E-03
MIB2	Mindbomb E3 ubiquitin protein ligase 2	-4.90	1.27E-04
ENAH	enabled homolog (Drosophila)	-4.93	8.52E-04
MB21D1	Mab-21 domain containing 1	-4.94	8.11E-03
RNF17	ring finger protein 17	-4.98	1.53E-03
DIO2	deiodinase, iodothyronine, type II	-4.98	4.20E-04
ACE	angiotensin I converting enzyme (peptidyl-dipeptidase A) 1	-5.04	1.88E-03
ARHGAP6	Rho GTPase activating protein 6	-5.05	2.27E-03
SESN3	sestrin 3	-5.09	3.00E-04
HYDIN	HYDIN, axonemal central pair apparatus protein	-5.12	4.23E-03
LOC388942	uncharacterized LOC388942	-5.13	2.30E-03
CPA4	carboxypeptidase A4	-5.16	9.30E-05
HS6ST3	heparan sulfate 6-O-sulfotransferase 3	-5.16	5.28E-03
HMGA2	High mobility group AT-hook 2	-5.16	9.56E-03
ZSWIM2	zinc finger, SWIM-type containing 2	-5.20	5.80E-03
PARVA	parvin, alpha	-5.23	4.70E-05
THRB	thyroid hormone receptor, beta	-5.24	1.18E-04
FILIP1	filamin A interacting protein 1	-5.24	7.94E-03
PDE11A	phosphodiesterase 11A	-5.30	7.76E-04
AR	androgen receptor	-5.33	7.02E-04
PARD6G	par-6 partitioning defective 6 homolog gamma (C. elegans)	-5.33	4.30E-05
PLEKHG2	pleckstrin homology domain containing, family G (with RhoGef domain) member 2	-5.34	1.81E-04
TMEM170B	transmembrane protein 170B	-5.34	8.00E-06
KSR2	kinase suppressor of ras 2	-5.35	3.03E-04
TNFRSF10D	tumor necrosis factor receptor superfamily, member	-5.39	2.58E-04

	10d, decoy with truncated death domain		
ABCA13	ATP-binding cassette, sub-family A (ABC1), member 13	-5.45	3.96E-04
ZNF663	zinc finger protein 663	-5.46	3.33E-03
LOC100506548	uncharacterized LOC100506548 ; ribosomal protein L37	-5.47	1.22E-04
TMC5	transmembrane channel-like 5	-5.49	3.16E-03
UGT3A1	UDP glycosyltransferase 3 family, polypeptide A1	-5.50	5.27E-04
IPO9	importin 9	-5.51	1.80E-04
RBMXL2	RNA binding motif protein, X-linked-like 2	-5.59	3.12E-03
POU6F2-AS2	POU6F2 antisense RNA 2 (non-protein coding)	-5.62	1.84E-03
MYO7A	myosin VIIA	-5.65	6.10E-03
SERTAD4	SERTA domain containing 4	-5.65	1.68E-04
KCNIP3	Kv channel interacting protein 3, calsenilin	-5.66	2.07E-03
GGTA1P	glycoprotein, alpha-galactosyltransferase 1 pseudogene	-5.66	9.24E-03
KLRG1	killer cell lectin-like receptor subfamily G, member 1	-5.67	8.80E-03
CCR1	chemokine (C-C motif) receptor 1	-5.70	6.96E-03
TFDP2	transcription factor Dp-2 (E2F dimerization partner 2)	-5.71	1.51E-03
RNF125	ring finger protein 125, E3 ubiquitin protein ligase	-5.71	3.07E-03
MZF1	myeloid zinc finger 1	-5.73	1.58E-04
LOC57399	uncharacterized gastric protein ZA52P	-5.76	7.58E-03
GPR27	G protein-coupled receptor 27	-5.79	8.63E-03
SYT9	synaptotagmin IX	-5.82	8.44E-04
LRPAP1	low density lipoprotein receptor-related protein associated protein 1	-5.82	9.96E-04
FABP4	fatty acid binding protein 4, adipocyte	-5.83	2.00E-06
PDS5B	PDS5, regulator of cohesion maintenance, homolog B (S. cerevisiae)	-5.83	2.09E-04
SPINK6	serine peptidase inhibitor, Kazal type 6	-5.84	6.38E-03
TNS4	tensin 4	-5.89	4.80E-04
CEP70	centrosomal protein 70kDa	-5.91	1.74E-04
FAM205A	family with sequence similarity 205, member A ; family with sequence similarity 205, member B	-5.91	1.52E-03
MMP28	matrix metallopeptidase 28	-5.94	7.58E-03
PID1	phosphotyrosine interaction domain containing 1	-5.99	1.14E-03
KIAA1211	KIAA1211	-6.00	1.17E-04
ITGBL1	integrin, beta-like 1 (with EGF-like repeat domains)	-6.06	1.61E-04
PMEPA1	prostate transmembrane protein, androgen induced 1	-6.13	5.20E-05
AURKB	aurora kinase B	-6.17	4.05E-03
ARMC4	armadillo repeat containing 4	-6.20	9.71E-04
IL17RB	interleukin 17 receptor B	-6.22	8.79E-03
COL1A1	collagen, type I, alpha 1	-6.24	1.88E-03
KLRC1	killer cell lectin-like receptor subfamily C, member 1 ; killer cell lectin-like receptor subfamily C, member 2	-6.24	1.27E-03
PDGFRA	platelet-derived growth factor receptor, alpha polypeptide	-6.24	2.33E-03
HELLS	helicase, lymphoid-specific	-6.25	4.75E-03
MNT	MAX binding protein	-6.26	9.18E-03
POSTN	periostin, osteoblast specific factor	-6.27	3.70E-05
NLN	neurolysin (metallopeptidase M3 family)	-6.27	4.55E-04
PKI55	DKFZp434H1419	-6.30	2.18E-04

RBP1	retinol binding protein 1, cellular	-6.33	5.20E-05
PLCD3	phospholipase C, delta 3	-6.33	2.23E-03
SHISA2	shisa homolog 2 (Xenopus laevis)	-6.34	3.00E-06
SESN3	sestrin 3	-6.36	7.60E-05
FAM212B	family with sequence similarity 212, member B	-6.37	5.22E-04
NFYA	nuclear transcription factor Y, alpha	-6.38	9.00E-05
NEU3	sialidase 3 (membrane sialidase)	-6.39	1.00E-06
ESR1	estrogen receptor 1	-6.41	4.03E-03
ELN	elastin	-6.41	1.13E-03
KCNJ5	potassium inwardly-rectifying channel, subfamily J, member 5	-6.43	6.92E-03
PDK3	pyruvate dehydrogenase kinase, isozyme 3	-6.47	6.78E-03
SPTBN1	spectrin, beta, non-erythrocytic 1	-6.47	7.58E-04
GPR87	G protein-coupled receptor 87	-6.48	2.67E-03
RUNX1T1	runt-related transcription factor 1; translocated to, 1 (cyclin D-related)	-6.49	2.17E-03
ISYNA1	Inositol-3-phosphate synthase 1	-6.51	7.92E-03
GP5	glycoprotein V (platelet)	-6.52	1.96E-03
PARD3B	par-3 partitioning defective 3 homolog B (C. elegans)	-6.52	8.86E-04
PDZRN3	PDZ domain containing ring finger 3	-6.54	2.07E-03
AMIGO1	adhesion molecule with Ig-like domain 1	-6.58	2.86E-03
CNTNAP2	contactin associated protein-like 2	-6.61	3.80E-03
CDH19	cadherin 19, type 2	-6.61	2.91E-03
LRRC17	leucine rich repeat containing 17	-6.64	2.00E-06
NOG	noggin	-6.68	6.81E-03
OGN	osteoglycin	-6.72	3.31E-03
IGLV1-44	immunoglobulin lambda variable 1-44	-6.78	4.77E-03
SLC44A5	solute carrier family 44, member 5	-6.78	9.70E-05
NEGR1	neuronal growth regulator 1	-6.78	1.22E-03
CHD2	chromodomain helicase DNA binding protein 2	-6.79	1.52E-03
KAZN	kazrin, periplakin interacting protein	-6.80	2.30E-03
PTP4A1	protein tyrosine phosphatase type IVA, member 1	-6.80	8.14E-03
KIAA1549	KIAA1549	-6.83	9.03E-04
NAPSA	napsin A aspartic peptidase	-6.91	1.34E-03
ADCYAP1R1	adenylate cyclase activating polypeptide 1 (pituitary) receptor type I	-6.92	2.11E-03
PCDHGB8P	protocadherin gamma subfamily B, 8 pseudogene	-6.94	5.83E-03
CCDC48	coiled-coil domain containing 48	-6.97	9.41E-03
CWH43	cell wall biogenesis 43 C-terminal homolog (S. cerevisiae)	-6.99	6.16E-09
KRT80	keratin 80	-7.01	1.74E-04
EXOC4	exocyst complex component 4	-7.03	2.58E-03
COL27A1	collagen, type XXVII, alpha 1	-7.16	5.55E-03
C17orf51	chromosome 17 open reading frame 51 ; family with sequence similarity 27-like	-7.18	2.78E-03
WDR90	WD repeat domain 90	-7.29	2.36E-03
KIAA1211	KIAA1211	-7.31	6.17E-03
DEFB132	defensin, beta 132	-7.34	8.67E-03
LOC441178	uncharacterized LOC441178	-7.39	1.57E-03
KRT19	keratin 19	-7.40	2.61E-03

LOC100506965	uncharacterized LOC100506965	-7.40	1.31E-03
CCDC14	coiled-coil domain containing 14	-7.41	6.84E-03
CYP7B1	cytochrome P450, family 7, subfamily B, polypeptide 1	-7.62	3.54E-03
TNPO1	Transportin 1	-7.66	2.43E-03
NTRK3	neurotrophic tyrosine kinase, receptor, type 3	-7.70	6.13E-03
FBXL13	F-box and leucine-rich repeat protein 13	-7.71	4.07E-03
TSPAN18	tetraspanin 18	-7.72	1.88E-04
TRPM1	transient receptor potential cation channel, subfamily M, member 1	-7.72	9.07E-03
LPAR2	lysophosphatidic acid receptor 2	-7.74	6.44E-03
LOC388906	opioid growth factor receptor pseudogene	-7.86	9.04E-03
LRRC16A	leucine rich repeat containing 16A	-7.92	3.30E-03
EMCN	endomucin	-7.98	1.71E-07
ANKFN1	Ankyrin-repeat and fibronectin type III domain containing 1	-8.04	1.96E-03
CLN8	ceroid-lipofuscinosis, neuronal 8 (epilepsy, progressive with mental retardation)	-8.05	9.43E-04
NLN	neurolysin (metallopeptidase M3 family)	-8.06	7.41E-04
SV2C	synaptic vesicle glycoprotein 2C	-8.11	7.08E-04
SSX4	synovial sarcoma, X breakpoint 4 ; synovial sarcoma, X breakpoint 4B	-8.13	1.33E-04
PLA2G3	phospholipase A2, group III	-8.20	2.38E-03
C5orf48	chromosome 5 open reading frame 48	-8.20	5.17E-04
HIST1H2AK	histone cluster 1, H2ak	-8.40	1.34E-03
TMEM144	transmembrane protein 144	-8.56	3.61E-03
LOC100287375	uncharacterized LOC100287375	-8.57	4.57E-03
TRPM8	transient receptor potential cation channel, subfamily M, member 8	-8.73	7.91E-04
CXADR	coxsackie virus and adenovirus receptor	-8.78	9.98E-04
CDHR2	cadherin-related family member 2	-8.80	6.03E-03
RDH10	retinol dehydrogenase 10 (all-trans)	-9.00	1.89E-04
LRRC38	leucine rich repeat containing 38	-9.02	3.19E-03
FABP4	fatty acid binding protein 4, adipocyte	-9.04	4.52E-03
TTC23L	tetratricopeptide repeat domain 23-like	-9.06	2.27E-04
CCDC67	coiled-coil domain containing 67	-9.31	1.32E-03
HIST1H4A	histone cluster 1, H4a ; histone cluster 1, H4b ; histone cluster 1, H4c ; histone cluster 1, H4d ; histone cluster 1, H4e ; histone cluster 1, H4f ; histone cluster 1, H4h ; histone cluster 1, H4i ; histone cluster 1, H4j ; histone cluster 1, H4k ; histone cluster 1, H4l ; histone cluster 2, H4a ; histone cluster 2, H4b ; histone cluster 4, H4	-9.38	5.40E-05
POU3F3	POU class 3 homeobox 3	-9.46	6.38E-03
MYO3A	myosin IIIA	-9.46	2.69E-03
CD36	CD36 molecule (thrombospondin receptor)	-9.58	4.57E-03
ACSS3	acyl-CoA synthetase short-chain family member 3	-9.62	3.53E-04
EMR2	egf-like module containing, mucin-like, hormone receptor-like 2	-9.63	2.06E-03
LOC100128922	connexin	-9.68	8.95E-04
CXADR	coxsackie virus and adenovirus receptor	-9.68	4.90E-05
OR8G1	olfactory receptor, family 8, subfamily G, member 1	-9.71	4.60E-03
CLEC4GP1	C-type lectin domain family 4, member G pseudogene 1	-9.76	4.84E-03

SNTG1	syntrophin, gamma 1	-9.77	6.75E-04
RALGPS1	Ral GEF with PH domain and SH3 binding motif 1	-9.79	3.43E-03
SYCN	syncollin	-9.92	7.06E-03
DSCAM	Down syndrome cell adhesion molecule	-9.98	6.19E-03
FZD3	frizzled family receptor 3	-10.00	1.15E-04
FGFR2	fibroblast growth factor receptor 2	-10.01	2.38E-04
SYNGR1	synaptogyrin 1	-10.12	7.00E-06
MARC1	mitochondrial amidoxime reducing component 1	-10.23	1.80E-03
ZMYM2	zinc finger, MYM-type 2	-10.32	2.09E-04
TSPAN11	tetraspanin 11	-10.35	1.20E-05
C10orf128	chromosome 10 open reading frame 128	-10.63	7.13E-04
MMP16	matrix metallopeptidase 16 (membrane-inserted)	-10.70	2.00E-05
AQP1	aquaporin 1 (Colton blood group)	-11.05	9.75E-03
ADAMDEC1	ADAM-like, decysin 1	-11.07	1.14E-03
LOC100128594	uncharacterized LOC100128594	-11.31	9.41E-04
LINC00460	long intergenic non-protein coding RNA 460	-11.33	1.37E-03
LOC100127972	uncharacterized LOC100127972	-11.47	3.35E-03
SERPINB13	serpin peptidase inhibitor, clade B (ovalbumin), member 13	-11.83	7.31E-03
EML5	echinoderm microtubule associated protein like 5	-11.85	5.08E-03
CAMK4	calcium/calmodulin-dependent protein kinase IV	-11.86	6.13E-04
CIITA	class II, major histocompatibility complex, transactivator	-11.90	5.06E-03
ACO1	aconitase 1, soluble	-12.01	1.00E-06
KLHL14	kelch-like 14 (Drosophila)	-12.87	9.08E-04
MYO1A	myosin IA	-12.96	5.93E-03
RPL27A	ribosomal protein L27a ; small nucleolar RNA, H/ACA box 3	-13.09	1.71E-04
C18orf34	chromosome 18 open reading frame 34	-13.12	3.97E-04
TMEM35	transmembrane protein 35	-13.20	1.77E-03
SYT17	synaptotagmin XVII	-13.50	4.98E-04
TXK	TXK tyrosine kinase	-14.01	1.60E-03
LMO7	LIM domain 7	-14.06	1.85E-03
SEMA6A	sema domain, transmembrane domain (TM), and cytoplasmic domain, (semaphorin) 6A	-14.60	9.00E-06
SLC44A5	solute carrier family 44, member 5	-14.63	6.81E-03
NALCN	sodium leak channel, non-selective	-14.82	1.18E-03
BRF1	BRF1 homolog, subunit of RNA polymerase III transcription initiation factor IIIB (S. cerevisiae)	-15.53	6.89E-03
NALCN	sodium leak channel, non-selective	-15.77	3.52E-04
SPTBN2	spectrin, beta, non-erythrocytic 2	-16.23	9.20E-04
HMGA2	high mobility group AT-hook 2	-16.43	3.98E-03
SLC25A27	solute carrier family 25, member 27	-16.78	2.27E-03
NGB	neuroglobin	-16.94	3.30E-05
C1QTNF2	C1q and tumor necrosis factor related protein 2	-17.22	1.08E-03
MSR1	macrophage scavenger receptor 1	-18.24	1.39E-03
TRPM6	transient receptor potential cation channel, subfamily M, member 6	-18.74	2.21E-03
MRAP2	melanocortin 2 receptor accessory protein 2	-19.45	2.00E-06
CDH20	cadherin 20, type 2	-20.23	2.32E-03
CD36	CD36 molecule (thrombospondin receptor)	-20.76	2.99E-03

PCDHA2	protocadherin alpha 2	-21.05	5.53E-03
KCMF1	potassium channel modulatory factor 1	-21.12	5.22E-03
LHX2	LIM homeobox 2	-22.54	1.78E-03
STRBP	spermatid perinuclear RNA binding protein	-23.64	8.20E-03
COPG2IT1	COPG2 imprinted transcript 1 (non-protein coding)	-25.96	1.26E-03
REEP1	receptor accessory protein 1	-31.05	4.81E-03
FAM212B	family with sequence similarity 212, member B	-38.54	3.63E-04
AQP1	aquaporin 1 (Colton blood group)	-45.44	3.60E-05

Up-regulated genes following RV-infection in HSaVEC

Gene Symbol	Description	Fold Change	ANOVA p-value
IFNB1	interferon, beta 1, fibroblast	5925.38	1.76E-07
TNFAIP6	tumor necrosis factor, alpha-induced protein 6	1410.72	3.11E-04
SCN3A	sodium channel, voltage-gated, type III, alpha subunit	1222.11	1.24E-07
CCL4	chemokine (C-C motif) ligand 4	972.27	2.00E-05
CCL5	chemokine (C-C motif) ligand 5	430.30	2.00E-05
IL28A	interleukin 28A (interferon, lambda 2)	401.78	2.66E-04
C3	complement component 3	400.73	3.39E-04
IRG1	immunoresponsive 1 homolog (mouse)	364.32	7.78E-04
ATP10A	ATPase, class V, type 10A	361.00	1.08E-04
CCL5	chemokine (C-C motif) ligand 5	325.72	1.33E-07
CSAG2 , CSAG3	CSAG family, member 2 ; CSAG family, member 3	316.25	5.03E-04
CCL5	chemokine (C-C motif) ligand 5	310.81	5.00E-06
CXCL10	chemokine (C-X-C motif) ligand 10	297.78	8.69E-07
IDO1	indoleamine 2,3-dioxygenase 1	286.26	7.45E-04
CCL3 , CCL3L1 , CCL3L3	chemokine (C-C motif) ligand 3 ; chemokine (C-C motif) ligand 3-like 1 ; chemokine (C-C motif) ligand 3-like 3	278.09	3.00E-06
TAC3	tachykinin 3	253.27	3.27E-04
AIM2	absent in melanoma 2	220.77	7.15E-04
SAA1 , SAA2	serum amyloid A1 ; serum amyloid A2	187.80	6.40E-05
RSAD2	radical S-adenosyl methionine domain containing 2	182.63	1.39E-07
TLR2	toll-like receptor 2	172.71	1.74E-03
OASL	2'-5'-oligoadenylate synthetase-like	171.40	4.00E-06
OASL	2'-5'-oligoadenylate synthetase-like	165.46	1.30E-05
KLK10	kallikrein-related peptidase 10	162.81	1.90E-05
KLRD1	killer cell lectin-like receptor subfamily D, member 1	157.49	5.63E-04
CCL8	chemokine (C-C motif) ligand 8	151.42	3.50E-05
GBP4	guanylate binding protein 4	144.04	1.70E-05
GBP5	guanylate binding protein 5	143.96	1.00E-06
RNF175	ring finger protein 175	140.72	1.56E-03
NEURL3	neuralized homolog 3 (Drosophila) pseudogene	140.67	8.20E-05
MX2	myxovirus (influenza virus) resistance 2 (mouse)	137.19	3.00E-06
IL29	interleukin 29 (interferon, lambda 1)	124.90	1.36E-04
BTC	betacellulin	123.68	1.46E-10
TNFSF13B	tumor necrosis factor (ligand) superfamily, member 13b	122.63	3.80E-05
CCL20	chemokine (C-C motif) ligand 20	122.14	3.00E-06

SERPING1	serpin peptidase inhibitor, clade G (C1 inhibitor), member 1	119.19	1.40E-05
CFB	complement factor B	115.58	5.00E-06
LIF	leukemia inhibitory factor	113.04	3.72E-03
TNFSF13B	tumor necrosis factor (ligand) superfamily, member 13b	112.35	1.16E-04
TNFAIP6	tumor necrosis factor, alpha-induced protein 6	104.19	2.46E-03
HSH2D	hematopoietic SH2 domain containing	101.38	9.01E-07
TNFRSF9	tumor necrosis factor receptor superfamily, member 9	100.94	2.60E-05
HERC5	HECT and RLD domain containing E3 ubiquitin protein ligase 5	95.10	1.00E-05
BST2	bone marrow stromal cell antigen 2	94.60	8.00E-06
PI3	peptidase inhibitor 3, skin-derived	94.22	1.27E-03
FAM65B	family with sequence similarity 65, member B	93.85	2.65E-04
SGPP2	sphingosine-1-phosphate phosphatase 2	93.82	7.85E-04
IFIT2	interferon-induced protein with tetratricopeptide repeats 2	93.70	1.23E-04
IL1RN	interleukin 1 receptor antagonist	93.09	2.45E-03
RSAD2	radical S-adenosyl methionine domain containing 2	91.41	8.00E-06
LOC100129518 , SOD2	uncharacterized LOC100129518 ; superoxide dismutase 2, mitochondrial	91.33	7.55E-04
IFIT3	interferon-induced protein with tetratricopeptide repeats 3	90.79	1.00E-06
CXCL9	chemokine (C-X-C motif) ligand 9	88.71	1.37E-04
CD38	CD38 molecule	86.62	1.77E-07
CR1L	complement component (3b/4b) receptor 1-like	85.28	7.32E-04
IFITM1	interferon induced transmembrane protein 1	84.59	6.00E-06
C1S	complement component 1, s subcomponent	80.96	6.80E-05
TSLP	thymic stromal lymphopoietin	77.40	9.00E-06
SECTM1	secreted and transmembrane 1	76.55	5.00E-05
SLC27A6	solute carrier family 27 (fatty acid transporter), member 6	76.22	2.21E-03
KYNU	kynureninase	74.95	2.83E-03
CXCL11	chemokine (C-X-C motif) ligand 11	72.25	4.00E-06
ANGPTL1	angiopoietin-like 1	72.23	6.00E-06
C1R	complement component 1, r subcomponent	65.44	2.40E-05
KLRD1	killer cell lectin-like receptor subfamily D, member 1	64.97	8.00E-06
STATH	statherin	64.84	2.13E-03
ELOVL7	ELOVL fatty acid elongase 7	62.24	1.51E-03
EPSTI1	epithelial stromal interaction 1 (breast)	60.34	1.00E-06
LRP2	low density lipoprotein receptor-related protein 2	60.15	6.00E-06
TNFRSF9	tumor necrosis factor receptor superfamily, member 9	59.84	5.50E-05
C15orf48	chromosome 15 open reading frame 48	59.12	1.00E-06
SIDT1	SID1 transmembrane family, member 1	54.81	5.74E-03
KYNU	kynureninase	54.59	5.60E-05
CLEC4E	C-type lectin domain family 4, member E	53.94	7.86E-04
IFI30	interferon, gamma-inducible protein 30	53.88	8.00E-05
KYNU	kynureninase	53.57	9.00E-05
IFIT1	interferon-induced protein with tetratricopeptide repeats 1	53.39	5.00E-06
OR52K3P	olfactory receptor, family 52, subfamily K, member 3 pseudogene	53.39	6.77E-04

C1S	complement component 1, s subcomponent	50.78	2.02E-04
GPR84	G protein-coupled receptor 84	48.98	2.33E-04
TMEM139	transmembrane protein 139	48.84	6.02E-04
SOX8	SRY (sex determining region Y)-box 8	48.64	5.25E-03
BATF2	basic leucine zipper transcription factor, ATF-like 2	47.19	3.00E-06
RTP4	receptor (chemosensory) transporter protein 4	46.45	5.00E-06
IFITM1 , IFITM2	interferon induced transmembrane protein 1 ; interferon induced transmembrane protein 2	45.85	6.80E-05
KIR2DL2 , KIR2DL4 , KIR2DL5A , KIR2DL5B , KIR3DL3 , KIR3DS1 , LOC100287534	killer cell immunoglobulin-like receptor, two domains, long cytoplasmic tail, 2 ; killer cell immunoglobulin-like receptor, two domains, long cytoplasmic tail, 4 ; killer cell immunoglobulin-like receptor, two domains, long cytoplasmic tail, 5A ; killer cell immunoglobulin-like receptor, two domains, long cytoplasmic tail, 5B ; killer cell immunoglobulin-like receptor three domains long cytoplasmic tail 3 ; killer cell immunoglobulin-like receptor, three domains, short cytoplasmic tail, 1 ; killer cell immunoglobulin-like receptor 2DL4-like	45.36	7.42E-03
KIR2DL4	killer cell immunoglobulin-like receptor, two domains, long cytoplasmic tail, 4	44.49	2.87E-03
TNFAIP3	tumor necrosis factor, alpha-induced protein 3	44.00	2.80E-05
CSF2	colony stimulating factor 2 (granulocyte-macrophage)	43.76	4.19E-03
IFI44L	interferon-induced protein 44-like	43.41	7.27E-08
NCF4	neutrophil cytosolic factor 4, 40kDa	43.35	9.70E-03
IL12RB1	interleukin 12 receptor, beta 1	43.34	7.70E-05
IL28A , IL28B	interleukin 28A (interferon, lambda 2) ; interleukin 28B (interferon, lambda 3)	42.04	3.06E-04
MCHR1	melanin-concentrating hormone receptor 1	41.12	4.00E-06
ETV7	ets variant 7	39.70	7.00E-06
SYTL2	synaptotagmin-like 2	38.46	7.83E-03
HCAR3	hydroxycarboxylic acid receptor 3	37.24	9.10E-05
CMPK2	cytidine monophosphate (UMP-CMP) kinase 2, mitochondrial	37.15	4.00E-06
ATF3	activating transcription factor 3	37.15	3.87E-03
CX3CL1	chemokine (C-X3-C motif) ligand 1	36.99	5.00E-06
ATF3	activating transcription factor 3	36.88	2.40E-05
SAA1 , SAA2 , SAA2-SAA4	serum amyloid A1 ; serum amyloid A2 ; SAA2-SAA4 readthrough	36.43	2.00E-05
PI3	peptidase inhibitor 3, skin-derived	36.04	2.14E-03
NR4A3	nuclear receptor subfamily 4, group A, member 3	35.95	1.89E-03
SYNPO2	synaptopodin 2	35.33	9.38E-04
EGR3	early growth response 3	35.03	6.10E-05
ANGPTL1	angiopoietin-like 1	34.67	2.87E-03
EMR1	egf-like module containing, mucin-like, hormone receptor-like 1	34.29	2.24E-04
MRGPRX3	MAS-related GPR, member X3	34.15	2.90E-05
SEPT4	septin 4	34.14	1.04E-03
C8orf34	chromosome 8 open reading frame 34	34.08	1.75E-04
TEX14	testis expressed 14	33.98	4.47E-03
ZBTB32	zinc finger and BTB domain containing 32	33.96	1.43E-04
S100P	S100 calcium binding protein P	33.84	4.90E-05
IFI6	interferon, alpha-inducible protein 6	33.51	3.71E-04
KYNU	kynureninase	33.26	1.68E-04
HEPH	hephaestin	33.23	3.70E-03

CXCL3	chemokine (C-X-C motif) ligand 3	33.14	1.44E-04
CR1 , CR1L	complement component (3b/4b) receptor 1 (Knops blood group) ; complement component (3b/4b) receptor 1-like	33.06	3.81E-04
TMPRSS3	transmembrane protease, serine 3	32.90	8.51E-03
FGF5	fibroblast growth factor 5	32.70	3.00E-05
ANGPTL1	angiopoietin-like 1	32.63	1.40E-05
ETV7	ets variant 7	32.57	1.73E-03
CCL7	chemokine (C-C motif) ligand 7	32.34	4.40E-05
IFIH1	interferon induced with helicase C domain 1	32.31	2.00E-06
EPSTI1	epithelial stromal interaction 1 (breast)	32.25	4.00E-05
TMEM229B	transmembrane protein 229B	32.07	6.71E-03
TTLL6	tubulin tyrosine ligase-like family, member 6	31.94	2.82E-04
SP8	Sp8 transcription factor	31.80	2.14E-04
BANCR	BRAF-activated non-protein coding RNA	31.65	3.75E-04
GBP5	guanylate binding protein 5	31.39	2.80E-05
TBC1D1	TBC1 (tre-2/USP6, BUB2, cdc16) domain family, member 1	31.13	1.34E-04
SLC1A3	solute carrier family 1 (glial high affinity glutamate transporter), member 3	31.12	4.11E-03
GBP4	guanylate binding protein 4	30.67	2.00E-06
LRRN3	leucine rich repeat neuronal 3	30.38	5.13E-03
BRE-AS1	BRE antisense RNA 1 (non-protein coding)	30.16	1.30E-05
SERPINA3	serpin peptidase inhibitor, clade A (alpha-1 antiproteinase, antitrypsin), member 3	29.71	3.00E-06
ISG20	interferon stimulated exonuclease gene 20kDa	29.63	3.10E-05
CSF3	colony stimulating factor 3 (granulocyte)	29.57	3.00E-05
CCRN4L	CCR4 carbon catabolite repression 4-like (S. cerevisiae)	29.57	1.90E-05
SSTR2	somatostatin receptor 2	28.75	2.03E-04
CX3CL1	chemokine (C-X3-C motif) ligand 1	28.53	2.08E-03
ACTN2	actinin, alpha 2	28.12	2.44E-03
P2RY6	pyrimidinergic receptor P2Y, G-protein coupled, 6	28.04	4.56E-03
ISG20	interferon stimulated exonuclease gene 20kDa	27.96	4.10E-05
ZBP1	Z-DNA binding protein 1	27.59	1.00E-05
CDK18	cyclin-dependent kinase 18	27.22	1.11E-04
ODF3B	outer dense fiber of sperm tails 3B	26.74	8.29E-04
PTGS2	prostaglandin-endoperoxide synthase 2 (prostaglandin G/H synthase and cyclooxygenase)	26.64	5.20E-05
TNIP3	TNFAIP3 interacting protein 3	26.30	5.00E-06
PIK3AP1	phosphoinositide-3-kinase adaptor protein 1	25.96	2.40E-05
ADAM28	ADAM metallopeptidase domain 28	25.84	5.00E-06
RASGRP1	RAS guanyl releasing protein 1 (calcium and DAG-regulated)	25.59	7.83E-03
EDNRA	endothelin receptor type A	25.15	1.04E-04
TNFAIP3	tumor necrosis factor, alpha-induced protein 3	25.07	7.00E-06
KLRD1	killer cell lectin-like receptor subfamily D, member 1	24.67	1.58E-04
IFI35	interferon-induced protein 35	24.18	1.70E-05
BIRC3	baculoviral IAP repeat containing 3	24.14	1.02E-04
SLC15A3	solute carrier family 15, member 3	23.80	2.00E-06
IL1B	interleukin 1, beta	23.78	3.23E-04

LGALS3BP	lectin, galactoside-binding, soluble, 3 binding protein	23.45	1.00E-05
CA2	carbonic anhydrase II	23.44	1.00E-06
KMO	kynurenine 3-monooxygenase (kynurenine 3-hydroxylase)	23.15	7.75E-04
KMO	kynurenine 3-monooxygenase (kynurenine 3-hydroxylase)	23.03	8.81E-03
TBC1D1	TBC1 (tre-2/USP6, BUB2, cdc16) domain family, member 1	23.00	1.18E-04
PARP10	poly (ADP-ribose) polymerase family, member 10	22.95	2.37E-04
KIR2DL4	killer cell immunoglobulin-like receptor, two domains, long cytoplasmic tail, 4	22.56	1.62E-04
MSC	musculin	22.33	2.00E-06
PDZK1IP1	PDZK1 interacting protein 1	22.29	5.10E-05
HAS2	hyaluronan synthase 2	21.91	9.02E-04
MX1	myxovirus (influenza virus) resistance 1, interferon-inducible protein p78 (mouse)	21.90	4.00E-05
C2	complement component 2	21.81	7.76E-03
CSF2	colony stimulating factor 2 (granulocyte-macrophage)	21.57	7.73E-04
RET	ret proto-oncogene	21.57	3.46E-03
BTC	betacellulin	21.54	4.31E-04
MAB21L2	mab-21-like 2 (C. elegans)	21.51	2.73E-03
ODF3B	outer dense fiber of sperm tails 3B	21.40	3.66E-04
CLDN23	claudin 23	21.35	3.00E-06
IL2RG	interleukin 2 receptor, gamma	21.34	1.06E-03
MAP3K8	mitogen-activated protein kinase kinase kinase 8	21.14	2.20E-05
RBM47	RNA binding motif protein 47	20.95	1.49E-03
HNF4G	hepatocyte nuclear factor 4, gamma	20.43	8.86E-03
CYP1B1	cytochrome P450, family 1, subfamily B, polypeptide 1	20.15	8.66E-03
LOC100129518 , SOD2	uncharacterized LOC100129518 ; superoxide dismutase 2, mitochondrial	20.13	5.41E-04
JUP , KRT17	junction plakoglobin ; keratin 17	19.85	2.73E-04
ZMYND15	zinc finger, MYND-type containing 15	19.21	1.41E-03
C12orf50	chromosome 12 open reading frame 50	19.10	1.18E-03
EGR2	early growth response 2	19.01	4.25E-04
FGL1	fibrinogen-like 1	18.96	3.55E-04
FST	follistatin	18.58	2.50E-05
F3	coagulation factor III (thromboplastin, tissue factor)	18.54	4.70E-05
FBXO32	F-box protein 32	18.47	5.00E-06
PRIC285	peroxisomal proliferator-activated receptor A interacting complex 285	18.45	6.00E-06
PLA1A	phospholipase A1 member A	18.26	1.50E-04
NKX3-1	NK3 homeobox 1	18.20	8.10E-05
IL1B	interleukin 1, beta	18.10	9.80E-05
CYP1B1	cytochrome P450, family 1, subfamily B, polypeptide 1	18.03	4.30E-05
GBP1	guanylate binding protein 1, interferon-inducible	17.94	1.10E-05
HCG4	HLA complex group 4 (non-protein coding)	17.94	3.00E-05
ICAM1	intercellular adhesion molecule 1	17.89	6.60E-05
FST	follistatin	17.88	5.00E-05
OTOF	otoferlin	17.66	4.10E-05
PDE6H	phosphodiesterase 6H, cGMP-specific, cone, gamma	17.52	5.80E-05

KLF4	Kruppel-like factor 4 (gut)	17.34	1.70E-05
IFIT3	interferon-induced protein with tetratricopeptide repeats 3	17.25	2.00E-06
GBP1	guanylate binding protein 1, interferon-inducible	17.24	2.00E-06
LRRN3	leucine rich repeat neuronal 3	17.19	3.69E-03
CPA2	carboxypeptidase A2 (pancreatic)	16.99	4.62E-04
IL6	interleukin 6 (interferon, beta 2)	16.96	4.10E-05
RRN3P1	RNA polymerase I transcription factor homolog (S. cerevisiae) pseudogene 1	16.84	1.56E-03
PLEKHA4	pleckstrin homology domain containing, family A (phosphoinositide binding specific) member 4	16.75	1.29E-04
XAF1	XIAP associated factor 1	16.61	5.30E-05
IL18BP	interleukin 18 binding protein	16.60	1.46E-04
LRP2	low density lipoprotein receptor-related protein 2	16.13	1.05E-04
SAA2-SAA4 , SAA4	SAA2-SAA4 readthrough ; serum amyloid A4, constitutive	16.11	1.09E-03
FAM46A	family with sequence similarity 46, member A	16.10	2.20E-05
CXCL11	chemokine (C-X-C motif) ligand 11	15.99	2.10E-05
CLDN23	claudin 23	15.93	1.37E-04
FST	follistatin	15.79	1.40E-05
IRF8	interferon regulatory factor 8	15.78	1.27E-03
SAMD9	sterile alpha motif domain containing 9	15.68	3.60E-07
GBP1	guanylate binding protein 1, interferon-inducible	15.67	3.77E-03
MCF2	MCF.2 cell line derived transforming sequence	15.65	1.83E-04
VNN3	vanin 3	15.65	1.74E-03
APCDD1	adenomatosis polyposis coli down-regulated 1	15.61	7.32E-03
ISG15	ISG15 ubiquitin-like modifier	15.54	1.80E-05
CEBPD	CCAAT/enhancer binding protein (C/EBP), delta	15.41	8.92E-07
PDZK1IP1	PDZK1 interacting protein 1	15.39	1.53E-04
CXCL5	chemokine (C-X-C motif) ligand 5	15.34	9.50E-05
IFIT2	interferon-induced protein with tetratricopeptide repeats 2	15.30	6.00E-06
SLC22A15	solute carrier family 22, member 15	15.15	2.98E-03
AK8	adenylate kinase 8	15.11	1.70E-03
DGKG	diacylglycerol kinase, gamma 90kDa	15.07	2.18E-03
NCF2	neutrophil cytosolic factor 2	15.03	2.59E-04
LOC100507108	uncharacterized LOC100507108	14.89	1.26E-03
N4BP2L1	NEDD4 binding protein 2-like 1	14.38	1.75E-04
FAM65C	family with sequence similarity 65, member C	14.38	7.11E-03
EBI3	Epstein-Barr virus induced 3	14.28	8.01E-03
CD69	CD69 molecule	14.24	1.80E-05
HLA-G	major histocompatibility complex, class I, G	14.16	2.91E-03
GPR65	G protein-coupled receptor 65	14.08	2.24E-03
XAF1	XIAP associated factor 1	14.03	3.90E-05
OLFM3	olfactomedin 3	13.91	7.36E-04
ICAM1	intercellular adhesion molecule 1	13.85	4.00E-06
FAM26F	family with sequence similarity 26, member F	13.49	1.59E-04
ODF3B	outer dense fiber of sperm tails 3B	13.47	9.30E-05
ACHE	acetylcholinesterase	13.33	4.64E-03
IFIH1	interferon induced with helicase C domain 1	13.27	3.00E-06

IGHA1	Immunoglobulin heavy constant alpha 1	13.26	1.84E-03
BTN2A3P	butyrophilin, subfamily 2, member A3, pseudogene	13.20	2.09E-03
PARP8	poly (ADP-ribose) polymerase family, member 8	13.19	6.83E-04
RND1	Rho family GTPase 1	13.17	9.48E-03
APOL6	apolipoprotein L, 6	13.15	3.00E-06
LOC100129518 , SOD2	uncharacterized LOC100129518 ; superoxide dismutase 2, mitochondrial	13.13	3.30E-05
CXorf65	chromosome X open reading frame 65	13.04	3.40E-03
C1orf95	chromosome 1 open reading frame 95	13.01	2.07E-03
SHANK2	SH3 and multiple ankyrin repeat domains 2	12.95	6.88E-03
LOC100287705 , PTN	uncharacterized LOC100287705 ; pleiotrophin	12.78	1.27E-03
FAM91A1	family with sequence similarity 91, member A1	12.68	9.53E-03
LOC729083	uncharacterized LOC729083	12.68	3.64E-03
LOC100128988	uncharacterized LOC100128988	12.66	1.25E-03
COL8A1	collagen, type VIII, alpha 1	12.63	1.95E-04
GABBR1 , UBD	gamma-aminobutyric acid (GABA) B receptor, 1 ; ubiquitin D	12.36	4.30E-05
BDKRB2	bradykinin receptor B2	12.30	5.19E-04
SAG	S-antigen; retina and pineal gland (arrestin)	12.29	3.73E-04
STAT2	signal transducer and activator of transcription 2, 113kDa	12.28	6.92E-04
PRRG4	proline rich Gla (G-carboxyglutamic acid) 4 (transmembrane)	12.25	1.00E-06
GBP1	guanylate binding protein 1, interferon-inducible	12.17	2.40E-05
LOC100129518 , SOD2	uncharacterized LOC100129518 ; superoxide dismutase 2, mitochondrial	12.15	5.50E-05
CH25H	cholesterol 25-hydroxylase	12.11	1.37E-04
IL1A	interleukin 1, alpha	12.08	1.34E-04
SRD5A3-AS1	SRD5A3 antisense RNA 1 (non-protein coding)	12.06	2.40E-04
OAS3	2'-5'-oligoadenylate synthetase 3, 100kDa	11.97	3.00E-06
LRG1	leucine-rich alpha-2-glycoprotein 1	11.92	6.08E-04
FARP2	FERM, RhoGEF and pleckstrin domain protein 2	11.79	1.84E-04
TULP2	tubby like protein 2	11.72	1.19E-03
SYNPO2	synaptopodin 2	11.72	3.35E-04
IFNA17	interferon, alpha 17	11.70	1.60E-04
RNF213	ring finger protein 213	11.68	5.27E-04
H2AFB1 , H2AFB2 , H2AFB3	H2A histone family, member B1 ; H2A histone family, member B2 ; H2A histone family, member B3	11.60	5.20E-05
ADAP1	ArfGAP with dual PH domains 1	11.54	1.60E-04
CTSS	cathepsin S	11.53	1.00E-06
CLEC4D	C-type lectin domain family 4, member D	11.53	2.47E-03
COL1A1	collagen, type I, alpha 1	11.50	3.79E-03
HERC6	HECT and RLD domain containing E3 ubiquitin protein ligase family member 6	11.35	4.00E-06
HIST2H2BE	histone cluster 2, H2be	11.30	6.80E-05
OAS2	2'-5'-oligoadenylate synthetase 2, 69/71kDa	11.30	3.83E-04
KL	klotho	11.25	8.35E-03
IRF7	interferon regulatory factor 7	11.23	3.70E-05
SLC7A2	solute carrier family 7 (cationic amino acid transporter, y+ system), member 2	11.02	2.70E-05
ACSL4	Acyl-CoA synthetase long-chain family member 4	11.02	9.00E-05

SELE	selectin E	10.93	1.50E-05
VNN3	vanin 3	10.83	2.28E-04
OAS2	2'-5'-oligoadenylate synthetase 2, 69/71kDa	10.82	1.10E-04
GJD3	gap junction protein, delta 3, 31.9kDa	10.75	3.50E-05
PPM1K	protein phosphatase, Mg2+/Mn2+ dependent, 1K	10.75	8.10E-05
OAS1	2'-5'-oligoadenylate synthetase 1, 40/46kDa	10.70	6.30E-05
LAMA2	laminin, alpha 2	10.67	3.13E-04
LOC285628 , MIR146A	uncharacterized LOC285628 ; microRNA 146a	10.66	3.50E-05
PROL1	proline rich, lacrimal 1	10.65	1.13E-03
CD33	CD33 molecule	10.64	8.20E-05
ARAP2	ArfGAP with RhoGAP domain, ankyrin repeat and PH domain 2	10.64	6.66E-07
EDNRA	endothelin receptor type A	10.62	6.97E-04
C6orf58	chromosome 6 open reading frame 58	10.62	7.20E-03
RNF213	ring finger protein 213	10.53	6.32E-07
FXYD6	FXYD domain containing ion transport regulator 6	10.52	4.39E-03
LY6E	lymphocyte antigen 6 complex, locus E	10.49	1.13E-04
TFAP2A	transcription factor AP-2 alpha (activating enhancer binding protein 2 alpha)	10.49	5.69E-04
RSPO3	R-spondin 3	10.47	1.20E-05
RBM47	RNA binding motif protein 47	10.45	4.57E-03
LOC100271840	uncharacterized LOC100271840	10.45	3.55E-03
DDX58	DEAD (Asp-Glu-Ala-Asp) box polypeptide 58	10.43	1.20E-05
TLR3	toll-like receptor 3	10.41	3.00E-05
PRRG4	proline rich Gla (G-carboxyglutamic acid) 4 (transmembrane)	10.39	1.49E-03
RUNX3	runt-related transcription factor 3	10.37	3.92E-03
FAM163A	family with sequence similarity 163, member A	10.37	1.76E-03
PAX3	paired box 3	10.37	1.40E-03
ZC3H12A	zinc finger CCCH-type containing 12A	10.35	1.10E-05
TAC1	tachykinin, precursor 1	10.33	1.96E-03
ST14	suppression of tumorigenicity 14 (colon carcinoma)	10.33	1.00E-03
SYNPO2	synaptopodin 2	10.32	2.40E-04
CYP2J2	cytochrome P450, family 2, subfamily J, polypeptide 2	10.31	1.48E-04
SLITRK5	SLIT and NTRK-like family, member 5	10.31	9.01E-03
VNN2	vanin 2	10.20	2.41E-03
SLAMF8	SLAM family member 8	10.16	4.42E-03
IL12A	interleukin 12A (natural killer cell stimulatory factor 1, cytotoxic lymphocyte maturation factor 1, p35)	10.15	1.04E-03
MAB21L2	mab-21-like 2 (C. elegans)	10.15	1.40E-05
ANGPTL4	angiopoietin-like 4	10.10	1.44E-04
VCAM1	vascular cell adhesion molecule 1	10.08	1.70E-05
PTPRC	protein tyrosine phosphatase, receptor type, C	10.06	6.69E-03
SIX1	SIX homeobox 1	10.03	8.69E-03
HIST2H2AA3 , HIST2H2AA4	histone cluster 2, H2aa3 ; histone cluster 2, H2aa4	9.98	1.74E-04
IL3RA	interleukin 3 receptor, alpha (low affinity)	9.94	7.90E-05
PDZD2	PDZ domain containing 2	9.90	6.74E-04
DENND2D	DENN/MADD domain containing 2D	9.88	1.60E-04

PCLO	piccolo (presynaptic cytomatrix protein)	9.81	1.06E-03
TRPC4	transient receptor potential cation channel, subfamily C, member 4	9.81	3.76E-04
SOCS1	suppressor of cytokine signaling 1	9.79	2.13E-03
LOC100507472 , PCSK6	uncharacterized LOC100507472 ; proprotein convertase subtilisin/kexin type 6	9.79	3.94E-04
CD83	CD83 molecule	9.77	1.58E-03
LOC100507307	uncharacterized LOC100507307	9.69	1.03E-04
CFB	complement factor B	9.57	1.51E-03
SERPINE2	Serpin peptidase inhibitor, clade E (nexin, plasminogen activator inhibitor type 1), member 2	9.56	2.74E-03
ITGA11	integrin, alpha 11	9.52	4.87E-03
CASZ1	castor zinc finger 1	9.44	2.65E-03
LITAF	lipopolysaccharide-induced TNF factor	9.42	2.72E-04
RUNX2	runt-related transcription factor 2	9.42	3.83E-04
SAMHD1	SAM domain and HD domain 1	9.42	2.03E-04
CASP1	caspase 1, apoptosis-related cysteine peptidase	9.41	1.30E-05
PARP8	poly (ADP-ribose) polymerase family, member 8	9.40	1.90E-05
TRIM14	tripartite motif containing 14	9.39	1.30E-05
KLF4	Kruppel-like factor 4 (gut)	9.39	1.52E-04
SLC7A2	solute carrier family 7 (cationic amino acid transporter, y+ system), member 2	9.35	4.58E-04
DDO	D-aspartate oxidase	9.33	1.49E-03
SLAMF7	SLAM family member 7	9.31	1.31E-03
C12orf39	chromosome 12 open reading frame 39	9.31	1.69E-04
PMAIP1	phorbol-12-myristate-13-acetate-induced protein 1	9.29	1.86E-04
APOBEC3G	apolipoprotein B mRNA editing enzyme, catalytic polypeptide-like 3G	9.25	8.40E-05
ZNF500	zinc finger protein 500	9.24	1.60E-05
		9.24	7.06E-04
ERV9-1	endogenous retrovirus group 9, member 1	9.21	6.53E-03
LOC100130992	uncharacterized LOC100130992	9.13	5.11E-03
FAM46A	family with sequence similarity 46, member A	9.09	1.74E-04
CLEC7A	C-type lectin domain family 7, member A	9.07	7.80E-04
FAM111A	family with sequence similarity 111, member A	9.04	5.01E-03
OAS3	2'-5'-oligoadenylate synthetase 3, 100kDa	9.04	3.00E-06
GALE	UDP-galactose-4-epimerase	9.03	7.48E-03
CXCL6	chemokine (C-X-C motif) ligand 6 (granulocyte chemotactic protein 2)	8.97	5.07E-04
HLA-DQB1	major histocompatibility complex, class II, DQ beta 1	8.93	3.37E-04
LOC731424	uncharacterized LOC731424	8.89	1.60E-05
BANK1	B-cell scaffold protein with ankyrin repeats 1	8.88	4.66E-04
CEBPD	CCAAT/enhancer binding protein (C/EBP), delta	8.86	1.05E-04
CAPN10	calpain 10	8.83	3.71E-03
IL17F	interleukin 17F	8.80	8.17E-04
KIAA0664L3	KIAA0664-like 3	8.80	9.60E-05
SIK1	salt-inducible kinase 1	8.78	2.11E-03
ICAM1	intercellular adhesion molecule 1	8.77	6.00E-06
ATP6V1B1	ATPase, H+ transporting, lysosomal 56/58kDa, V1 subunit B1	8.77	2.55E-04
CEACAM1	carcinoembryonic antigen-related cell adhesion molecule 1 (biliary glycoprotein)	8.74	2.80E-05

CST6	cystatin E/M	8.72	7.24E-03
PLEKHB1	Pleckstrin homology domain containing, family B (evectins) member 1	8.67	2.03E-03
LOC440896	uncharacterized LOC440896	8.65	1.47E-03
TTR	transthyretin	8.62	3.22E-04
WISP1	WNT1 inducible signaling pathway protein 1	8.60	6.15E-03
TNFSF10	tumor necrosis factor (ligand) superfamily, member 10	8.59	1.80E-05
CYP1B1	cytochrome P450, family 1, subfamily B, polypeptide 1	8.58	1.37E-03
SNX29	sorting nexin 29	8.54	5.83E-03
SAMHD1	SAM domain and HD domain 1	8.53	7.60E-05
ZFP36	zinc finger protein 36, C3H type, homolog (mouse)	8.51	5.41E-04
PLSCR1	phospholipid scramblase 1	8.51	5.00E-06
KIAA0146	KIAA0146	8.50	7.00E-06
ADCY1	adenylate cyclase 1 (brain)	8.44	7.85E-04
GEM	GTP binding protein overexpressed in skeletal muscle	8.43	1.39E-03
CLDN14	claudin 14	8.41	3.22E-04
FBXO32	F-box protein 32	8.41	9.80E-05
DSP	desmoplakin	8.38	9.13E-04
NPAS3	neuronal PAS domain protein 3	8.38	2.54E-03
HIST2H2AA3 , HIST2H2AA4	histone cluster 2, H2aa3 ; histone cluster 2, H2aa4	8.33	2.00E-05
DHRS2	dehydrogenase/reductase (SDR family) member 2	8.32	8.24E-03
TRAF1	TNF receptor-associated factor 1	8.30	5.08E-04
DDX58	DEAD (Asp-Glu-Ala-Asp) box polypeptide 58	8.29	1.36E-04
MSX1	msh homeobox 1	8.28	2.00E-06
PAPPA	pregnancy-associated plasma protein A, pappalysin 1	8.24	1.54E-03
LOC100130458	uncharacterized LOC100130458	8.24	6.91E-03
CYP7A1	cytochrome P450, family 7, subfamily A, polypeptide 1	8.23	9.80E-03
SYNPO2L	synaptopodin 2-like	8.21	1.05E-03
USP18	ubiquitin specific peptidase 18	8.18	1.62E-07
ANGPTL4	angiopoietin-like 4	8.17	6.30E-05
HCP5	HLA complex P5 (non-protein coding)	8.16	4.00E-06
MXD1	MAX dimerization protein 1	8.15	1.65E-03
RAB39B	RAB39B, member RAS oncogene family	8.12	9.89E-03
NFKBIZ	nuclear factor of kappa light polypeptide gene enhancer in B-cells inhibitor, zeta	8.08	7.90E-05
FCGR2A	Fc fragment of IgG, low affinity IIa, receptor (CD32)	8.06	4.40E-05
RNF213	ring finger protein 213	8.03	1.50E-03
CASP1	caspase 1, apoptosis-related cysteine peptidase	8.02	4.54E-07
TRIM14	tripartite motif containing 14	8.01	9.40E-07
MILR1	mast cell immunoglobulin-like receptor 1	8.01	1.77E-03
SYNPO2	synaptopodin 2	8.01	2.46E-03
KIAA0146	KIAA0146	7.99	3.90E-05
LOC284294	uncharacterized LOC284294	7.98	7.88E-03
EPPK1	epiplakin 1	7.97	6.18E-04
ZC3HAV1	zinc finger CCCH-type, antiviral 1	7.93	5.40E-05
FBXO6	F-box protein 6	7.90	1.90E-05

IL18RAP	interleukin 18 receptor accessory protein	7.87	2.39E-03
ZNF192	zinc finger protein 192	7.79	1.01E-03
JAK3	Janus kinase 3	7.79	2.70E-05
RHEBL1	Ras homolog enriched in brain like 1	7.73	9.00E-06
LHX8	LIM homeobox 8	7.68	2.57E-03
KLK15	kallikrein-related peptidase 15	7.64	5.60E-04
SP110	SP110 nuclear body protein	7.63	1.00E-04
BIRC3	baculoviral IAP repeat containing 3	7.62	4.96E-03
CEACAM1	carcinoembryonic antigen-related cell adhesion molecule 1 (biliary glycoprotein)	7.61	1.01E-03
LPL	lipoprotein lipase	7.60	2.42E-03
HIST1H4A , HIST1H4B , HIST1H4C , HIST1H4D , HIST1H4E , HIST1H4F , HIST1H4H , HIST1H4I , HIST1H4J , HIST1H4K , HIST1H4L , HIST2H4A , HIST2H4B , HIST4H4	histone cluster 1, H4a ; histone cluster 1, H4b ; histone cluster 1, H4c ; histone cluster 1, H4d ; histone cluster 1, H4e ; histone cluster 1, H4f ; histone cluster 1, H4h ; histone cluster 1, H4i ; histone cluster 1, H4j ; histone cluster 1, H4k ; histone cluster 1, H4l ; histone cluster 2, H4a ; histone cluster 2, H4b ; histone cluster 4, H4	7.60	3.07E-03
ZDHHC2	Zinc finger, DHHC-type containing 2	7.59	7.64E-03
PKIB	protein kinase (cAMP-dependent, catalytic) inhibitor beta	7.58	2.70E-03
CDH3	cadherin 3, type 1, P-cadherin (placental)	7.52	9.29E-03
LITAF	lipopolysaccharide-induced TNF factor	7.51	3.26E-03
PSMB9	proteasome (prosome, macropain) subunit, beta type, 9 (large multifunctional peptidase 2)	7.51	2.10E-05
SGPP2	sphingosine-1-phosphate phosphatase 2	7.51	3.20E-05
XRN1	5'-3' exoribonuclease 1	7.51	2.51E-03
RABGAP1L	RAB GTPase activating protein 1-like	7.49	8.50E-05
C1QTNF1	C1q and tumor necrosis factor related protein 1	7.48	3.27E-03
TNFAIP2	tumor necrosis factor, alpha-induced protein 2	7.47	2.90E-05
SERPINA1	serpin peptidase inhibitor, clade A (alpha-1 antiproteinase, antitrypsin), member 1	7.46	2.51E-03
LAMA2	laminin, alpha 2	7.45	1.03E-03
IFI44	Interferon-induced protein 44	7.42	8.00E-06
ARL14	ADP-ribosylation factor-like 14	7.40	2.14E-03
BLZF1	basic leucine zipper nuclear factor 1	7.38	4.75E-03
PDC	phosducin	7.37	6.73E-03
NAMPT	Nicotinamide phosphoribosyltransferase	7.37	2.35E-04
CLDN1	claudin 1	7.36	9.13E-04
TRPC2	transient receptor potential cation channel, subfamily C, member 2, pseudogene	7.34	2.71E-03
ELF5	E74-like factor 5 (ets domain transcription factor)	7.34	1.29E-03
FBXO32	F-box protein 32	7.34	2.61E-04
FBXO32	F-box protein 32	7.30	4.90E-05
NLRP3	NLR family, pyrin domain containing 3	7.27	3.83E-04
FAM125B	family with sequence similarity 125, member B	7.27	8.64E-03
CTSS	cathepsin S	7.26	2.71E-03

FZD5	frizzled family receptor 5	7.25	6.37E-03
ARID5B	AT rich interactive domain 5B (MRF1-like)	7.21	1.64E-03
NCOA7	nuclear receptor coactivator 7	7.20	2.10E-05
PRIC285	peroxisomal proliferator-activated receptor A interacting complex 285	7.19	4.08E-04
DNAH1	dynein, axonemal, heavy chain 1	7.19	2.09E-03
DHX58	DEXH (Asp-Glu-X-His) box polypeptide 58	7.18	1.24E-04
SAMD9	sterile alpha motif domain containing 9	7.15	3.00E-06
FHDC1	FH2 domain containing 1	7.14	3.28E-03
C1orf173	chromosome 1 open reading frame 173	7.14	4.67E-03
TNFSF10	tumor necrosis factor (ligand) superfamily, member 10	7.09	8.30E-05
KCNC1	potassium voltage-gated channel, Shaw-related subfamily, member 1	7.09	7.10E-03
APOL6	apolipoprotein L, 6	7.09	1.88E-04
GATA4	GATA binding protein 4	7.07	3.35E-03
CASS4	Cas scaffolding protein family member 4	7.04	1.03E-04
LYPD6	LY6/PLAUR domain containing 6	7.03	2.14E-04
HIST2H2AA3	histone cluster 2, H2aa3	7.01	7.70E-05
SALL3	sal-like 3 (Drosophila)	7.01	2.99E-03
LOC285043	uncharacterized LOC285043	6.99	3.64E-03
IFIT5	interferon-induced protein with tetratricopeptide repeats 5	6.98	2.09E-04
TMTC1	transmembrane and tetratricopeptide repeat containing 1	6.98	5.39E-04
ACSS1	acyl-CoA synthetase short-chain family member 1	6.97	2.21E-03
CASP1	caspase 1, apoptosis-related cysteine peptidase	6.96	2.00E-06
SLC22A16	solute carrier family 22 (organic cation/carnitine transporter), member 16	6.95	2.80E-05
TNFSF10	tumor necrosis factor (ligand) superfamily, member 10	6.93	2.19E-04
STAT1 , STAT1	signal transducer and activator of transcription 1, 91kDa ; signal transducer and activator of transcription 1, 91kDa	6.92	1.25E-04
P2RY1	purinergic receptor P2Y, G-protein coupled, 1	6.91	4.23E-03
MC2R	melanocortin 2 receptor (adrenocorticotropic hormone)	6.91	2.28E-03
CD74	CD74 molecule, major histocompatibility complex, class II invariant chain	6.89	3.60E-05
FAM26F	family with sequence similarity 26, member F	6.88	1.90E-05
STAT1 , STAT1	signal transducer and activator of transcription 1, 91kDa ; signal transducer and activator of transcription 1, 91kDa	6.87	2.70E-04
GCA	grancalcin, EF-hand calcium binding protein	6.85	5.00E-04
SLC30A4	solute carrier family 30 (zinc transporter), member 4	6.85	4.91E-03
MUC4	mucin 4, cell surface associated	6.83	4.11E-03
C3orf20	chromosome 3 open reading frame 20	6.81	7.83E-04
TRABD2A	TraB domain containing 2A	6.81	9.35E-03
SLC6A20	solute carrier family 6 (proline IMINO transporter), member 20	6.80	2.80E-05
LCN10 , LCN6	lipocalin 10 ; lipocalin 6	6.79	3.60E-05
VIPR1	vasoactive intestinal peptide receptor 1	6.78	8.94E-03
PPM1K	protein phosphatase, Mg2+/Mn2+ dependent, 1K	6.78	8.19E-03
LRIG3	leucine-rich repeats and immunoglobulin-like domains 3	6.73	3.25E-03

MFSD12	major facilitator superfamily domain containing 12	6.72	9.28E-04
APOD	apolipoprotein D	6.70	9.20E-05
DNAH7	dynein, axonemal, heavy chain 7	6.66	8.49E-03
SAMD9L	sterile alpha motif domain containing 9-like	6.66	1.00E-05
C20orf203	chromosome 20 open reading frame 203	6.65	6.73E-03
LCP2	lymphocyte cytosolic protein 2 (SH2 domain containing leukocyte protein of 76kDa)	6.63	2.95E-03
SP110	SP110 nuclear body protein	6.63	2.08E-04
PLXNC1	plexin C1	6.61	1.20E-05
TMEM182	transmembrane protein 182	6.59	4.57E-03
STAT1	signal transducer and activator of transcription 1, 91kDa	6.58	1.00E-06
BAMBI	BMP and activin membrane-bound inhibitor homolog (Xenopus laevis)	6.56	3.70E-05
MEP1B	meprin A, beta	6.56	3.73E-04
C2CD4B	C2 calcium-dependent domain containing 4B	6.54	1.93E-04
ANK2	ankyrin 2, neuronal	6.53	4.83E-03
ACTN2	actinin, alpha 2	6.52	2.68E-03
CEACAM1	carcinoembryonic antigen-related cell adhesion molecule 1 (biliary glycoprotein)	6.50	2.17E-04
ZFC3H1	zinc finger, C3H1-type containing	6.49	6.90E-03
FGF5	fibroblast growth factor 5	6.47	5.80E-05
LOC285401	uncharacterized LOC285401	6.47	7.12E-03
FAM26F	Family with sequence similarity 26, member F	6.46	1.06E-04
TRIM69	Tripartite motif containing 69	6.46	2.59E-03
IGFBP6	insulin-like growth factor binding protein 6	6.45	2.97E-04
CSF1	colony stimulating factor 1 (macrophage)	6.44	8.20E-05
FGA	fibrinogen alpha chain	6.43	6.21E-03
LOC285556	uncharacterized LOC285556	6.43	3.30E-03
RIMS2	regulating synaptic membrane exocytosis 2	6.41	4.29E-04
BCL2L11	BCL2-like 11 (apoptosis facilitator)	6.39	1.96E-03
CXCR7	Chemokine (C-X-C motif) receptor 7	6.38	2.91E-04
LCN2	lipocalin 2	6.37	3.31E-03
OAS2	2'-5'-oligoadenylate synthetase 2, 69/71kDa	6.37	9.10E-05
MSX1	Msh homeobox 1	6.36	4.60E-05
PIK3AP1	phosphoinositide-3-kinase adaptor protein 1	6.36	2.88E-04
LOC646329	uncharacterized LOC646329	6.32	2.86E-03
C10orf91	chromosome 10 open reading frame 91	6.32	1.40E-03
CYP24A1	cytochrome P450, family 24, subfamily A, polypeptide 1	6.31	3.24E-04
C12orf39	chromosome 12 open reading frame 39	6.31	1.59E-03
CXCL16	chemokine (C-X-C motif) ligand 16	6.29	7.00E-06
SLC38A4	solute carrier family 38, member 4	6.27	8.55E-03
CLEC7A	C-type lectin domain family 7, member A	6.25	1.06E-03
IFIH1	Interferon induced with helicase C domain 1	6.23	2.25E-04
MAP3K8	mitogen-activated protein kinase kinase kinase 8	6.23	3.43E-03
SAMHD1	SAM domain and HD domain 1	6.22	2.40E-05
NFKBIA	nuclear factor of kappa light polypeptide gene enhancer in B-cells inhibitor, alpha	6.21	2.40E-05
SAMSN1	SAM domain, SH3 domain and nuclear localization signals 1	6.18	1.89E-04

CSPG5	chondroitin sulfate proteoglycan 5 (neuroglycan C)	6.17	2.45E-03
TMTC1	transmembrane and tetratricopeptide repeat containing 1	6.17	1.26E-04
LRRC19	leucine rich repeat containing 19	6.16	1.68E-04
CLDN1	claudin 1	6.16	3.47E-04
LONRF2	LON peptidase N-terminal domain and ring finger 2	6.15	2.00E-03
TMEM72	transmembrane protein 72	6.15	4.69E-03
KCNT2	potassium channel, subfamily T, member 2	6.12	3.90E-05
IFNA1	interferon, alpha 1	6.10	5.85E-03
CYP2C9	cytochrome P450, family 2, subfamily C, polypeptide 9	6.07	8.38E-03
DGCR12	DiGeorge syndrome critical region gene 12	6.07	2.02E-03
LOC100131392 , TRIM49 , TRIM49C , TRIM49DP , TRIM49L1	tripartite motif-containing protein 49-like protein 1-like ; tripartite motif containing 49 ; tripartite motif containing 49C ; tripartite motif containing 49D, pseudogene ; tripartite motif containing 49-like 1	6.04	3.14E-03
DDX60L	DEAD (Asp-Glu-Ala-Asp) box polypeptide 60-like	6.03	1.00E-05
CD5L	CD5 molecule-like	6.02	7.52E-04
SIX1	SIX homeobox 1	6.02	7.20E-05
SOCS1	suppressor of cytokine signaling 1	5.98	1.25E-03
IQCF4	IQ motif containing F5 pseudogene	5.95	3.63E-03
DDX60	DEAD (Asp-Glu-Ala-Asp) box polypeptide 60	5.94	1.60E-05
TAP1	transporter 1, ATP-binding cassette, sub-family B (MDR/TAP)	5.92	6.26E-04
KCNE3	potassium voltage-gated channel, Isk-related family, member 3	5.92	5.33E-03
BATF3	basic leucine zipper transcription factor, ATF-like 3	5.91	7.68E-04
FGF2	fibroblast growth factor 2 (basic)	5.90	6.51E-04
STAT1 , STAT1	signal transducer and activator of transcription 1, 91kDa ; signal transducer and activator of transcription 1, 91kDa	5.89	4.30E-05
SP110	SP110 nuclear body protein	5.89	4.70E-05
MUC4	mucin 4, cell surface associated	5.88	2.65E-04
NT5C3	5'-nucleotidase, cytosolic III	5.87	6.14E-08
LYPD6	LY6/PLAUR domain containing 6	5.87	1.85E-03
SAT1	spermidine/spermine N1-acetyltransferase 1	5.84	7.00E-06
MTNR1A	melatonin receptor 1A	5.82	3.01E-04
SP110	SP110 nuclear body protein	5.80	1.43E-04
EPPK1	epiplakin 1	5.80	1.02E-03
UBA7	ubiquitin-like modifier activating enzyme 7	5.79	2.30E-05
TFPI2	tissue factor pathway inhibitor 2	5.79	1.50E-05
DNAH6	dynein, axonemal, heavy chain 6	5.78	1.55E-04
IL7R	interleukin 7 receptor	5.77	7.35E-04
KLK13	kallikrein-related peptidase 13	5.76	1.75E-04
CASP1	caspase 1, apoptosis-related cysteine peptidase	5.76	3.80E-05
KIF6	kinesin family member 6	5.76	5.53E-03
OAS1	2'-5'-oligoadenylate synthetase 1, 40/46kDa	5.75	1.76E-04
HSD11B1	hydroxysteroid (11-beta) dehydrogenase 1	5.75	2.04E-03
ART3	ADP-ribosyltransferase 3	5.75	6.95E-04
VSTM2A	V-set and transmembrane domain containing 2A	5.74	7.50E-05
GOLGA3	golgin A3	5.74	6.05E-03

BANK1	B-cell scaffold protein with ankyrin repeats 1	5.73	2.78E-04
NCF4	neutrophil cytosolic factor 4, 40kDa	5.69	4.37E-04
EHF	ets homologous factor	5.69	7.87E-03
PTGS2	prostaglandin-endoperoxide synthase 2 (prostaglandin G/H synthase and cyclooxygenase)	5.68	4.00E-06
HIST1H2BC	histone cluster 1, H2bc	5.68	3.76E-04
SLC7A2	solute carrier family 7 (cationic amino acid transporter, y+ system), member 2	5.66	6.35E-04
IRF1	interferon regulatory factor 1	5.66	1.01E-04
MT1M	metallothionein 1M	5.65	5.68E-04
SAMSN1	SAM domain, SH3 domain and nuclear localization signals 1	5.64	1.69E-03
GXYLT2	glucoside xylosyltransferase 2	5.63	2.62E-04
GMPR	guanosine monophosphate reductase	5.61	9.70E-05
APOL1	apolipoprotein L, 1	5.61	4.00E-06
SLCO3A1	solute carrier organic anion transporter family, member 3A1	5.61	8.61E-03
IFI44	interferon-induced protein 44	5.60	1.60E-05
VNN1	vanin 1	5.60	3.60E-05
JUNB	jun B proto-oncogene	5.58	1.50E-05
IRF1	interferon regulatory factor 1	5.58	5.10E-05
UBA7	ubiquitin-like modifier activating enzyme 7	5.55	6.81E-04
HDAC9	histone deacetylase 9	5.55	1.35E-03
KCTD14 , NDUFC2-KCTD14	potassium channel tetramerisation domain containing 14 ; NDUFC2-KCTD14 readthrough	5.54	2.60E-05
NAMPT	nicotinamide phosphoribosyltransferase	5.52	5.10E-05
TREX1	three prime repair exonuclease 1	5.51	3.21E-04
TNC	tenascin C	5.50	3.97E-03
PDGFRL	platelet-derived growth factor receptor-like	5.50	1.10E-05
KCTD14 , NDUFC2-KCTD14	potassium channel tetramerisation domain containing 14 ; NDUFC2-KCTD14 readthrough	5.50	4.50E-05
FOSL2	FOS-like antigen 2	5.49	1.44E-03
GFRA1	GDNF family receptor alpha 1	5.49	5.52E-03
PHF15	PHD finger protein 15	5.49	7.88E-03
TWIST1	twist homolog 1 (Drosophila)	5.48	2.84E-04
FAM132B	family with sequence similarity 132, member B	5.46	1.10E-04
TAOK1	TAO kinase 1	5.45	7.67E-03
C19orf66	chromosome 19 open reading frame 66	5.44	3.72E-04
CAPN3	calpain 3, (p94)	5.41	4.00E-06
EGR4	early growth response 4	5.40	6.18E-03
HIST1H2BC , HIST1H2BE , HIST1H2BF , HIST1H2BG , HIST1H2BI	histone cluster 1, H2bc ; histone cluster 1, H2be ; histone cluster 1, H2bf ; histone cluster 1, H2bg ; histone cluster 1, H2bi	5.38	6.70E-05
FLCN	folliculin	5.38	2.11E-03
TAPT1	transmembrane anterior posterior transformation 1	5.37	9.66E-03
AKIP1 , NUAK2	A kinase (PRKA) interacting protein 1 ; NUAK family, SNF1-like kinase, 2	5.37	2.80E-05
UBE2QL1	ubiquitin-conjugating enzyme E2Q family-like 1	5.37	6.21E-03
TMC5	transmembrane channel-like 5	5.37	5.57E-03

ZNFX1	zinc finger, NFX1-type containing 1	5.36	3.00E-05
SAMD9L	sterile alpha motif domain containing 9-like	5.36	3.20E-05
TXNIP	thioredoxin interacting protein	5.35	8.00E-06
ALOX5	arachidonate 5-lipoxygenase	5.35	2.20E-05
BLZF1	basic leucine zipper nuclear factor 1	5.35	6.39E-04
LOC100506342	uncharacterized LOC100506342	5.34	5.50E-05
ALS2CR11	amyotrophic lateral sclerosis 2 (juvenile) chromosome region, candidate 11	5.33	2.60E-05
DUOXA1	dual oxidase maturation factor 1	5.33	1.29E-03
PPP1R15A	protein phosphatase 1, regulatory subunit 15A	5.32	3.70E-05
S1PR2	sphingosine-1-phosphate receptor 2	5.32	2.74E-04
GRIK1-AS1	GRIK1 antisense RNA 1 (non-protein coding)	5.32	4.35E-03
RET	ret proto-oncogene	5.31	9.30E-04
CASP1	caspase 1, apoptosis-related cysteine peptidase	5.31	8.74E-04
OR7E91P	olfactory receptor, family 7, subfamily E, member 91 pseudogene	5.31	2.92E-03
LOC100130275	uncharacterized LOC100130275	5.29	5.80E-03
TRIM36	tripartite motif containing 36	5.28	4.81E-04
GADD45B	growth arrest and DNA-damage-inducible, beta	5.26	9.70E-05
TREX1	three prime repair exonuclease 1	5.23	5.00E-04
CCDC146	coiled-coil domain containing 146	5.23	7.57E-03
SLC25A28	solute carrier family 25 (mitochondrial iron transporter), member 28	5.22	2.00E-06
ZBTB16	zinc finger and BTB domain containing 16	5.21	4.00E-05
GZMA	granzyme A (granzyme 1, cytotoxic T-lymphocyte-associated serine esterase 3)	5.20	8.00E-06
BCL6	B-cell CLL/lymphoma 6	5.20	5.27E-03
TSHZ2	teashirt zinc finger homeobox 2	5.20	2.71E-03
RHEBL1	Ras homolog enriched in brain like 1	5.20	1.68E-04
SIX2	SIX homeobox 2	5.18	2.97E-04
SH3RF3-AS1	SH3RF3 antisense RNA 1 (non-protein coding)	5.18	4.30E-05
XRN1	5'-3' exoribonuclease 1	5.18	1.39E-04
FOSB	FBJ murine osteosarcoma viral oncogene homolog B	5.15	3.64E-04
COL14A1	collagen, type XIV, alpha 1	5.15	3.89E-04
PNPT1	polyribonucleotide nucleotidyltransferase 1	5.15	8.20E-05
TRAFD1	TRAF-type zinc finger domain containing 1	5.13	4.30E-05
IQCF1	IQ motif containing F1	5.13	1.27E-03
MIOX	myo-inositol oxygenase	5.10	8.72E-03
PML	promyelocytic leukemia	5.10	1.59E-03
IL8	interleukin 8	5.09	2.10E-04
VSIG10L	V-set and immunoglobulin domain containing 10 like	5.09	2.00E-06
JUP , KRT17	junction plakoglobin ; keratin 17	5.08	2.91E-04
GNG4	guanine nucleotide binding protein (G protein), gamma 4	5.08	4.98E-03
IL10RA	interleukin 10 receptor, alpha	5.07	1.47E-03
CSF1	colony stimulating factor 1 (macrophage)	5.07	3.48E-03
SMR3B	submaxillary gland androgen regulated protein 3B	5.07	8.18E-03
CACNA1A	calcium channel, voltage-dependent, P/Q type, alpha 1A subunit	5.07	2.40E-05
CREB5 , LOC401317	cAMP responsive element binding protein 5 ; uncharacterized LOC401317	5.05	6.53E-04

CLEC7A	C-type lectin domain family 7, member A	5.05	1.31E-03
LOC100507535	uncharacterized LOC100507535	5.03	3.50E-05
NOD2	nucleotide-binding oligomerization domain containing 2	5.02	1.96E-03
NR4A2	nuclear receptor subfamily 4, group A, member 2	5.01	2.07E-03
CTSS	cathepsin S	5.01	1.00E-05
FLJ39739	uncharacterized FLJ39739	5.01	1.22E-04
MYH13	myosin, heavy chain 13, skeletal muscle	5.00	2.76E-03
TRIM38	tripartite motif containing 38	4.97	3.70E-05
ANK2	ankyrin 2, neuronal	4.96	1.13E-03
KCNMB4	potassium large conductance calcium-activated channel, subfamily M, beta member 4	4.96	7.11E-03
RASGRF1	Ras protein-specific guanine nucleotide-releasing factor 1	4.92	1.09E-03
NPTX2	neuronal pentraxin II	4.91	1.09E-03
TXNIP	thioredoxin interacting protein	4.90	8.72E-04
LOC100507000	uncharacterized LOC100507000	4.90	8.38E-03
NFKBIZ	nuclear factor of kappa light polypeptide gene enhancer in B-cells inhibitor, zeta	4.89	2.50E-05
LOC100130950	uncharacterized LOC100130950	4.89	7.63E-03
LRIG1	leucine-rich repeats and immunoglobulin-like domains 1	4.89	3.68E-03
BMP5	bone morphogenetic protein 5	4.87	8.99E-04
SLC1A2	solute carrier family 1 (glial high affinity glutamate transporter), member 2	4.87	8.00E-04
PRND	prion protein 2 (dublet)	4.85	1.50E-03
CD274	CD274 molecule	4.85	5.80E-05
APOL6	apolipoprotein L, 6	4.84	1.68E-04
TP63	tumor protein p63	4.81	2.71E-03
RABGAP1L	RAB GTPase activating protein 1-like	4.81	1.60E-05
IKBKE	inhibitor of kappa light polypeptide gene enhancer in B-cells, kinase epsilon	4.79	5.00E-05
NUDCD2	NudC domain containing 2	4.79	3.83E-03
HIST1H2BC , HIST1H2BE , HIST1H2BF , HIST1H2BG , HIST1H2BI	histone cluster 1, H2bc ; histone cluster 1, H2be ; histone cluster 1, H2bf ; histone cluster 1, H2bg ; histone cluster 1, H2bi	4.76	7.50E-03
PARP10	poly (ADP-ribose) polymerase family, member 10	4.76	1.60E-04
STAT2	signal transducer and activator of transcription 2, 113kDa	4.75	2.12E-03
IL7R	interleukin 7 receptor	4.75	5.36E-04
ZC3H12C	zinc finger CCCH-type containing 12C	4.75	4.87E-04
CRYM	crystallin, mu	4.73	3.36E-04
TFPI2	tissue factor pathway inhibitor 2	4.73	9.80E-05
ABTB2	ankyrin repeat and BTB (POZ) domain containing 2	4.73	7.18E-03
C5orf56	chromosome 5 open reading frame 56	4.73	3.30E-05
WNK3	WNK lysine deficient protein kinase 3	4.72	4.08E-03
H1F0	H1 histone family, member 0	4.71	8.10E-05
CDKL5	cyclin-dependent kinase-like 5	4.71	2.69E-03
NLRC5	NLR family, CARD domain containing 5	4.71	3.86E-04
CXCL2	chemokine (C-X-C motif) ligand 2	4.70	9.00E-06
DCAF7	DDB1 and CUL4 associated factor 7	4.70	5.42E-03

STARD5	StAR-related lipid transfer (START) domain containing 5	4.69	2.20E-05
VCAN	versican	4.69	4.40E-05
STC2	stanniocalcin 2	4.68	5.61E-03
SLC25A37	solute carrier family 25 (mitochondrial iron transporter), member 37	4.67	1.50E-05
CPED1	cadherin-like and PC-esterase domain containing 1	4.67	2.63E-03
NPAS3	neuronal PAS domain protein 3	4.66	1.40E-05
RHOC	Ras homolog family member C	4.66	1.22E-04
PLSCR1	phospholipid scramblase 1	4.65	1.95E-04
GPR137	G protein-coupled receptor 137	4.65	6.77E-03
OSMR	oncostatin M receptor	4.64	8.91E-03
SAMHD1	SAM domain and HD domain 1	4.64	1.81E-04
EPPK1	epiplakin 1	4.63	2.74E-03
LOC100507007	uncharacterized LOC100507007	4.62	7.75E-03
MOCOS	molybdenum cofactor sulfurase	4.61	5.00E-06
LST1	leukocyte specific transcript 1	4.60	2.72E-03
SEZ6L2	seizure related 6 homolog (mouse)-like 2	4.60	8.81E-03
LINC00167	long intergenic non-protein coding RNA 167	4.60	4.24E-03
WISP1	WNT1 inducible signaling pathway protein 1	4.59	5.13E-03
RARRES3	retinoic acid receptor responder (tazarotene induced) 3	4.58	1.48E-04
N4BP1	NEDD4 binding protein 1	4.58	3.00E-06
NEDD9	neural precursor cell expressed, developmentally down-regulated 9	4.58	1.32E-04
CAPN3	calpain 3, (p94)	4.56	1.71E-04
ARID5B	AT rich interactive domain 5B (MRF1-like)	4.56	4.14E-04
TANC2	tetratricopeptide repeat, ankyrin repeat and coiled-coil containing 2	4.55	1.91E-03
KCNAB3	potassium voltage-gated channel, shaker-related subfamily, beta member 3	4.54	5.98E-03
PARP9	poly (ADP-ribose) polymerase family, member 9	4.54	1.05E-04
MCL1	myeloid cell leukemia sequence 1 (BCL2-related)	4.53	9.60E-03
COL6A2	collagen, type VI, alpha 2	4.53	2.56E-03
HLA-J	major histocompatibility complex, class I, J (pseudogene)	4.53	1.03E-03
NUMA1	Nuclear mitotic apparatus protein 1	4.52	3.50E-03
HGF	hepatocyte growth factor (hepapoietin A; scatter factor)	4.51	4.95E-04
PRRC2B	proline-rich coiled-coil 2B	4.51	8.22E-03
IQGAP2	IQ motif containing GTPase activating protein 2	4.50	1.38E-03
BLZF1	basic leucine zipper nuclear factor 1	4.49	2.12E-03
BDNF	brain-derived neurotrophic factor	4.48	4.00E-06
SLC25A37	solute carrier family 25 (mitochondrial iron transporter), member 37	4.46	4.20E-04
MIR155 , MIR155HG	microRNA 155 ; MIR155 host gene (non-protein coding)	4.46	4.60E-05
CEACAM1	carcinoembryonic antigen-related cell adhesion molecule 1 (biliary glycoprotein)	4.45	8.48E-07
FOS	FBJ murine osteosarcoma viral oncogene homolog	4.44	9.56E-03
ABCA6	ATP-binding cassette, sub-family A (ABC1), member 6	4.44	1.18E-04
PAPPA	pregnancy-associated plasma protein A, pappalysin 1	4.44	2.46E-04
CBLN2	cerebellin 2 precursor	4.44	1.40E-05

WTAP	Wilms tumor 1 associated protein	4.43	1.50E-05
HIVEP2	human immunodeficiency virus type I enhancer binding protein 2	4.43	4.25E-04
DCAF4L1	DDB1 and CUL4 associated factor 4-like 1	4.43	2.65E-03
IFIT5	interferon-induced protein with tetratricopeptide repeats 5	4.42	9.00E-06
NUB1	negative regulator of ubiquitin-like proteins 1	4.42	3.02E-03
LOC145663	Uncharacterized LOC145663	4.42	5.73E-04
PPP1R15A	protein phosphatase 1, regulatory subunit 15A	4.41	2.10E-05
PML	promyelocytic leukemia	4.41	7.40E-05
TXNIP	thioredoxin interacting protein	4.40	7.50E-05
DHRS9	dehydrogenase/reductase (SDR family) member 9	4.37	1.60E-05
DHRS9	dehydrogenase/reductase (SDR family) member 9	4.34	3.78E-04
LOC401312	uncharacterized LOC401312	4.32	4.05E-04
LOC100505500	uncharacterized LOC100505500	4.30	3.37E-03
GADD45B	growth arrest and DNA-damage-inducible, beta	4.29	4.00E-06
HLA-G	major histocompatibility complex, class I, G	4.29	3.90E-05
LGALS9	lectin, galactoside-binding, soluble, 9	4.27	8.60E-05
IFNE	interferon, epsilon	4.27	1.74E-03
IRAK3	interleukin-1 receptor-associated kinase 3	4.26	4.35E-04
NPY1R	neuropeptide Y receptor Y1	4.25	8.79E-03
CCDC148	coiled-coil domain containing 148	4.25	7.87E-03
GNG8	guanine nucleotide binding protein (G protein), gamma 8	4.25	9.44E-03
C5orf56	chromosome 5 open reading frame 56	4.24	4.90E-05
SDC4	syndecan 4	4.22	1.85E-04
IL4I1	interleukin 4 induced 1	4.22	1.41E-03
GREM1	gremlin 1	4.21	1.82E-03
SERPINB9	serpin peptidase inhibitor, clade B (ovalbumin), member 9	4.21	8.58E-04
SIRPB2	signal-regulatory protein beta 2	4.21	1.80E-05
FOSL2	FOS-like antigen 2	4.19	6.00E-05
TRIM21	tripartite motif containing 21	4.18	7.20E-05
HCG26	HLA complex group 26 (non-protein coding)	4.18	5.28E-04
CXCL2	Chemokine (C-X-C motif) ligand 2	4.18	8.82E-03
WARS	tryptophanyl-tRNA synthetase	4.16	6.60E-05
LTB	lymphotoxin beta (TNF superfamily, member 3)	4.16	1.95E-03
PMAIP1	phorbol-12-myristate-13-acetate-induced protein 1	4.15	8.52E-04
FNDC8	fibronectin type III domain containing 8	4.15	3.42E-03
C21orf91	chromosome 21 open reading frame 91	4.15	1.56E-04
IL18BP	interleukin 18 binding protein	4.15	3.00E-04
RNF213	ring finger protein 213	4.15	1.34E-04
SLC25A37	Solute carrier family 25 (mitochondrial iron transporter), member 37	4.14	8.00E-06
C3AR1	complement component 3a receptor 1	4.13	1.07E-03
C21orf91	chromosome 21 open reading frame 91	4.12	6.74E-04
PATL1	protein associated with topoisomerase II homolog 1 (yeast)	4.12	1.62E-04
LPAR5	lysophosphatidic acid receptor 5	4.10	1.18E-03
TMEM106A	transmembrane protein 106A	4.10	3.20E-04
CCL23	chemokine (C-C motif) ligand 23	4.09	5.31E-04

EID3	EP300 interacting inhibitor of differentiation 3	4.08	4.70E-03
BLZF1	basic leucine zipper nuclear factor 1	4.07	1.10E-04
EGR1	early growth response 1	4.05	2.11E-04
IL15	interleukin 15	4.05	6.20E-05
USP42	Ubiquitin specific peptidase 42	4.05	8.19E-03
SLC16A4	solute carrier family 16, member 4 (monocarboxylic acid transporter 5)	4.04	2.94E-03
KIAA0226	KIAA0226	4.04	1.09E-04
LOC284561	uncharacterized LOC284561	4.02	1.20E-03
LOC100506377	uncharacterized LOC100506377	4.02	1.00E-05
TDRD7	tudor domain containing 7	4.01	1.10E-05
DHRS9	dehydrogenase/reductase (SDR family) member 9	4.01	1.00E-06
C5orf56	chromosome 5 open reading frame 56	4.01	8.40E-05
TAP2	transporter 2, ATP-binding cassette, sub-family B (MDR/TAP)	4.00	2.47E-04
PARP9	poly (ADP-ribose) polymerase family, member 9	4.00	7.10E-05

Down-regulated genes following RV-infection in HSaVEC

Gene Symbol	Description	Fold Change	ANOVA p-value
PLA2G12A	phospholipase A2, group XIIA	-4.02	2.30E-05
RAB6B	RAB6B, member RAS oncogene family	-4.06	7.33E-03
TXNL1	thioredoxin-like 1	-4.06	6.60E-05
FRY	furry homolog (Drosophila)	-4.10	2.00E-06
MYEF2	myelin expression factor 2	-4.14	7.69E-04
KSR2	kinase suppressor of ras 2	-4.14	5.40E-05
SLC46A1	solute carrier family 46 (folate transporter), member 1	-4.14	6.92E-03
GLS	glutaminase	-4.15	3.60E-05
GNL1	guanine nucleotide binding protein-like 1	-4.15	3.94E-04
CYP4X1	cytochrome P450, family 4, subfamily X, polypeptide 1	-4.16	5.90E-05
LOC100505782	uncharacterized LOC100505782	-4.16	1.68E-03
MAS1	MAS1 oncogene	-4.18	8.62E-03
RPS15A	ribosomal protein S15a	-4.18	3.70E-05
RBBP4	retinoblastoma binding protein 4	-4.18	5.11E-04
PITX3	paired-like homeodomain 3	-4.19	5.15E-03
HTR2B	5-hydroxytryptamine (serotonin) receptor 2B, G protein-coupled	-4.21	8.16E-04
C10orf128	chromosome 10 open reading frame 128	-4.21	3.50E-05
ANKRD29	ankyrin repeat domain 29	-4.23	4.90E-04
RPL27A , SNORA3	ribosomal protein L27a ; small nucleolar RNA, H/ACA box 3	-4.25	2.41E-03
PDK3	pyruvate dehydrogenase kinase, isozyme 3	-4.27	5.48E-03
CD36	CD36 molecule (thrombospondin receptor)	-4.28	1.42E-03
NMU	neuromedin U	-4.32	2.96E-04
RGS5	regulator of G-protein signaling 5	-4.33	6.00E-06
LOC100506343	uncharacterized LOC100506343	-4.33	6.76E-03
ZNF423	zinc finger protein 423	-4.34	1.62E-03
CELF2	CUGBP, Elav-like family member 2	-4.34	3.28E-04
LOC158402	Uncharacterized LOC158402	-4.34	8.04E-04
ZADH2	zinc binding alcohol dehydrogenase domain	-4.35	3.07E-04

	containing 2		
CSRNP3	cysteine-serine-rich nuclear protein 3	-4.38	4.68E-03
SPTBN1	spectrin, beta, non-erythrocytic 1	-4.40	1.02E-04
SYMPK	symplekin	-4.44	3.11E-03
NFIA	nuclear factor I/A	-4.50	3.17E-03
AK5	adenylate kinase 5	-4.51	5.02E-04
FABP4	fatty acid binding protein 4, adipocyte	-4.51	4.54E-03
ZAK	sterile alpha motif and leucine zipper containing kinase AZK	-4.52	5.54E-03
DCLK1	doublecortin-like kinase 1	-4.58	1.61E-03
NPFFR1	neuropeptide FF receptor 1	-4.58	4.14E-03
ENAH	enabled homolog (Drosophila)	-4.58	1.19E-03
FAM212B	family with sequence similarity 212, member B	-4.58	1.25E-04
FBLN1	fibulin 1	-4.59	3.75E-04
LRPAP1	low density lipoprotein receptor-related protein associated protein 1	-4.62	6.16E-03
LINC00304	long intergenic non-protein coding RNA 304	-4.63	1.02E-03
SERPIND1	serpin peptidase inhibitor, clade D (heparin cofactor), member 1	-4.66	1.96E-04
ADAMTS5	ADAM metallopeptidase with thrombospondin type 1 motif, 5	-4.66	4.52E-04
LRRC55	leucine rich repeat containing 55	-4.68	6.48E-03
LOC388796 , SNORA71B	uncharacterized LOC388796 ; small nucleolar RNA, H/ACA box 71B	-4.69	6.85E-03
RGS5	regulator of G-protein signaling 5	-4.72	2.82E-04
CXADR	coxsackie virus and adenovirus receptor	-4.73	1.24E-03
MYRIP	myosin VIIA and Rab interacting protein	-4.75	2.97E-04
OSBPL7	oxysterol binding protein-like 7	-4.77	4.43E-03
MYCN	v-myc myelocytomatosis viral related oncogene, neuroblastoma derived (avian)	-4.78	5.26E-03
IPW , LOC100506948 , SNORD107 , SNORD115-13 , SNORD115-26 , SNORD115-7 , SNORD116-28	imprinted in Prader-Willi syndrome (non-protein coding) ; uncharacterized LOC100506948 ; small nucleolar RNA, C/D box 107 ; small nucleolar RNA, C/D box 115-13 ; small nucleolar RNA, C/D box 115-26 ; small nucleolar RNA, C/D box 115-7 ; small nucleolar RNA, C/D box 116-28	-4.78	3.04E-03
PAMR1	peptidase domain containing associated with muscle regeneration 1	-4.80	2.63E-03
DCLK1	doublecortin-like kinase 1	-4.83	3.07E-04
RGS7BP	regulator of G-protein signaling 7 binding protein	-4.88	5.35E-04
MEG3	maternally expressed 3 (non-protein coding)	-4.92	5.59E-03
MYEF2	myelin expression factor 2	-4.93	6.30E-05
KRT80	keratin 80	-4.93	1.27E-03
DNAH12	dynein, axonemal, heavy chain 12	-4.93	7.49E-03
EMID2	EMI domain containing 2	-4.96	3.16E-04
LOH12CR2	loss of heterozygosity, 12, chromosomal region 2 (non-protein coding)	-5.00	5.36E-03
C4orf17	chromosome 4 open reading frame 17	-5.01	9.58E-04
MYO5B	myosin VB	-5.03	3.47E-03
NOG	noggin	-5.04	4.93E-04
PARD6G	par-6 partitioning defective 6 homolog gamma (C. elegans)	-5.05	8.81E-03
GIPC2	GIPC PDZ domain containing family, member 2	-5.10	6.60E-05
FLRT3	fibronectin leucine rich transmembrane protein 3	-5.17	8.93E-04

CXADR	coxsackie virus and adenovirus receptor	-5.18	7.40E-05
TMEM242	transmembrane protein 242	-5.18	5.59E-03
LOC100506795	uncharacterized LOC100506795	-5.19	3.36E-03
CKAP2 , IGLC1 , IGLV1-44	Cytoskeleton associated protein 2 ; Immunoglobulin lambda constant 1 (Mcg marker) ; Immunoglobulin lambda variable 1-44	-5.23	9.90E-03
CBL	Cbl proto-oncogene, E3 ubiquitin protein ligase	-5.25	3.49E-03
ZMYM2	zinc finger, MYM-type 2	-5.27	1.04E-03
PKI55	DKFZp434H1419	-5.30	1.58E-04
PLA2G12A	phospholipase A2, group XIIA	-5.30	7.45E-03
WDR17	WD repeat domain 17	-5.32	9.13E-03
RHBDL3	Rhomboid, veinlet-like 3 (Drosophila)	-5.34	4.51E-03
ADAL	adenosine deaminase-like	-5.38	3.59E-03
RGS5	regulator of G-protein signaling 5	-5.46	3.85E-04
C7orf41	chromosome 7 open reading frame 41	-5.59	2.06E-03
TMEM170B	transmembrane protein 170B	-5.60	1.54E-03
ARAP3	ArfGAP with RhoGAP domain, ankyrin repeat and PH domain 3	-5.60	4.36E-04
MMP16	matrix metallopeptidase 16 (membrane-inserted)	-5.64	5.70E-05
LINC00466	long intergenic non-protein coding RNA 466	-5.66	7.42E-03
CLIC5	chloride intracellular channel 5	-5.68	2.00E-03
EIF2C1	eukaryotic translation initiation factor 2C, 1	-5.72	3.55E-04
NLN	neurolysin (metallopeptidase M3 family)	-5.73	7.89E-04
TRPM1	transient receptor potential cation channel, subfamily M, member 1	-5.76	9.03E-03
CRLS1	cardiolipin synthase 1	-5.77	1.82E-03
SESN3	sestrin 3	-5.80	1.84E-04
PDK3	pyruvate dehydrogenase kinase, isozyme 3	-5.84	5.51E-04
GHSR	growth hormone secretagogue receptor	-5.92	2.86E-03
C17orf104	chromosome 17 open reading frame 104	-5.96	3.78E-03
SLC7A1	solute carrier family 7 (cationic amino acid transporter, y+ system), member 1	-5.98	3.09E-03
DMRT2	doublesex and mab-3 related transcription factor 2	-5.98	6.50E-03
GTF2H5	general transcription factor IIH, polypeptide 5	-6.00	2.00E-06
ABCD2	ATP-binding cassette, sub-family D (ALD), member 2	-6.04	6.24E-03
CIDEA	cell death-inducing DFFA-like effector a	-6.04	3.54E-04
KLHL4	kelch-like 4 (Drosophila)	-6.09	5.00E-06
PRND	prion protein 2 (dublet)	-6.10	4.32E-04
Dbpht2	DNA binding protein with his-thr domain	-6.16	2.59E-03
LRRC17	leucine rich repeat containing 17	-6.17	1.00E-06
LOC90499	uncharacterized LOC90499	-6.21	6.76E-03
PTPN22	protein tyrosine phosphatase, non-receptor type 22 (lymphoid)	-6.30	3.97E-04
PTPN22	protein tyrosine phosphatase, non-receptor type 22 (lymphoid)	-6.38	5.14E-03
MYCNOS	MYCN opposite strand/antisense RNA (non-protein coding)	-6.42	1.61E-03
CERS5	ceramide synthase 5	-6.50	4.90E-03
ADAM29	ADAM metallopeptidase domain 29	-6.56	5.96E-03
DDX31	DEAD (Asp-Glu-Ala-Asp) box polypeptide 31	-6.61	1.45E-03
SLC12A4	solute carrier family 12 (potassium/chloride transporters), member 4	-6.63	3.05E-03

DUOX1	dual oxidase 1	-6.69	1.37E-03
APOA5	apolipoprotein A-V	-6.80	1.37E-04
C12orf42	chromosome 12 open reading frame 42	-6.82	1.13E-03
AK5	adenylate kinase 5	-6.85	3.35E-04
NALCN	sodium leak channel, non-selective	-6.88	1.10E-05
PCGEM1	prostate-specific transcript 1 (non-protein coding)	-6.88	5.79E-03
FLYWCH1	FLYWCH-type zinc finger 1	-7.02	3.34E-03
TLL2	tolloid-like 2	-7.03	7.77E-04
NDP	Norrie disease (pseudoglioma)	-7.16	8.20E-05
TSPAN11	tetraspanin 11	-7.19	1.29E-04
PDE7A	phosphodiesterase 7A	-7.20	1.65E-03
ADAMTS5	ADAM metallopeptidase with thrombospondin type 1 motif, 5	-7.28	6.52E-04
SESN3	sestrin 3	-7.30	4.58E-03
LOC644090	uncharacterized LOC644090	-7.31	4.15E-03
RGS5	regulator of G-protein signaling 5	-7.34	1.30E-03
ADAMTS5	ADAM metallopeptidase with thrombospondin type 1 motif, 5	-7.47	7.43E-04
PTPN22	protein tyrosine phosphatase, non-receptor type 22 (lymphoid)	-7.49	1.64E-04
LOC100505576	uncharacterized LOC100505576	-7.68	3.40E-03
PRO2012	uncharacterized protein PRO2012	-7.70	1.10E-04
NPHP4	nephronophthisis 4	-7.72	5.46E-04
FXYD4	FXYD domain containing ion transport regulator 4	-7.82	8.93E-03
MGC42157	uncharacterized locus MGC42157	-7.82	7.49E-03
ZNF362	Zinc finger protein 362	-7.88	6.56E-04
DKFZp451A211	uncharacterized LOC400169	-7.91	2.70E-03
TMEM35	transmembrane protein 35	-8.07	3.02E-04
KCNJ1	potassium inwardly-rectifying channel, subfamily J, member 1	-8.17	1.20E-05
CYFIP2	cytoplasmic FMR1 interacting protein 2	-8.18	2.62E-03
C1orf150	chromosome 1 open reading frame 150	-8.21	1.83E-03
BCRP3	breakpoint cluster region pseudogene 3	-8.33	8.10E-05
STRBP	spermatid perinuclear RNA binding protein	-8.36	3.00E-06
FZD3	frizzled family receptor 3	-8.56	3.65E-03
TACC1	Transforming, acidic coiled-coil containing protein 1	-8.67	2.16E-04
ACO1	aconitase 1, soluble	-8.77	6.82E-07
APLNR	apelin receptor	-9.07	5.51E-04
OR8B8	olfactory receptor, family 8, subfamily B, member 8	-9.24	4.78E-04
KCNJ4	potassium inwardly-rectifying channel, subfamily J, member 4	-9.80	1.15E-03
KRT19	keratin 19	-10.41	4.60E-05
SEMA6A	sema domain, transmembrane domain (TM), and cytoplasmic domain, (semaphorin) 6A	-10.76	2.83E-04
SESN3	sestrin 3	-10.85	6.92E-04
RPS6KA6	ribosomal protein S6 kinase, 90kDa, polypeptide 6	-10.92	2.71E-03
PPFIBP2	PTPRF interacting protein, binding protein 2 (liprin beta 2)	-11.34	8.28E-03
CAMSAP1	calmodulin regulated spectrin-associated protein 1	-11.66	9.95E-03
KRT19	Keratin 19	-11.76	4.00E-06
C18orf34	chromosome 18 open reading frame 34	-12.66	8.00E-06

ZMAT4	zinc finger, matrin-type 4	-12.80	4.14E-04
MRAP2	melanocortin 2 receptor accessory protein 2	-13.37	3.70E-05
CD36	CD36 molecule (thrombospondin receptor)	-13.70	1.64E-04
FAM13C	family with sequence similarity 13, member C	-14.25	3.47E-03
AQP1	aquaporin 1 (Colton blood group)	-15.64	2.62E-03
CXADR	coxsackie virus and adenovirus receptor	-15.68	2.95E-03
GAMT	guanidinoacetate N-methyltransferase	-19.83	6.52E-03
DMRT1	doublesex and mab-3 related transcription factor 1	-20.61	2.70E-05
REEP1	receptor accessory protein 1	-26.32	5.80E-03
AGXT2L1	alanine-glyoxylate aminotransferase 2-like 1	-27.20	8.86E-03
SPINK13	serine peptidase inhibitor, Kazal type 13 (putative)	-30.27	3.88E-03
CLEC4GP1	C-type lectin domain family 4, member G pseudogene 1	-40.63	1.44E-03
