# Supplementary material for: Gene expression profiling of rubella virus infected primary endothelial cells of fetal and adult origin
Source: Virol J. 2016 Feb 2;13:21. doi: 10.1186/s12985-016-0475-9 (PMC4736114; doi:10.1186/s12985-016-0475-9)

Additional File 2. Absolute chemokine expression following RV infection. HUVEC and HSaVEC were infected with RV at an MOI of 10 and the cell culture supernatant was collected 48 hpi. Non-infected cells served as control. Chemokine levels in the supernatant were determined using a human chemokine array kit. Data are represented as the mean pixel intensity of two spots ± SD of one representative assay.

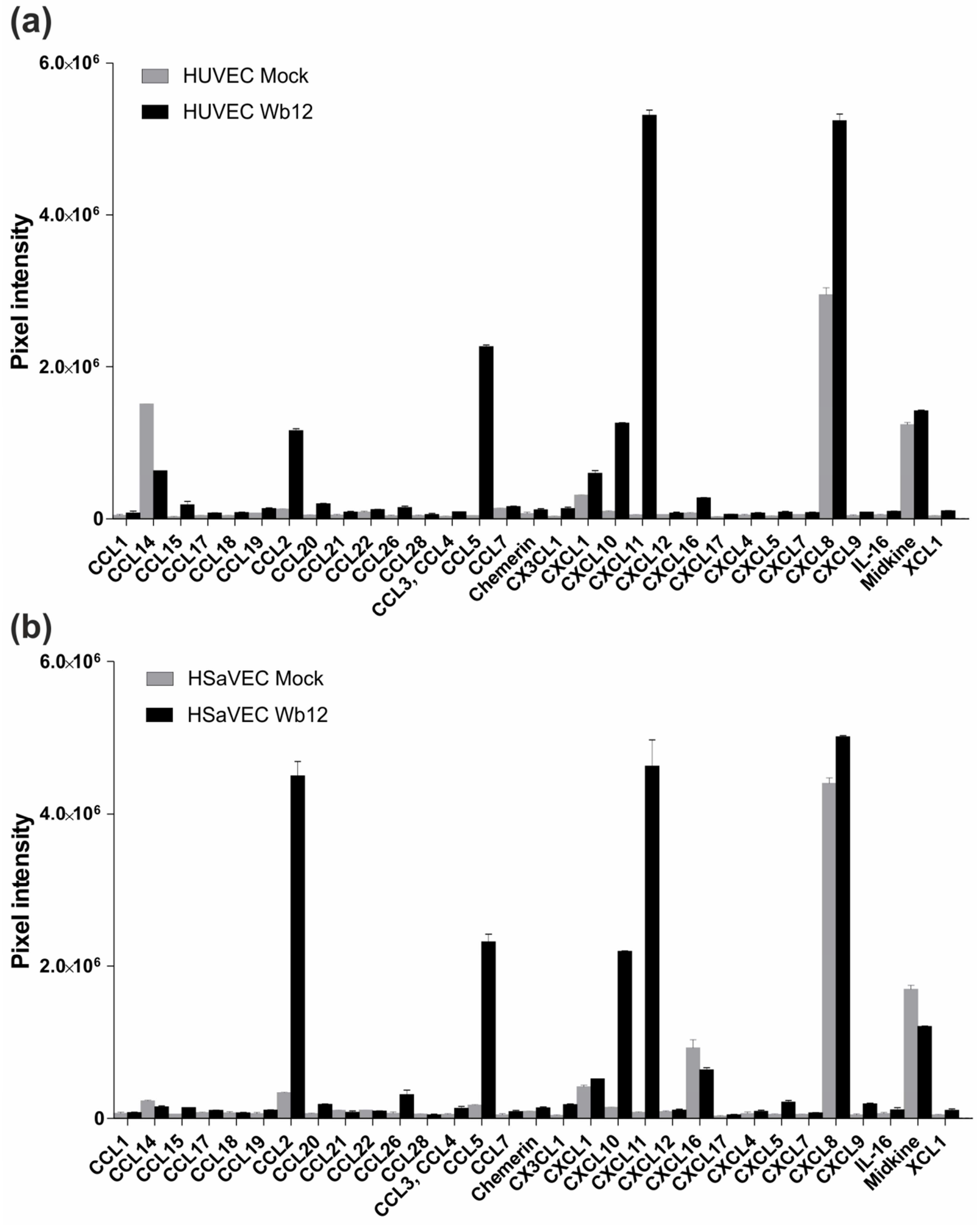

Supplement: Additional file 2: — Absolute chemokine expression following RV infection. (PDF 761 kb) [file 12985_2016_475_MOESM2_ESM.pdf]
