# Supplementary material for: Gene expression profiling of rubella virus infected primary endothelial cells of fetal and adult origin
Source: Virol J. 2016 Feb 2;13:21. doi: 10.1186/s12985-016-0475-9 (PMC4736114; doi:10.1186/s12985-016-0475-9)
Supplement: Additional file 3: — Ranking of commonly up- and down-regulated genes in the endothelial cells following RV infection. (PDF 52 kb) [file 12985_2016_475_MOESM3_ESM.pdf]

Additional File 3. Ranking of commonly up- and down-regulated genes in the endothelial cells following RV infection. The mean of the log-fold changes of infected HUVEC and HSaVEC was calculated, anti-logged (FC) and ranked according to expression level for the 344 and 48 commonly up- and down-regulated genes.

Commonly up-regulated genes following RV infection			
**Rank**	**Gene Symbol**	**Description**	**FC**
**1**	IFNB1	interferon, beta 1, fibroblast	4309.47
**2**	CXCL10	chemokine (C-X-C motif) ligand 10	1199.70
**3**	SCN3A	sodium channel, voltage-gated, type III, alpha subunit	1117.52
**4**	MX2	myxovirus (influenza virus) resistance 2 (mouse)	872.20
**5**	CCL5	chemokine (C-C motif) ligand 5	562.25
**6**	IL28A	interleukin 28A (interferon, lambda 2)	480.18
**7**	CCL4	chemokine (C-C motif) ligand 4	474.07
**8**	OASL	2'-5'-oligoadenylate synthetase-like	458.75
**9**	TNFAIP6	tumor necrosis factor, alpha-induced protein 6	445.77
**10**	IDO1	indoleamine 2,3-dioxygenase 1	429.65
**11**	RSAD2	radical S-adenosyl methionine domain containing 2	336.58
**12**	CCL8	chemokine (C-C motif) ligand 8	296.17
**13**	C3	complement component 3	259.16
**14**	TNFSF13B	tumor necrosis factor (ligand) superfamily, member 13b	254.32
**15**	AIM2	absent in melanoma 2	239.08
**16**	CCL3	chemokine (C-C motif) ligand 3 ; chemokine (C-C motif) ligand 3-like 1 ; chemokine (C-C motif) ligand 3-like 3	232.32
**17**	CSAG2	CSAG family, member 2 ; CSAG family, member 3	221.73
**18**	IFIT1	interferon-induced protein with tetratricopeptide repeats 1	200.44
**19**	ELOVL7	ELOVL fatty acid elongase 7	185.82
**20**	RTP4	receptor (chemosensory) transporter protein 4	147.32
**21**	IRG1	immunoresponsive 1 homolog (mouse)	145.68
**22**	ATP10A	ATPase, class V, type 10A	138.14
**23**	SECTM1	secreted and transmembrane 1	124.02
**24**	CD38	CD38 molecule	123.20
**25**	IL12RB1	interleukin 12 receptor, beta 1	121.21
**26**	CCL20	chemokine (C-C motif) ligand 20	117.08
**27**	TNIP3	TNFAIP3 interacting protein 3	116.28
**28**	TAC3	tachykinin 3	113.21
**29**	STATH	statherin	109.75
**30**	IFI44L	interferon-induced protein 44-like	106.47
**31**	C1S	complement component 1, s subcomponent	104.38
**32**	TLR2	toll-like receptor 2	99.35
**33**	HERC5	HECT and RLD domain containing E3 ubiquitin protein ligase 5	98.49
**34**	BTC	betacellulin	95.79
**35**	IFIT2	interferon-induced protein with tetratricopeptide repeats 2	91.18
**36**	IFIT3	interferon-induced protein with tetratricopeptide repeats 3	89.61
**37**	SIDT1	SID1 transmembrane family, member 1	86.76
**38**	IL29	interleukin 29 (interferon, lambda 1)	86.60
**39**	NEURL3	neuralized homolog 3 (Drosophila) pseudogene	83.54
**40**	IFI30	interferon, gamma-inducible protein 30	78.46
**41**	EPSTI1	epithelial stromal interaction 1 (breast)	74.36
**42**	CR1	complement component (3b/4b) receptor 1 (Knops blood group) ;	71.84

		complement component (3b/4b) receptor 1-like	
**43**	GABBR1	gamma-aminobutyric acid (GABA) B receptor, 1 ; ubiquitin D	71.46
**44**	CMPK2	cytidine monophosphate (UMP-CMP) kinase 2, mitochondrial	71.44
**45**	TNFRSF9	tumor necrosis factor receptor superfamily, member 9	70.83
**46**	SLC15A3	solute carrier family 15, member 3	69.70
**47**	HSH2D	hematopoietic SH2 domain containing	69.69
**48**	TEX14	testis expressed 14	69.61
**49**	CR1L	complement component (3b/4b) receptor 1-like	68.46
**50**	ISG20	interferon stimulated exonuclease gene 20kDa	68.25
**51**	GBP4	guanylate binding protein 4	67.74
**52**	PI3	peptidase inhibitor 3, skin-derived	65.92
**53**	CXCL11	chemokine (C-X-C motif) ligand 11	65.40
**54**	GBP5	guanylate binding protein 5	64.79
**55**	TSLP	thymic stromal lymphopoietin	63.01
**56**	CCL7	chemokine (C-C motif) ligand 7	61.23
**57**	CXCL9	chemokine (C-X-C motif) ligand 9	60.72
**58**	IFITM1	interferon induced transmembrane protein 1	60.40
**59**	BATF2	basic leucine zipper transcription factor, ATF-like 2	57.21
**60**	MX1	myxovirus (influenza virus) resistance 1, interferon-inducible protein p78 (mouse)	56.96
**61**	SERPINA3	serpin peptidase inhibitor, clade A (alpha-1 antiproteinase, antitrypsin), member 3	56.31
**62**	CXCL3	chemokine (C-X-C motif) ligand 3	55.51
**63**	PDZK1IP1	PDZK1 interacting protein 1	54.00
**64**	ANGPTL1	angiopoietin-like 1	53.76
**65**	RNF175	ring finger protein 175	53.72
**66**	IL1RN	interleukin 1 receptor antagonist	52.54
**67**	SAA1	serum amyloid A1 ; serum amyloid A2 ; SAA2-SAA4 readthrough	49.50
**68**	SELE	selectin E	49.00
**69**	C1R	complement component 1, r subcomponent	46.78
**70**	KLRD1	killer cell lectin-like receptor subfamily D, member 1	45.10
**71**	KYNU	kynureninase	44.84
**72**	ADAM28	ADAM metallopeptidase domain 28	43.25
**73**	S100P	S100 calcium binding protein P	43.22
**74**	VCAM1	vascular cell adhesion molecule 1	42.84
**75**	P2RY6	pyrimidinergic receptor P2Y, G-protein coupled, 6	41.44
**76**	IFI6	interferon, alpha-inducible protein 6	40.91
**77**	CSF3	colony stimulating factor 3 (granulocyte)	38.44
**78**	CSF2	colony stimulating factor 2 (granulocyte-macrophage)	38.37
**79**	IFI35	interferon-induced protein 35	37.85
**80**	TNFAIP3	tumor necrosis factor, alpha-induced protein 3	37.16
**81**	SLC1A3	solute carrier family 1 (glial high affinity glutamate transporter), member 3	36.49
**82**	FAM65B	family with sequence similarity 65, member B	36.36
**83**	ETV7	ets variant 7	35.60
**84**	LGALS3BP	lectin, galactoside-binding, soluble, 3 binding protein	35.07
**85**	BDKRB2	bradykinin receptor B2	34.61
**86**	CFB	complement factor B	34.30
**87**	TMEM229B	transmembrane protein 229B	34.27
**88**	BST2	bone marrow stromal cell antigen 2	33.82

**89**	NCF2	neutrophil cytosolic factor 2	33.46
**90**	TBC1D1	TBC1 (tre-2/USP6, BUB2, cdc16) domain family, member 1	33.27
**91**	KLK10	kallikrein-related peptidase 10	32.80
**92**	BIRC3	baculoviral IAP repeat containing 3	30.61
**93**	LIF	leukemia inhibitory factor	30.45
**94**	EGR3	early growth response 3	29.96
**95**	LOC100129518	uncharacterized LOC100129518	29.59
**96**	GPR84	G protein-coupled receptor 84	28.64
**97**	CXCL6	chemokine (C-X-C motif) ligand 6 (granulocyte chemotactic protein 2)	27.49
**98**	LRRN3	leucine rich repeat neuronal 3	26.55
**99**	SGPP2	sphingosine-1-phosphate phosphatase 2	26.08
**100**	CLDN23	claudin 23	25.76
**101**	MAB21L2	mab-21-like 2 (C. elegans)	25.35
**102**	CXCL5	chemokine (C-X-C motif) ligand 5	24.97
**103**	C15orf48	chromosome 15 open reading frame 48	24.64
**104**	IL1B	interleukin 1, beta	24.27
**105**	C8orf34	chromosome 8 open reading frame 34	24.08
**106**	CLEC4E	C-type lectin domain family 4, member E	24.02
**107**	IFIH1	Interferon induced with helicase C domain 1	23.96
**108**	SSTR2	somatostatin receptor 2	23.76
**109**	CX3CL1	chemokine (C-X3-C motif) ligand 1	23.53
**110**	BANCR	BRAF-activated non-protein coding RNA	23.35
**111**	NR4A3	nuclear receptor subfamily 4, group A, member 3	23.23
**112**	TMEM139	transmembrane protein 139	22.76
**113**	CTSS	cathepsin S	22.68
**114**	HCG4	HLA complex group 4 (non-protein coding)	22.19
**115**	HERC6	HECT and RLD domain containing E3 ubiquitin protein ligase family member 6	21.95
**116**	LRP2	low density lipoprotein receptor-related protein 2	21.89
**117**	CH25H	cholesterol 25-hydroxylase	21.75
**118**	CD69	CD69 molecule	21.52
**119**	MRGPRX3	MAS-related GPR, member X3	21.31
**120**	ICAM1	intercellular adhesion molecule 1	21.15
**121**	PTGS2	prostaglandin-endoperoxide synthase 2 (prostaglandin G/H synthase and cyclooxygenase)	20.37
**122**	ODF3B	outer dense fiber of sperm tails 3B	19.67
**123**	CYP1B1	cytochrome P450, family 1, subfamily B, polypeptide 1	19.66
**124**	OAS2	2'-5'-oligoadenylate synthetase 2, 69/71kDa	19.36
**125**	TLR3	toll-like receptor 3	19.23
**126**	PIK3AP1	phosphoinositide-3-kinase adaptor protein 1	18.87
**127**	APOBEC3G	apolipoprotein B mRNA editing enzyme, catalytic polypeptide-like 3G	18.83
**128**	F3	coagulation factor III (thromboplastin, tissue factor)	18.27
**129**	4SEP	septin 4	18.23
**130**	ATF3	activating transcription factor 3	18.00
**131**	PSMB9	proteasome (prosome, macropain) subunit, beta type, 9 (large multifunctional peptidase 2)	17.93
**132**	NKX3-1	NK3 homeobox 1	17.88
**133**	C2	complement component 2	17.83
**134**	APOL6	apolipoprotein L, 6	17.72

**135**	PLEKHA4	pleckstrin homology domain containing, family A (phosphoinositide binding specific) member 4	17.59
**136**	ISG15	ISG15 ubiquitin-like modifier	17.56
**137**	OAS1	2'-5'-oligoadenylate synthetase 1, 40/46kDa	17.45
**138**	GBP1	guanylate binding protein 1, interferon-inducible	17.42
**139**	FAM65C	family with sequence similarity 65, member C	16.94
**140**	IL6	interleukin 6 (interferon, beta 2)	16.82
**141**	DENND2D	DENN/MADD domain containing 2D	16.78
**142**	PLA1A	phospholipase A1 member A	16.73
**143**	XAF1	XIAP associated factor 1	16.22
**144**	FCGR2A	Fc fragment of IgG, low affinity IIa, receptor (CD32)	15.93
**145**	BRE-AS1	BRE antisense RNA 1 (non-protein coding)	15.58
**146**	DSP	desmoplakin	15.44
**147**	RET	ret proto-oncogene	15.28
**148**	C6orf58	Chromosome 6 open reading frame 58	14.94
**149**	IL18RAP	interleukin 18 receptor accessory protein	14.81
**150**	OAS3	2'-5'-oligoadenylate synthetase 3, 100kDa	14.78
**151**	DDX58	DEAD (Asp-Glu-Ala-Asp) box polypeptide 58	14.59
**152**	EDNRA	endothelin receptor type A	14.13
**153**	CD83	CD83 molecule	13.86
**154**	SAMD9	sterile alpha motif domain containing 9	13.63
**155**	RARRES3	retinoic acid receptor responder (tazarotene induced) 3	13.31
**156**	SLC22A16	solute carrier family 22 (organic cation/carnitine transporter), member 16	13.29
**157**	CASP1	caspase 1, apoptosis-related cysteine peptidase	13.16
**158**	PDE6H	phosphodiesterase 6H, cGMP-specific, cone, gamma	13.15
**159**	CEBPD	CCAAT/enhancer binding protein (C/EBP), delta	13.07
**160**	PRIC285	peroxisomal proliferator-activated receptor A interacting complex 285	13.04
**161**	LY6E	lymphocyte antigen 6 complex, locus E	12.83
**162**	HCP5	HLA complex P5 (non-protein coding)	12.80
**163**	FAM46A	Family with sequence similarity 46, member A	12.77
**164**	CYP2J2	cytochrome P450, family 2, subfamily J, polypeptide 2	12.70
**165**	ZC3H12A	zinc finger CCCH-type containing 12A	12.45
**166**	IL1A	interleukin 1, alpha	12.23
**167**	TNC	tenascin C	12.10
**168**	PDZD2	PDZ domain containing 2	12.08
**169**	ZBTB32	zinc finger and BTB domain containing 32	12.00
**170**	SYNPO2	synaptopodin 2	11.88
**171**	FST	follistatin	11.82
**172**	ACTN2	actinin, alpha 2	11.80
**173**	HAS2	hyaluronan synthase 2	11.71
**174**	PPM1K	protein phosphatase, Mg2+/Mn2+ dependent, 1K	11.60
**175**	COL1A1	collagen, type I, alpha 1	11.48
**176**	VNN3	vanin 3	11.35
**177**	SOX8	SRY (sex determining region Y)-box 8	11.32
**178**	HIST2H2BE	histone cluster 2, H2be	11.30
**179**	APOL1	apolipoprotein L, 1	11.18
**180**	SLAMF8	SLAM family member 8	10.99
**181**	PRRG4	proline rich Gla (G-carboxyglutamic acid) 4 (transmembrane)	10.97

**182**	SAMD9L	sterile alpha motif domain containing 9-like	10.94
**183**	KIAA0146	KIAA0146	10.71
**184**	IL18BP	interleukin 18 binding protein	10.57
**185**	LOC285628	uncharacterized LOC285628	10.55
**186**	MXD1	MAX dimerization protein 1	10.52
**187**	KLF4	Kruppel-like factor 4 (gut)	10.50
**188**	FXYD6	FXYD domain containing ion transport regulator 6	10.47
**189**	IRF7	interferon regulatory factor 7	10.40
**190**	TRIM14	tripartite motif containing 14	10.34
**191**	IL8	interleukin 8	10.33
**192**	TRAF1	TNF receptor-associated factor 1	10.24
**193**	CLEC7A	C-type lectin domain family 7, member A	10.15
**194**	ARAP2	ArfGAP with RhoGAP domain, ankyrin repeat and PH domain 2	10.01
**195**	RASGRF1	Ras protein-specific guanine nucleotide-releasing factor 1	10.00
**196**	PARP10	poly (ADP-ribose) polymerase family, member 10	9.98
**197**	LOC100271840	uncharacterized LOC100271840	9.95
**198**	USP18	ubiquitin specific peptidase 18	9.94
**199**	MAP3K8	mitogen-activated protein kinase kinase kinase 8	9.88
**200**	IL12A	interleukin 12A (natural killer cell stimulatory factor 1, cytotoxic lymphocyte maturation factor 1, p35)	9.87
**201**	ZC3HAV1	zinc finger CCCH-type, antiviral 1	9.81
**202**	IL3RA	interleukin 3 receptor, alpha (low affinity)	9.76
**203**	HSD11B1	hydroxysteroid (11-beta) dehydrogenase 1	9.73
**204**	LCP2	lymphocyte cytosolic protein 2 (SH2 domain containing leukocyte protein of 76kDa)	9.68
**205**	TNFSF10	tumor necrosis factor (ligand) superfamily, member 10	9.64
**206**	IFI44	interferon-induced protein 44	9.55
**207**	PARP8	poly (ADP-ribose) polymerase family, member 8	9.51
**208**	SIK1	salt-inducible kinase 1	9.25
**209**	TFAP2A	transcription factor AP-2 alpha (activating enhancer binding protein 2 alpha)	9.24
**210**	COL8A1	collagen, type VIII, alpha 1	9.19
**211**	SOCS1	suppressor of cytokine signaling 1	9.18
**212**	AKIP1	A kinase (PRKA) interacting protein 1 ; NUAK family, SNF1-like kinase, 2	9.11
**213**	IRF8	interferon regulatory factor 8	9.05
**214**	MUC4	mucin 4, cell surface associated	9.02
**215**	DDX60	DEAD (Asp-Glu-Ala-Asp) box polypeptide 60	8.96
**216**	CASZ1	castor zinc finger 1	8.95
**217**	LOC284561	uncharacterized LOC284561	8.84
**218**	TAP1	transporter 1, ATP-binding cassette, sub-family B (MDR/TAP)	8.78
**219**	TNFAIP2	tumor necrosis factor, alpha-induced protein 2	8.74
**220**	SAMHD1	SAM domain and HD domain 1	8.72
**221**	HIST2H2AA3	histone cluster 2, H2aa3	8.56
**222**	FBXO6	F-box protein 6	8.51
**223**	MT1M	metallothionein 1M	8.46
**224**	GPR65	G protein-coupled receptor 65	8.37
**225**	SP110	SP110 nuclear body protein	8.34
**226**	HLA-G	major histocompatibility complex, class I, G	8.31
**227**	SRD5A3-AS1	SRD5A3 antisense RNA 1 (non-protein coding)	8.31
**228**	PTPRC	protein tyrosine phosphatase, receptor type, C	8.30

**229**	FBXO32	F-box protein 32	8.26
**230**	CXCL2	chemokine (C-X-C motif) ligand 2	8.25
**231**	PKIB	protein kinase (cAMP-dependent, catalytic) inhibitor beta	8.24
**232**	DHX58	DEXH (Asp-Glu-X-His) box polypeptide 58	8.21
**233**	CLDN14	claudin 14	8.18
**234**	NLRP3	NLR family, pyrin domain containing 3	8.08
**235**	RNF213	ring finger protein 213	8.04
**236**	JAK3	Janus kinase 3	7.97
**237**	LOC285556	uncharacterized LOC285556	7.97
**238**	GXYLT2	glucoside xylosyltransferase 2	7.93
**239**	IFNA1	interferon, alpha 1	7.93
**240**	MSX1	Msh homeobox 1	7.88
**241**	GZMA	granzyme A (granzyme 1, cytotoxic T-lymphocyte-associated serine esterase 3)	7.74
**242**	SLC7A2	solute carrier family 7 (cationic amino acid transporter, y+ system), member 2	7.72
**243**	PCLO	piccolo (presynaptic cytomatrix protein)	7.60
**244**	C12orf39	chromosome 12 open reading frame 39	7.48
**245**	NFKBIZ	nuclear factor of kappa light polypeptide gene enhancer in B-cells inhibitor, zeta	7.44
**246**	CXCL16	chemokine (C-X-C motif) ligand 16	7.39
**247**	ACHE	acetylcholinesterase	7.38
**248**	GMPR	guanosine monophosphate reductase	7.36
**249**	PLSCR1	phospholipid scramblase 1	7.36
**250**	STAT1	signal transducer and activator of transcription 1, 91kDa	7.33
**251**	CEACAM1	carcinoembryonic antigen-related cell adhesion molecule 1 (biliary glycoprotein)	7.33
**252**	NCOA7	nuclear receptor coactivator 7	7.27
**253**	FAM26F	family with sequence similarity 26, member F	7.22
**254**	DDX60L	DEAD (Asp-Glu-Ala-Asp) box polypeptide 60-like	7.21
**255**	LAMA2	laminin, alpha 2	7.18
**256**	KCTD14	potassium channel tetramerisation domain containing 14 ; NDUFC2-KCTD14 readthrough	7.08
**257**	PMAIP1	phorbol-12-myristate-13-acetate-induced protein 1	7.07
**258**	BATF3	basic leucine zipper transcription factor, ATF-like 3	7.05
**259**	BANK1	B-cell scaffold protein with ankyrin repeats 1	7.04
**260**	PAPPA	pregnancy-associated plasma protein A, pappalysin 1	7.03
**261**	GJD3	gap junction protein, delta 3, 31.9kDa	6.97
**262**	NFKBIA	nuclear factor of kappa light polypeptide gene enhancer in B-cells inhibitor, alpha	6.95
**263**	IRF1	interferon regulatory factor 1	6.92
**264**	LGALS9	lectin, galactoside-binding, soluble, 9	6.88
**265**	NPAS3	neuronal PAS domain protein 3	6.79
**266**	XRN1	5'-3' exoribonuclease 1	6.74
**267**	IGFBP6	insulin-like growth factor binding protein 6	6.70
**268**	NLRC5	NLR family, CARD domain containing 5	6.69
**269**	HDAC9	histone deacetylase 9	6.69
**270**	VNN1	vanin 1	6.68
**271**	C2CD4B	C2 calcium-dependent domain containing 4B	6.61
**272**	ZFP36	zinc finger protein 36, C3H type, homolog (mouse)	6.61
**273**	TRIM69	tripartite motif containing 69	6.60
**274**	KIAA0664L3	KIAA0664-like 3	6.55

**275**	RHEBL1	Ras homolog enriched in brain like 1	6.52
**276**	FGF2	fibroblast growth factor 2 (basic)	6.51
**277**	CSF1	colony stimulating factor 1 (macrophage)	6.50
**278**	WISP1	WNT1 inducible signaling pathway protein 1	6.49
**279**	HLA-J	major histocompatibility complex, class I, J (pseudogene)	6.48
**280**	STAT2	signal transducer and activator of transcription 2, 113kDa	6.43
**281**	SLC1A2	solute carrier family 1 (glial high affinity glutamate transporter), member 2	6.32
**282**	IFIT5	interferon-induced protein with tetratricopeptide repeats 5	6.32
**283**	TFPI2	tissue factor pathway inhibitor 2	6.23
**284**	UBA7	ubiquitin-like modifier activating enzyme 7	6.22
**285**	CLDN1	claudin 1	6.22
**286**	NAMPT	Nicotinamide phosphoribosyltransferase	6.15
**287**	MOCOS	molybdenum cofactor sulfurase	6.09
**288**	MFSD12	major facilitator superfamily domain containing 12	6.05
**289**	CXCR7	chemokine (C-X-C motif) receptor 7	6.04
**290**	SLC16A4	solute carrier family 16, member 4 (monocarboxylic acid transporter 5)	5.95
**291**	CACNA1A	calcium channel, voltage-dependent, P/Q type, alpha 1A subunit	5.93
**292**	APOD	apolipoprotein D	5.76
**293**	GCA	grancalcin, EF-hand calcium binding protein	5.75
**294**	ZNFX1	zinc finger, NFX1-type containing 1	5.73
**295**	FLJ39739	uncharacterized FLJ39739	5.72
**296**	SAT1	spermidine/spermine N1-acetyltransferase 1	5.72
**297**	CAPN3	calpain 3, (p94)	5.69
**298**	JUNB	jun B proto-oncogene	5.63
**299**	STARD5	StAR-related lipid transfer (START) domain containing 5	5.58
**300**	SLC38A4	solute carrier family 38, member 4	5.54
**301**	TRIM21	tripartite motif containing 21	5.51
**302**	SH3RF3-AS1	SH3RF3 antisense RNA 1 (non-protein coding)	5.50
**303**	TAP2	transporter 2, ATP-binding cassette, sub-family B (MDR/TAP)	5.50
**304**	PDGFRL	platelet-derived growth factor receptor-like	5.47
**305**	C19orf66	chromosome 19 open reading frame 66	5.47
**306**	WARS	tryptophanyl-tRNA synthetase	5.38
**307**	PARP9	poly (ADP-ribose) polymerase family, member 9	5.37
**308**	C3AR1	complement component 3a receptor 1	5.37
**309**	LST1	leukocyte specific transcript 1	5.32
**310**	TRIM38	tripartite motif containing 38	5.31
**311**	SAMSN1	SAM domain, SH3 domain and nuclear localization signals 1	5.29
**312**	PNPT1	polyribonucleotide nucleotidyltransferase 1	5.27
**313**	TDRD7	tudor domain containing 7	5.24
**314**	LOC100507535	uncharacterized LOC100507535	5.23
**315**	LOC100507535	uncharacterized LOC100507535	5.23
**316**	LTB	lymphotoxin beta (TNF superfamily, member 3)	5.16
**317**	TXNIP	thioredoxin interacting protein	5.14
**318**	NT5C3	5'-nucleotidase, cytosolic III	5.12
**319**	IL7R	interleukin 7 receptor	5.12
**320**	NOD2	nucleotide-binding oligomerization domain containing 2	5.08
**321**	IFNE	interferon, epsilon	5.06
**322**	SLC25A28	solute carrier family 25 (mitochondrial iron transporter), member	5.04

		28	
**323**	C5orf56	chromosome 5 open reading frame 56	4.96
**324**	MIR155	microRNA 155 ; MIR155 host gene (non-protein coding)	4.96
**325**	FOSL2	FOS-like antigen 2	4.96
**326**	RHOC	Ras homolog family member C	4.96
**327**	PML	promyelocytic leukemia	4.95
**328**	TREX1	three prime repair exonuclease 1	4.93
**329**	BLZF1	basic leucine zipper nuclear factor 1	4.92
**330**	VSIG10L	V-set and immunoglobulin domain containing 10 like	4.91
**331**	S1PR2	sphingosine-1-phosphate receptor 2	4.81
**332**	NEDD9	neural precursor cell expressed, developmentally down-regulated 9	4.66
**333**	TRAFD1	TRAF-type zinc finger domain containing 1	4.61
**334**	NR4A2	nuclear receptor subfamily 4, group A, member 2	4.59
**335**	N4BP1	NEDD4 binding protein 1	4.50
**336**	ZC3H12C	zinc finger CCCH-type containing 12C	4.50
**337**	TMEM106A	transmembrane protein 106A	4.47
**338**	PATL1	protein associated with topoisomerase II homolog 1 (yeast)	4.45
**339**	CD274	CD274 molecule	4.43
**340**	IRAK3	interleukin-1 receptor-associated kinase 3	4.34
**341**	IL15	interleukin 15	4.30
**342**	IL4I1	interleukin 4 induced 1	4.30
**343**	SLC25A37	solute carrier family 25 (mitochondrial iron transporter), member 37	4.28
**344**	CCL23	chemokine (C-C motif) ligand 23	4.20

Commonly down-regulated genes following RV infection

**Rank**	**Gene Symbol**	**Description**	**FC**
**1**	REEP1	receptor accessory protein 1	-28.58
**2**	CLEC4GP1	C-type lectin domain family 4, member G pseudogene 1	-19.91
**3**	AQP1	aquaporin 1 (Colton blood group)	-19.88
**4**	MRAP2	melanocortin 2 receptor accessory protein 2	-16.13
**5**	STRBP	spermatid perinuclear RNA binding protein	-14.06
**6**	C18orf34	chromosome 18 open reading frame 34	-12.89
**7**	SEMA6A	sema domain, transmembrane domain (TM), and cytoplasmic domain, (semaphorin) 6A	-12.53
**8**	NALCN	sodium leak channel, non-selective	-11.72
**9**	FAM212B	family with sequence similarity 212, member B	-10.40
**10**	CD36	CD36 molecule (thrombospondin receptor)	-10.39
**11**	TMEM35	transmembrane protein 35	-10.32
**12**	ACO1	aconitase 1, soluble	-10.26
**13**	FZD3	frizzled family receptor 3	-9.25
**14**	TSPAN11	tetraspanin 11	-8.63
**15**	KRT19	Keratin 19	-8.19
**16**	CXADR	coxsackie virus and adenovirus receptor	-7.62
**17**	RPL27A	ribosomal protein L27a ; small nucleolar RNA, H/ACA box 3	-7.46
**18**	ZMYM2	zinc finger, MYM-type 2	-7.37
**19**	SESN3	sestrin 3	-6.95
**20**	NLN	neurolysin (metallopeptidase M3 family)	-6.80

**21**	C10orf128	chromosome 10 open reading frame 128	-6.69
**22**	TRPM1	transient receptor potential cation channel, subfamily M, member 1	-6.67
**23**	MMP16	matrix metallopeptidase 16 (membrane-inserted)	-6.49
**24**	LRRC17	leucine rich repeat containing 17	-6.40
**25**	CYFIP2	cytoplasmic FMR1 interacting protein 2	-6.20
**26**	KRT80	keratin 80	-5.88
**27**	NOG	noggin	-5.80
**28**	PKI55	DKFZp434H1419	-5.78
**29**	TMEM170B	transmembrane protein 170B	-5.47
**30**	PDK3	pyruvate dehydrogenase kinase, isozyme 3	-5.44
**31**	KLHL4	kelch-like 4 (Drosophila)	-5.36
**32**	SPTBN1	spectrin, beta, non-erythrocytic 1	-5.34
**33**	CRLS1	cardiolipin synthase 1	-5.28
**34**	PARD6G	par-6 partitioning defective 6 homolog gamma (C. elegans)	-5.19
**35**	LRPAP1	low density lipoprotein receptor-related protein associated protein 1	-5.19
**36**	C7orf41	chromosome 7 open reading frame 41	-5.17
**37**	FABP4	fatty acid binding protein 4, adipocyte	-5.13
**38**	AK5	adenylate kinase 5	-5.13
**39**	EIF2C1	eukaryotic translation initiation factor 2C, 1	-5.06
**40**	IPW	imprinted in Prader-Willi syndrome (non-protein coding) ; uncharacterized LOC100506948 ; small nucleolar RNA, C/D box 107 ; small nucleolar RNA, C/D box 115-13 ; small nucleolar RNA, C/D box 115-26 ; small nucleolar RNA, C/D box 115-7 ; small nucleolar RNA, C/D box 116-28	-4.80
**41**	ENAH	enabled homolog (Drosophila)	-4.75
**42**	KSR2	kinase suppressor of ras 2	-4.71
**43**	GIPC2	GIPC PDZ domain containing family, member 2	-4.59
**44**	RGS7BP	regulator of G-protein signaling 7 binding protein	-4.59
**45**	SERPIND1	serpin peptidase inhibitor, clade D (heparin cofactor), member 1	-4.51
**46**	GNL1	guanine nucleotide binding protein-like 1	-4.39
**47**	RPS15A	ribosomal protein S15a	-4.21
**48**	RAB6B	RAB6B	-4.03
