# Supplementary material for: Gene expression profiling of rubella virus infected primary endothelial cells of fetal and adult origin
Source: Virol J. 2016 Feb 2;13:21. doi: 10.1186/s12985-016-0475-9 (PMC4736114; doi:10.1186/s12985-016-0475-9)
Supplement: Additional file 4: — Comparison of differentially regulated genes following RV infection in HUVEC, HSaVEC, HEF and Hs888Lu. (PDF 244 kb) [file 12985_2016_475_MOESM4_ESM.pdf]

Additional File 4. Comparison of differentially regulated genes following RV infection in HUVEC, HSaVEC, HEF and Hs888Lu. (a) Venn diagram showing the intersections between the up- and down-regulated gene lists of HUVEC and HSaVEC (this study) and of HEF and Hs888Lu (1). Commonly regulated genes are depicted in the overlapping area and the number of commonly regulated genes is indicated. The figure is modified from VENNY(2). **(b)** Biological processes of the 86 transcripts that were affected after RV infection in both HUVEC and HEF. Displayed are the ranked Bonferroni corrected p-values of significantly enriched biological processes and pathways ($p \leq 0.01$). Related terms were grouped and the most significant term of the group was defined as the group leading term. Numbers at the bar represent genes (in %) from the cluster that were associated with the term.

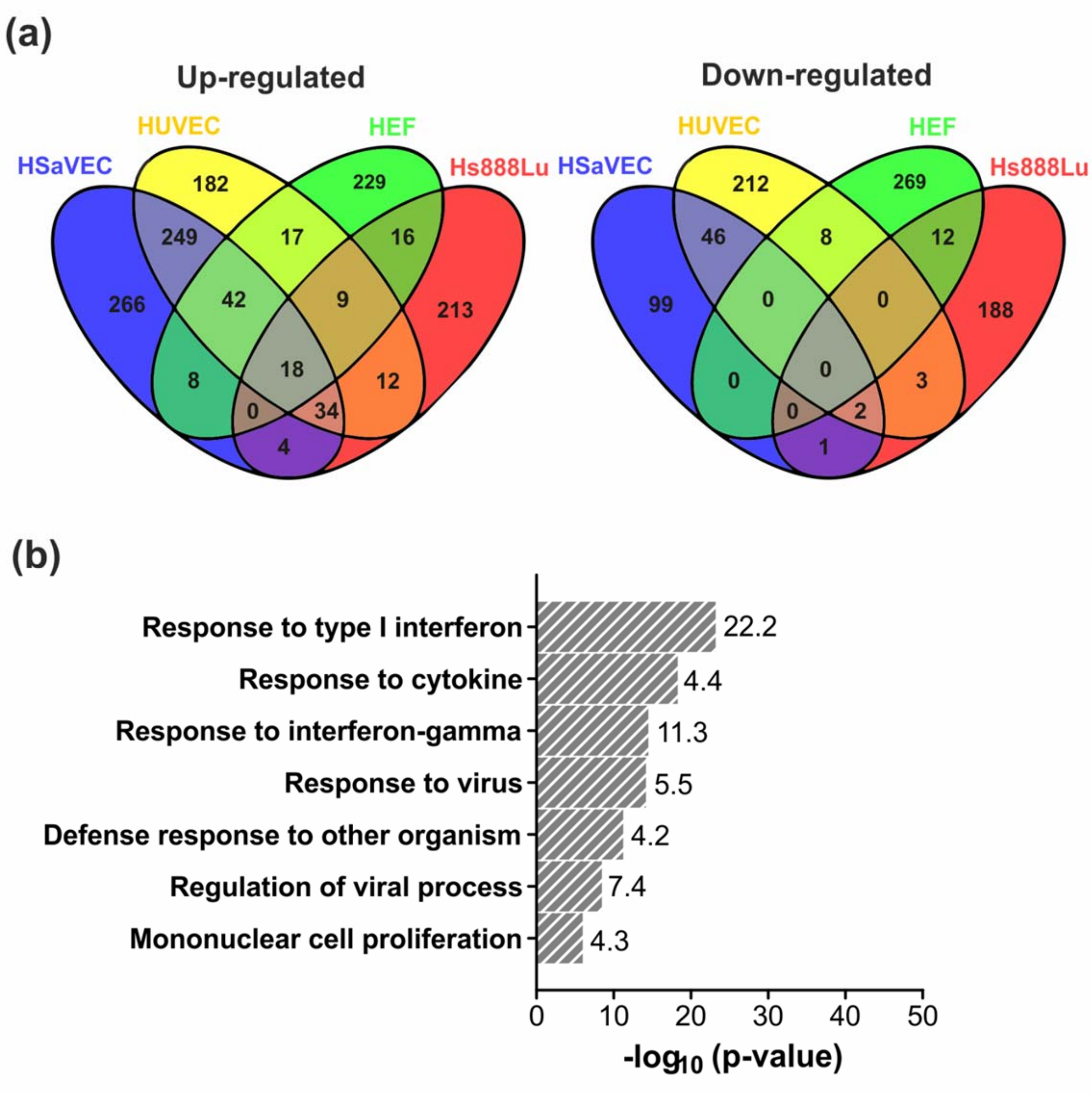

1. **Adamo MP, Zapata M, Frey TK.** 2008. Analysis of gene expression in fetal and adult cells infected with rubella virus. Virology **370:**1-11.
2. **Oliveros JC.** 2007-2015. An interactive tool for comparing lists with Venn's diagrams. Accessed
