# Supplementary material for: Gene expression profiling of rubella virus infected primary endothelial cells of fetal and adult origin
Source: Virol J. 2016 Feb 2;13:21. doi: 10.1186/s12985-016-0475-9 (PMC4736114; doi:10.1186/s12985-016-0475-9)
Supplement: Additional file 5: — List of primer sequences used for expression analysis of genes belonging to the GO term “sensory organ development” by qPCR. (PDF 7 kb) [file 12985_2016_475_MOESM5_ESM.pdf]

Additional File 5. List of primer sequences used for expression analysis of genes belonging to the GO term "sensory organ development" by qPCR.

Gene		Primer sequence	Product size (bp)
ADAMTS18	F	5'-CGCTGGTCTTTGAAATTCTG-3'	160
	R	5'-TAACCTCCACCACAGGAGAC-3'	
ALDH1A2	F	5'-GGATTGCCAAGGAGGAGATC-3'	144
	R	5'-CTGTGAGGGCCTTGTTGATG-3'	
CLN8	F	5'-TCCTGGATGCTCTTAAAGGC-3'	89
	R	5'-ATGCGGCAGTGAAACATGTG-3'	
FGFR2	F	5'-GGCCAACACTGTCAAGTTTC-3'	125
	R	5'-GCTGGTTTCGTACCTTGTAG-3'	
FZD3	F	5'-GAGGATGTGCCAAGATTTGC-3'	135
	R	5'-CGGAAATCCCGAGAACAATC-3'	
JAG2	F	5'-GCCTGCTCTGTGACAAAGAC-3'	147
	R	5'-CGTGCTCAGCCTTCTCACAG-3'	
MYO7A	F	5'-CCAGTGCTGCATCATCAGTG-3'	155
	R	5'-TGGCATTCCCAAATGCTTCC-3'	
NHS	F	5'-TCCCATCAATGTTACTGGAG-3'	92
	R	5'-TTCGTCTCCGCTGTAGTACC-3'	
RDH10	F	5'-ACACTTCTGGACCACTAAGG-3'	123
	R	5'-CTGGCACAGTAATCCTCAAC-3'	
SLC25A27	F	5'-GTATTCTGGAGGTCGAATGG-3'	102
	R	5'-CCTCCAATGACTGATTTCCAAAG-3'	
TNPO1	F	5'-TCCCTTTACTTGAGTGCCTATC-3'	125
	R	5'-TAGCATGGCTTGTGCAAGAG-3'	
TSPAN12	F	5'-CTGCAGAAACGAGGGTAGAG-3'	124
	R	5'-TTCACCGTTCCACAATATCC-3'	
GAPDH	F	5'-TCTTCCAGGAGCGAGATCCC-3'	147
	R	5'-AGGGGGCAGAGATGATGAC-3'	
